# Comprehensive Review of COVID-19: Epidemiology, Pathogenesis, Advancement in Diagnostic and Detection Techniques, and Post-Pandemic Treatment Strategies

**DOI:** 10.3390/ijms25158155

**Published:** 2024-07-26

**Authors:** Yiu-Sing Chung, Ching-Yin Lam, Pak-Hei Tan, Hin-Fung Tsang, Sze-Chuen Cesar Wong

**Affiliations:** Department of Applied Biology & Chemical Technology, The Hong Kong Polytechnic University, Hong Kong, China; yiusing011212@gmail.com (Y.-S.C.); chingyinlam869@gmail.com (C.-Y.L.); pakheitan@gmail.com (P.-H.T.); andy_thf@yahoo.com.hk (H.-F.T.)

**Keywords:** SARS-CoV-2, COVID-19, post-pandemic, endemic, review

## Abstract

At present, COVID-19 remains a public health concern due to the ongoing evolution of SARS-CoV-2 and its prevalence in particular countries. This paper provides an updated overview of the epidemiology and pathogenesis of COVID-19, with a focus on the emergence of SARS-CoV-2 variants and the phenomenon known as ‘long COVID’. Meanwhile, diagnostic and detection advances will be mentioned. Though many inventions have been made to combat the COVID-19 pandemic, some outstanding ones include multiplex RT-PCR, which can be used for accurate diagnosis of SARS-CoV-2 infection. ELISA-based antigen tests also appear to be potential diagnostic tools to be available in the future. This paper also discusses current treatments, vaccination strategies, as well as emerging cell-based therapies for SARS-CoV-2 infection. The ongoing evolution of SARS-CoV-2 underscores the necessity for us to continuously update scientific understanding and treatments for it.

## 1. Introduction

Since the peak of the COVID-19 pandemic, SARS-CoV-2 infectivity and associated mortality rates have decreased. However, society is still at risk from the growing number of new SARS-CoV-2 variants, such as KP.2, KP.3, and LB.1 [1]. Furthermore, a considerable number of individuals are now experiencing prolonged COVID-19 symptoms even after they have recovered from the acute phase of the disease. As a result, COVID-19 remains a public health problem. Despite the early development of detection and diagnostic tests, treatments, and vaccines for this pandemic, the emergence of novel SARS-CoV-2 variants may make these technologies less efficient. This paper provides an up-to-date overview of the progression of COVID-19, along with the development of diagnostic methods, detection methods, and treatment strategies. Continuous updates on the understanding of SARS-CoV-2 are ubiquitous for keeping the current endemic situation under control.

## 2. Epidemiology

### 2.1. Coronavirus

Coronavirus disease 2019 (COVID-19) is caused by the infection of a novel coronavirus (SARS-CoV-2), which is a positive-sense single-stranded RNA virus [2]. Coronavirus is split into four genres: Alpha (α), Beta (β), Gamma (γ), and Delta (δ). The Alpha and Beta subtypes are clinically relevant, as they can cause infectious diseases in humans, while the Gamma and Delta subtypes mainly cause infections in birds [3]. The Alpha and Beta subtypes originated from bats, while the Gamma and Delta subtypes originated from birds [4]. The first human coronavirus was recorded back in the mid-1960s. To date, there are a total of seven types of coronaviruses that can infect humans. Common human coronaviruses include 229E and NL63, which belong to Alpha coronavirus, and the OC43 and HKU1, which are found under Beta coronavirus [5,6,7]. They are viruses that can cause mild upper respiratory tract infections [8]. Meanwhile, there are other types of human coronavirus that can cause severe respiratory tract infections. They are MERS-CoV, SARS-CoV, and SARS-CoV-2, which are all Beta coronaviruses [9,10].

### 2.2. Origin of COVID-19

The first COVID-19 outbreak dates back to December 2019, when a cluster of unexplainable cases of pneumonia were reported in the hospitals in Wuhan, Hubei Province, China. The first reported case was from the Huanan Wet Seafood Wholesale Market in Wuhan, and it then started spreading all over the world. Initially, viral pneumonia was suspected to be the cause [11]. It was later identified to be caused by a novel coronavirus and named severe acute respiratory syndrome coronavirus 2 (SARS-CoV-2) [12]. SARS-CoV-2 was named after SARS-CoV due to close genetics and evolutionary histories [13], and SARS-CoV is the cause of the SARS outbreak that happened in China during 2002–2003 [14]. COVID-19 was first thought to be a zoonotic disease, as the first outbreak of SARS-CoV was spread from animals to humans [15]. Scientists thought SARS-CoV’s transmission source was similar to that of SARS-CoV-2. Most of the COVID-19 patients in early 2020 had visited the fish and wild animal market, which sold live animals such as snakes and bats [16]. Therefore, direct contact and consumption of wild animals were suspected to be the transmission route [17]. As a result, the Chinese government’s first response was to shut down animal wet markets noted for their poor hygiene [18]. However, the infection kept spreading rapidly worldwide. Later, the World Health Organization (WHO) declared COVID-19 a global health emergency and treated it as a pandemic in March 2020.

**Figure 1:**
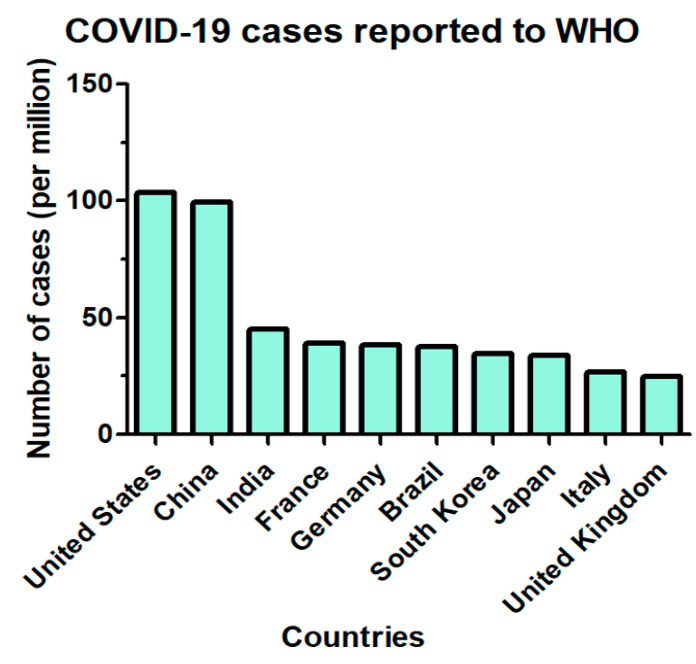
Top 10 countries with the highest number of cases reported to the WHO, cumulatively (as of 13 April 2024).

What is the impact of COVID-19 on global health? Figure 1 shows the top 10 countries with the most reported COVID-19 cases to the WHO as of 13 April 2024 [19]. As shown in the figure, the three most populous countries in the world had the highest numbers of reported COVID-19 cases. The most populated country in the world is India, and it had over 45 million cases of COVID-19, ranking third in the list [19]. The second most populated country is China, and it had over 99 million cases of COVID-19, ranking second in the list [19]. The third most populated country is the US, and it had over 103 million cases of COVID-19, ranking first in the list [19]. These three countries occupy over 35% of the total COVID-19 cases in the world. At the end of 2023, there were over 700 million cases of infection and 7 million deaths worldwide.

### 2.3. Routes of Transmission

The three major types of SARS-CoV-2 transmission are direct contact or indirect contact (via fomites), small airborne droplets (aerosols), and large droplets.

The first type of transmission is through direct or indirect contact. SARS-CoV-2 affects both the upper and lower respiratory tract. In terms of direct contact, SARS-CoV-2 can be transmitted by contacting the mucous membrane of the eyes, ears, or mouth of a COVID-19 patient [20]. Hospital-acquired infections by this transmission method were common among healthcare providers during the COVID-19 pandemic period [21]. Healthcare providers during the COVID-19 outbreak were the most susceptible to this type of transmission as they had to take care of COVID-19 patients [22]. For indirect contact, SARS-CoV-2 is spread through fomites that carry infectious viral particles [23]. The infectious viral particles spread from an infected person and can attach to any surface of inanimate objects. If there is another individual who touches the contaminated surface and brings the infectious viral particles to the mucous membrane of the eyes, ears, or mouth, he or she may be infected by SARS-CoV-2 [24].

The second type of transmission is by aerosols. Aerosols refer to suspensions of small particles in the air filled with the pathogen. Aerosols can travel through the air and enter the mucous membrane of the eyes, ears, and mouth, causing an infection. In a closed environment, aerosols can persist in the air for a long time [25]. SARS-CoV-2 is viable in aerosols for at least 3 h [26]. However, long-range transmissions by aerosols can be regulated by ventilation or air filtration. The aerosols are spread by the expired air from symptomatic patients through coughs and sneezes [27]. Asymptomatic COVID-19 patients may also spread SARS-CoV-2 by producing aerosols that contain infectious SARS particles [28,29]. If they perform activities that involve exhalation, such as talking, singing, or breathing, large quantities of aerosols may be produced [30]. These infectious aerosols can remain in the air for a long time if ventilation is not present.

The third type of transmission is by large droplets. Large droplets harboring SARS-CoV-2 virions can be produced upon coughing or sneezing of a COVID-19 patient. These large droplets can infect any susceptible host if ingested or inhaled [22]. Since large droplets only travel a short distance, maintaining a minimum distance of 1.5 m from an infectious patient can prevent the transmission of SARS-CoV-2 by droplets [31].

### 2.4. SARS-CoV-2 Variant

Since SARS-CoV-2 is an RNA virus, it has a high error rate during viral replication [32]. It causes mutations to happen and results in the formation of new SARS-CoV-2 variants. Table 1 shows the five major variants of concern during 2020–2022. Each variant of SARS-CoV-2 increases its infectivity and has different implications. For example, the Delta variant showed increased evasiveness against neutralizing antibodies found in convalescent patients [33].

Although new variants of SARS-CoV-2, such as XBB.1, BA.2.86.1, and JN.1, continue to arise, the severity and mortality caused by COVID-19 have decreased compared to the start of the pandemic [35]. The use of the vaccines reduced the severity and mortality of COVID-19 [36]. People have developed immunity against SARS-CoV-2 artificially through vaccination or naturally through recovery from the disease. Vaccination helps to lower the mortality rate of COVID-19 and, hence, the mortality rate has continued to decline since the early pandemic stage [37]. Moreover, diagnostic methods and new treatment strategies help prevent the spread and lower the severity of COVID-19.

On 5 May 2023, the director general of the WHO characterized COVID-19 as an ingrained and ongoing health issue, no longer seen as a Public Health Emergency of International Concern [38]. This indicated that the management of COVID-19 had switched toward long-term monitoring and management. With every passing year, more new variants of SARS-CoV-2 emerge. As of 5 June 2024, there are three variants of interest (VOI) noted by the WHO. They are EG.5, BA.2.86, and JN.1, which all originated from the primary Pango Lineage of Omicron (B.1.1.529). Compared to the 2020–2022 period, 2023 and 2024 had no Omicron variant that was listed as a variant of concern (VOC), which by definition requires major public health interventions [39]. VOCs have potential mutations that can cause changes in virulence and infectivity and affect vaccine effectiveness. Also, VOCs can potentially spread in a community and cause transmission across multiple countries [39]. Most sub-lineages of Omicron infect the upper respiratory tract, as opposed to the lower respiratory tracts for the previous VOCs [39]. Lower respiratory tract infection is generally more severe than upper respiratory tract infection, since lower respiratory tract infection may affect lung functions in the case of pneumonia [40]. 

New Omicron variants predicted to be dominant in the US are KP.2, KP.3, and LB.1, and they all emerged from JN.1 [1]. KP.2 and KP.3 are also listed as variants under monitoring, which means these variants may mutate and lead to changes in virulence, infectivity, and affect vaccine effectiveness [39]. The Centers for Disease Control and Prevention (CDC) in the US predicts KP.2, KP.3, and LB.1 to be the three most dominant variants of SARS-CoV-2 [1]. They were expected to account for over 70% of the infection cases over June [1]. In terms of genetic mutation, KP.2 and KP.3 have acquired a new substitution of spike protein, while LB.1 and KP.2.3 acquired a deletion at the 31st position in addition to the previous substitution on spike protein [41]. Scientists have performed Lentivirus-based pseudo-virus assays and neutralization assays to characterize the virological features of KP.2, KP.3, and LB.1 [41]. Pseudo-viruses are recombinant viruses. KP.2 and KP.3 showed increased immune evasion compared to their parental lineage JN.1 because of the newly acquired substitution [41]. LB.1 and KP.2.3 showed enhanced immune evasion and greater infectivity because of the deletion [41].

### 2.5. SARS-CoV-2 Updated Infection Rate and Mortality across Different Regions

**Figure 2:**
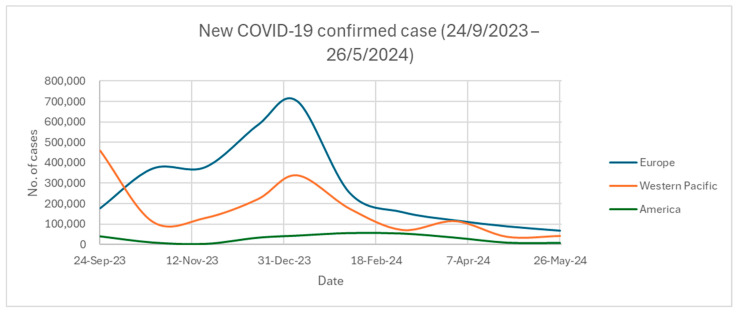
New COVID-19-confirmed cases from 24 September 2023 to 26 May 2024.

**Figure 3:**
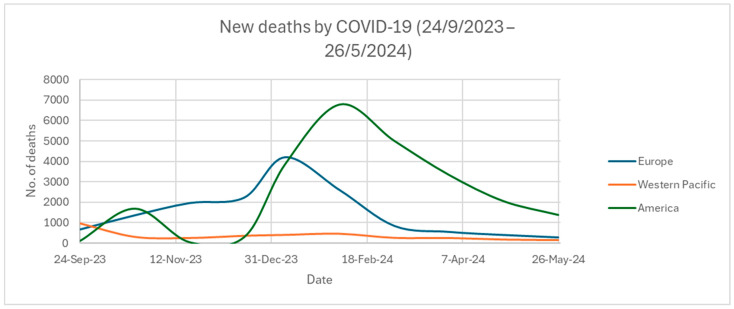
New deaths caused by COVID-19 from 24 September 2023 to 26 May 2024.

Figure 2 and Figure 3 show the newly confirmed COVID-19 cases and mortality rates across the three different WHO regions from 24 September 2023 to 26 May 2024 [42]. The WHO has classified the world into six WHO regions for analysis. They are Africa, America, Eastern Mediterranean, Europe, Southeast Asia, and Western Pacific. All data are collected and counted every month from the WHO. Only the top three WHO regions with the highest COVID-19 cases and deaths are shown [42]. According to Figure 2, Europe and the Western Pacific in the period of 24 September 2023 to 26 May 2024 had many more COVID-19-confirmed cases compared to America. The rise of new COVID-19 cases was either concentrated at the end of the year or the start of a new year. As shown in Figure 3, America had the highest number of deaths when compared to Europe or the Western Pacific, although it had far fewer COVID-19-confirmed cases than the latter two regions. Most of the deaths in the American region were contributed by the US. Over the last 28 days from 19 May to 16 June 2024, 1800 people died around the world, with 1200 deaths originating from the US [43]. However, only 22.5% of adults (above 18 years old) in the US have received the 2023–2024 updated COVID-19 vaccines, such as from Pfizer-BioNTech, Moderna, or Novavax [44]. In comparison, Portugal had the most deaths (164 deaths) in the Europe region from 19 May to 16 June 2024 [43]. Portugal has over 40% of the elderly (aged at least 60 years old) receiving the new COVID-19 vaccines [45]. For the elderly over 80 years old in Portugal, over 60% of them have received the new COVID-19 vaccines [45]. Although the elderly may have higher chances to contract COVID-19 due to having weaker immune systems, Portugal has much lower death rates than the US. This is because of the differences in receiving the new COVID-19 vaccines, especially for the elderly. The US had 10,811 infected cases while Portugal had 7398 infected cases from 19 May to 16 June 2024 [1,46]. The mortality rate from 19 May to 16 June 2024 was 11.10% (1200/10,811) for the US and 2.22% (164/7398) for Portugal. Vaccination offers protection against death from COVID-19, as indicated by the differences in vaccination and mortality rates between the US and Portugal.

### 2.6. SARS-CoV-2 New Transmission Pattern

**Figure 4:**
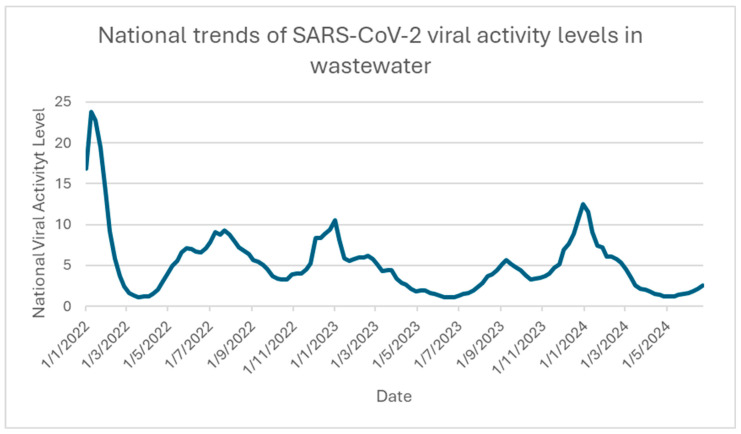
National trends of SARS-CoV-2 viral activity levels in wastewater from the US (accessed on 26 June 2024).

Wastewater viral activity is continuously monitored in the US. It is used to detect infectious diseases, such as COVID-19, and acts as an early warning [47]. Figure 4 depicts the trend of SARS-CoV-2 viral activity levels in wastewater from the US. A baseline was calculated using the 10th percentile of the log-transformed data collected from the wastewater sampling site [47]. The calculation is the number of standard deviations above the baseline, transformed to the linear scale [47]. The viral activity level is considered high when higher than 4.5 and very high when above 8 [47]. As shown in Figure 4, the wastewater viral activity surged at the beginning of 2022 and then fell off. It started to peak again in late summer and dropped again. It then began another surge during the end of the year and the start of the next year [47]. In this sense, COVID-19 infection is probably more common during late summer and the end of the year. With the rise of the new Omicron variants, a new surge is assumed to occur in the late summer, around August and September of 2024 [47].

### 2.7. SARS-CoV-2 Reinfection Rate

With new mutations arising from different variants of SARS-CoV-2, enhanced infectivity and the ability of immune escape of variants can cause reinfection, particularly with the Omicron variants [48]. Reinfection refers to the scenario whereby an individual has obtained their first positive COVID-19 testing result followed by another positive COVID-19 testing result after at least 90 days from the date of the first positive testing result. The reinfection rate varies among different variants of SARS-CoV-2, and the combined reinfection rate across different variants is around 0.94% [49]. The reinfection rate of Omicron variants has been considerably increased compared to all of the previous variants [50]. Whereas less than a 0.6% reinfection rate was found before the emergence of Omicron variants, Omicron variants have a 4.1% reinfection rate [49].

- United States

In the State of New York, from January 2021 to August 2023, there were over 6 million cumulative first infections and over 660 thousand cumulative reinfections [51]. The reinfection rate was 9.8%. The reinfection rate peaked in December 2021, which was probably due to the emergence of the SARS-CoV-2 Omicron variant.

- United Kingdom

In the United Kingdom, a study was conducted across multiple Omicron variant waves [52]. It found that from April 2020 to March 2023, over 96.7% of the first reinfections were caused by various Omicron variants, including BA.1, BA.2, BA.4/5, and BQ.1/CH1.1/XBB.1.5 [52]. The percentage of the second reinfection caused by various Omicron variants further increased to 99.6%, and all third reinfections were caused by the Omicron variants [52]. The reinfection was also found to be less symptomatic, and a lower viral load was found in the patients [52].

- Austria

In Austria, from June 2020 to December 2023, serological screenings were conducted to find out the reinfection rate of COVID-19 [53]. A 1.7% rate of reinfection was found during the Alpha and Beta periods, 4.4% of reinfection was found in the Delta variant period, and 38.5% of reinfection was found in the Omicron variant periods [53]. The reinfection rate started to decline at the end of 2023 when EG.5 was the dominant SARS-CoV-2 variant, as compared to the beginning of the Omicron variant period, where BA.1, BA.2, BA.4/5, and XBB were the dominant SARS-CoV-2 variants [53].

- Mexico

In Mexico, from March 2020 to February 2023, clinical data for COVID-19 were collected from patients who were treated in Mexico [54]. The reinfection rate peaked at 40% in the Omicron variant period, where BA.2 and BA.4/5 were the dominant SARS-CoV-2 variants at the time [54]. In all the reinfection cases, 40% of the reinfection occurred in unvaccinated patients, while 36.4% of reinfection occurred in patients who had received two doses of vaccines [54]. Patients who had received additional vaccine doses only accounted for 20.1% of the reinfection cases [54]. This indicates that vaccination lowers the risks of reinfection when compared to non-vaccination.

As the general public has gained immunity against COVID-19 by either vaccination or recovery from infection over the previous years of the COVID-19 pandemic, COVID-19 has now transitioned from the pandemic phase into the endemic phase. Endemicity refers to the pathogen being maintained at a stable and low prevalence rate, such as influenza [55]. The endemic phase means that the transmission of the virus is under control, unlike the pandemic phase, which has a widespread and uncontrolled transmission. If one of the SARS-CoV-2 variants mutates, increasing the immune escape property and infectivity, a new pandemic phase for COVID-19 could occur. Close monitoring of new variants of SARS-CoV-2 from public health organizations and governments is still needed to reduce the recurrence chance of the pandemic.

## 3. Pathogenesis

SARS-CoV-2 is made of four structural proteins. They are the spike, envelope, membrane, and nucleocapsid proteins [56]. The spike protein has two functional domains, called S1 and S2 [57]. The S1 domain facilitates receptor binding, while the S2 domain facilitates viral membrane fusion with the cell [58]. The S1 domain is further classified into the N-terminal domain (NTD) and receptor-binding domain (RBD). The RBD is the most variable part and is the part that interacts with cell receptors [59]. Based on the fact that SARS-CoV-2 and SARS-CoV have high genetic similarity [60], scientists identified that angiotensin-converting enzyme 2 (ACE2) receptors are used by SARS-CoV-2 to enter host cells. Transmembrane serine protease 2 (TMPRSS2) is crucial for the proteolytic processing of the S protein, which is important for membrane fusion and entry [61]. ACE2 is abundant in the intestine, lungs, kidney, and heart [62]. When the spike protein of SARS-CoV-2 binds to the ACE2 receptor, the virus starts to infect the cell, and various symptoms may be developed based on the location of the ACE2 receptor that the virus binds to [63]. For example, lung inflammation may happen when SARS-CoV-2 binds to the ACE2 receptor located in the lungs [64]. Similarly, myocarditis may happen when SARS-CoV-2 binds to the ACE2 receptor located in the heart [65].

The innate immune response to SARS-CoV-2 is inflammation. When SARS-CoV-2 enters the cells, it triggers the pattern recognition receptor (PRR) to detect the pathogen-associated molecular patterns (PAMPs), which are responsible for stimulating an innate antiviral response [66]. The host immune cells then produce proinflammatory cytokines (IL-6) and chemokines (CCL2) against the virus [67]. Type 1 interferons (IFN-1) are also released upon the recognition of PAMPs by PRR [68]. IFNs are responsible for antiviral reactions [69]. IFN-1 can trigger the release of enzymes that can degrade viral nucleic acids or produce proteins that block the release of viral particles from infected cells [69]. However, SARS-CoV-2 can counteract the innate immune response from IFN-1 by inhibiting the synthesis of IFN-1. Viral nonstructural proteins from SARS-CoV-2 can block the signaling pathway for IFN-1 synthesis and degrade the mRNA coding for IFN-1 [70].

If SARS-CoV-2 causes the release of excess proinflammatory cytokines, such as IL-6, a cytokine storm occurs. Cytokine storm is considered a poor prognostic marker, as it is commonly found in critically ill COVID-19 patients [71]. The interaction between the viral spike protein and the ACE2 receptor on macrophages can lead to cytokine storms [72]. Consequently, tissue damage in different organ systems (heart, liver, and kidney) and organ failure may happen due to the cytokine storm [73].

Adaptive immune responses to SARS-CoV-2 include the production of antibodies and the killing activity of CD8+ lymphocytes. Besides, CD4+ lymphocytes can coordinate the humoral immune response against SARS-CoV-2 [74]. This leads to the production of IgM, IgG, and IgA antibodies for SARS-CoV-2 [74]. Neutralizing antibodies against SARS-CoV-2 can bind to viral spike protein or RBD and prevent the virus from infecting host cells [75]. Nevertheless, antibodies also contribute to antibody-dependent cell-mediated cytotoxic effects and cellular phagocytosis [76].

### 3.1. Infection Stage of SARS-CoV-2

The infection of SARS-CoV-2 can be split into three stages. The first stage is the incubation period, where the person is infected by SARS-CoV-2. At this stage, viruses start replicating and symptoms are usually not developed. The second stage is the dissemination stage, in which acute pneumonia along with other symptoms, such as cough and fever, may develop in the patients [77]. Some patients who have diabetes or cardiovascular disease may experience more severe symptoms, such as hypoxia [78]. If the patient does not recover from the second stage, stage three, which is the most severe stage, will happen. Patients in stage three may have systemic hyperinflammation, including kidney injury, hepatocellular injury, and gastrointestinal injury [79,80].

One of the major causes of death among patients with COVID-19 is comorbidity. Comorbidity means having a medical condition in addition to the primary diagnosis of disease in a patient. Common comorbidities include hypertension, diabetes, obesity, cardiovascular diseases, and kidney diseases. Comorbidity is a significant risk factor that can increase the chance of mortality [81]. As comorbidities upregulate the expression of ACE2, the chance of getting COVID-19 and the severity of the disease in individuals with comorbidities would increase correspondingly [81]. Moreover, some patients have more than one comorbidity, which is multimorbidity. It was found that the mortality rate for patients with multimorbidity is doubled compared to those without multimorbidity [82].

### 3.2. Long COVID

#### 3.2.1. Definition of Long COVID

For some COVID-19 patients, they are unable to fully recover and suffer persistent symptoms [83]. In this sense, they suffer from long COVID. Long COVID refers to the persistent symptoms that occur after the initial acute phase of COVID-19. No clear definition for long COVID has been established. The United Kingdom classified long COVID as having persistent symptoms after 12 weeks of the initial onset of symptoms and referred to it as chronic COVID [84]. Another classification is after three weeks of the initial onset of symptoms and is referred to as post-acute COVID [85]. Both post-acute COVID and chronic COVID belong to long COVID. 

#### 3.2.2. Epidemiology of Long COVID

The diagnosis of long COVID is difficult. It cannot be dependent on each individual’s viral status, as most of the long COVID patients have negative results on PCR diagnosis [86]. Different individuals infected with SARS-CoV-2 have different recovery times based on the severity of the illness and their immune systems. One reason is that some of the individuals who were infected by SARS-CoV-2 did not develop any symptoms. The diagnosis of long COVID in asymptomatic individuals without any COVID-19 diagnostic testing is relatively difficult [87]. The occurrence of long COVID is estimated to be about 10 to 30% for non-hospitalized patients and 50 to 70% for hospitalized patients [88]. Only 10 to 12% of the vaccinated patients were diagnosed with long COVID [89].

The symptoms of long COVID differ for every individual and can affect different organ systems, including respiratory tracts, gastrointestinal tracts, joints, central and peripheral nervous systems, bone marrow, and the endocrine system [90]. Most general symptoms include headache, depression, and fatigue [91]. Myalgic encephalomyelitis/chronic fatigue syndrome (ME/CFS) is another syndrome that has similar symptoms to long COVID, such as fatigue and post-exertional malaise [92].

#### 3.2.3. Pathogenesis of Long COVID

One potential explanation for fatigue and hypoxia in tissues is that SARS-CoV-2 induces the platelets and endothelial cells for hypercoagulation [93]. This blocks the blood vessels and affects oxygen delivery to the tissues. The spike protein from SARS-CoV-2 can increase inflammatory signaling by activating platelets [94]. It can also increase fibrinogen aggregation, which forms micro-clots [95]. If the fibrinolysis for the micro-clots is unsuccessful, hypoxia occurs.

Another possible reason for long COVID symptoms is the disruption of mitochondrial function. Mitochondrial dysfunction in peripheral blood mononuclear cells in COVID-19 patients correlates with the severity of the disease [96]. Due to mitochondrial dysfunction, cells may not generate sufficient ATP to carry out their normal function. It causes the normal cellular energy metabolism to be altered and potentially causes a wide variety of symptoms of long COVID.

For neurological symptoms caused by long COVID, such as anosmia, magnetic resonance imaging has shown damages in the olfactory bulb and mucosa [97]. Acute COVID-19 can cause irreversible damage to olfactory stem cells, as they express the ACE2 receptors [98]. The loss of the olfactory stem cells negatively affects the repairing of the olfactory epithelium [99]. Persistent anosmia remains even after recovery from COVID-19, as the loss of olfactory stem cells renders olfactory repairing ineffective.

#### 3.2.4. Treatments for Long COVID

Treatments for long COVID are different among patients, as each of them may have different symptoms. Clinical assessment should be conducted carefully to detect whether the individual has developed long COVID. Treatment should be in different directions, such as providing symptomatic treatment, psychological support, occupational therapy, and physiotherapy [85]. One approach is to provide treatment based on the identified symptoms and interfere with the related mechanisms [100].

The most effective way to prevent long COVID is by vaccination. A study conducted in the United Kingdom, Spain, and Estonia found that vaccination consistently reduced the risks of developing long COVID symptoms, particularly for adults [101]. Another study found that vaccination before infection with SARS-CoV-2 is associated with a lower incidence of long COVID [102]. If the patients have ongoing long COVID, vaccination cannot assist in resolving the symptoms of long COVID [102]. Therefore, vaccination is a prevention method for reducing the probabilities of developing long COVID and should be conducted as soon as possible.

Metformin is a drug used for the treatment of diabetes. It was found that metformin can be used in patients with COVID-19 and diabetes to lower the risk of developing long COVID [103]. Moreover, in July of 2023, two RECOVER clinical trials were held by the US National Institutes of Health to test the effectiveness of the treatment for long COVID [104]. One of the RECOVER clinical trials was called RECOVER-VITAL, and it aimed to use Paxlovid as a potential treatment for long COVID [104]. Another RECOVER clinical trial was called RECOVER-NEURO [105]. It aimed to use three different treatment methods to help long COVID patients with neurological symptoms. It consisted of two online training programs and one non-invasive electrical stimulation program for the brain [105]. As of June 2024, two more RECOVER clinical trials are launching. They are RECOVER-SLEEP and RECOVER-AUTONOMIC [106,107]. RECOVER-SLEEP aims to test the efficacy of modafinil and solriamfetol for the treatment of hypersomnia. It also aims to test the efficacy of melatonin and light therapy for treating complex sleep disturbances [107]. RECOVER-AUTONOMIC aims to test the efficacy of Gamunex-C and Ivabradine against an autonomic nervous system disorder, called Postural Orthostatic Tachycardia Syndrome [106]. Through all these clinical trials, more potential treatment options are opened to patients suffering from long COVID.

The chronic symptoms for patients with long COVID add another burden to the healthcare system. If people are unwilling to get vaccinated in response to the new variants of SARS-CoV-2, more people will be infected and hospitalized by COVID-19. Those people can also develop long COVID after recovery from COVID-19, which adds extra burdens to the healthcare system. People should get vaccinated with the latest booster dose to keep up with the constant mutating variants of SARS-CoV-2 to remove some burden on the healthcare system.

## 4. Diagnostic Methods

Considering the challenges that COVID-19 can spread in the community through asymptomatic and pre-symptomatic transmissions [108,109], respiratory droplets, and direct and indirect contacts, as the primary means for the transmission between individuals [110], it is possible for coronavirus carriers to unknowingly infect nearby people. To prevent further transmission of disease by unaware carriers and suspected COVID-19 patients, reliable diagnostic techniques are required for early patient identification and better infection control. As such, there was a rapid bloom of research starting from the early stage of the COVID-19 pandemic, which aimed to provide insights into the diagnostic technologies for SARS-CoV-2 infection. However, not all the suggested techniques have been applied in clinical settings or generally accepted for diagnosis. Now, there are two primary categories of diagnostic techniques for SARS-CoV-2 infection recommended by the WHO and FDA. These are nucleic acid amplification tests (NAAT) and serological tests for viral antigens [111,112]. In the following, relatively popular techniques within these two diagnostic categories will be discussed. The characteristics of some featured diagnostic techniques are summarized and shown in Table 2.

### 4.1. Nucleic Acid Amplification Test (NAAT)

#### 4.1.1. RT-qPCR

RT-qPCR is the most stand-out technique used for detecting SARS-CoV-2 in the category of NAATs [25]. It is the most used method and is considered the gold standard for detecting SARS-CoV-2 by the World Health Organization (WHO) [113,114]. One of the reasons contributing to the prevalence of this method is its high sensitivity and specificity [113]. The result generation time of RT-qPCR can vary due to different primers and fluorescence probe designs. As reported previously [115], with the refinement in primers’ design to make their Tm value closer to the optimal elongation temperature of Taq polymerase and shortening the distance between the fluorophore and quencher in the fluorescent probes, scientists could reduce the time required for RT-qPCR SARS-CoV-2 detection from 74 min to 26 min [116].

Several types of patient samples are acceptable for RT-qPCR, which include posterior oropharyngeal saliva, nasopharyngeal swabs, oropharyngeal swabs, and sputum [113]. The nasopharyngeal swab is regarded as the gold-standard sample for SARS-CoV-2 detection [117], probably due to its wide application in the detection of other respiratory pathogens and ease of acquisition [117,118]. As such, it is more prevalent to use nasopharyngeal swabs over other upper respiratory tract samples for the diagnosis of COVID-19 using RT-qPCR. Lower respiratory tract samples, such as sputum, can be an alternative specimen for the diagnosis of COVID-19. Some studies have proved that RT-qPCR using sputum has a higher clinical sensitivity compared to nasopharyngeal swabs [11,56]. However, there is a problem associated with sputum sampling. As stated by Akowuah et al. [119], not all COVID-19 patients produce sputum spontaneously. Although sputum induction can be applied to these patients, it requires a longer sampling time and extra medical staff to look after patients compared to nasopharyngeal swabs. Considering the heavy workload of medical staff over the course of the COVID-19 pandemic, induced sputum sampling may be less preferred or impractical. The difficulty in sampling might have limited sputum’s prevalence for SARS-CoV-2 detection via RT-qPCR. Yet, scientists suggested that sputum can be used for performing a diagnosis of those with a wet cough [120], radiologically or epidemiologically suspected COVID-19, but with a negative nasopharyngeal swab result [121]. 

**Figure 5:**
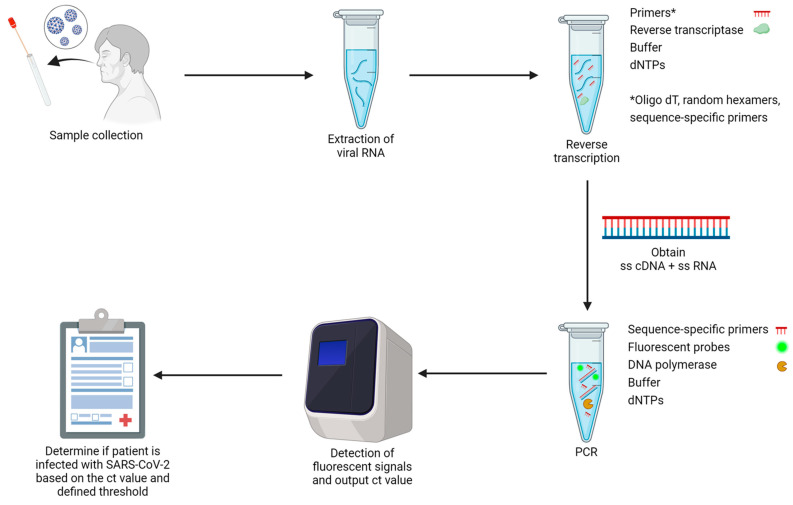
General workflow for a two-step RT-qPCR for the detection of SARS-CoV-2 virus. This figure was created with BioRender (https://biorender.com; accessed on 11 June 2024).

The detection of SARS-CoV-2 via RT-qPCR encompasses three major steps, which are reverse transcription, polymerase chain reaction, and fluorescence detection. The general workflow and setup of an RT-qPCR are depicted in Figure 5. As a single-stranded RNA virus [122], the genomic content of SARS-CoV-2 cannot be directly amplified through PCR. As such, a reverse transcription step is added before amplification. This is achieved by using an RNA-dependent DNA polymerase, which converts the single-stranded RNA into complementary DNA [123]. In terms of DNA amplification, scientists spotted conserved sites in the genome of SARS-CoV-2 to produce primers that are usable for more virus variants. Three conserved sites were found in the SARS-CoV-2 genome, which are the RdRP (RNA-dependent RNA polymerase) gene, the E (envelope protein) gene, and the N (nucleocapsid protein) gene [123]. Depending on the region, different combinations of genes are used for the detection of SARS-CoV-2. Except for the three genes mentioned above, there are also S (spike protein), *ORF1a*, and *ORF1b* genes, which may be targeted for DNA amplification [114,123]. With the genes of SARS-CoV-2 being successfully probed and amplified, PCR products will be labeled with fluorescent dyes or probes during thermal cycling and the qPCR machine will measure the changes in fluorescence intensity over time. The number of threshold cycles (Ct) aids in identifying whether a patient is infected with SARS-CoV-2 and his or her viral load [124]. However, the Ct value can vary due to several reasons, including the genes and platforms used for detection, the source of the specimen, and its degradation [113]. As such, there is no universal guideline for interpreting the Ct values obtained from RT-qPCR of all specimen types. Countries or regions may define their own set of Ct values by considering the gene panels used and the specimen types to determine the infectiousness of the tested individual. They may also consider whether follow-up tests and infection control actions are required based on the result. From a general perspective, for nasopharyngeal samples, Ct values that fall into the range of <20, 20–25, <30, and 30–33 signify a very high viral load, high viral load, moderate to low viral load, and very low transmitting risk, respectively [113].

However, for such a popular and high-credibility SARS-CoV-2 detection method, it possesses shortcomings, too. One of the risks with the use of RT-qPCR is a higher chance of obtaining false-positive and false-negative results. In terms of false-positive results, they may be obtained if there are problems such as suboptimal handling and contamination, even at a small amount. For instance, trace amounts of nucleic acids retained from previous tests in the PCR machine may be amplified and lead to false-positive results [113]. For false-negative results, their root causes can be poor collection, handling, and transit [123]. Ordering an RT-qPCR test at inappropriate time points may also yield false-negative results. Ideally, the test provides reliable results three days after the manifestation of symptoms and until the fourteenth day [125]. The reason is that the low viral loads at the beginning of COVID-19 infection and in the recovery stage are potential causes of false-negative results. As such, RT-qPCR has a high requirement on the experience and techniques of laboratory personnel [123], as well as a clean and well-planned working environment that can ensure a low contamination rate for the samples [126]. Clinicians should also order the RT-qPCR test after considering the patient’s exposure time to the virus.

Besides, RT-qPCR urges for rigorous primer design for good performance. As the major criteria for assessing assay performance, sensitivity and specificity of RT-qPCR depend on primer quality. A previous study demonstrated that the use of N-gene-targeting in-house-designed primer sets (iNP) for SARS-CoV-2 detection had a higher sensitivity than the N1-, N2-, or N3-gene-targeting primer sets recommended by the Centers for Disease Control and Prevention (CDC) when the cycle threshold was set as 35 (i.e., 94.8% vs. 57.9–63.2% for sputum samples and 69.6% vs. 65.2–69.6% for nasopharyngeal samples) [127]. While the differences between the iNP and CDC primer sets were primer sequences and the amplification temperatures differed by 2 °C, it was likely that the difference in target regions on the N gene contributed greatly to the variance in analytical sensitivity between the iNP- and CDC-suggested primers. Besides, cross-reactivity with other respiratory pathogens, such as influenza A virus, SARS-CoV, and *Staphylococcus aureus*, was found with the use of CDC-recommended primer sets [127], while the iNP primer set did not have false reactive results on the cross-reactivity panel. It is possible that the imprecise design of the CDC primer sets resulted in the detection of undesired targets. 

Aside from the primer design, specimen type may affect the clinical sensitivity of RT-qPCR assays. As observed by Lawrence Panchali et al. [127], using iNP for detecting SARS-CoV-2 in sputum could achieve 94.8% sensitivity; however, sensitivity was greatly reduced when nasopharyngeal swabs were used (i.e., 69.6%). This may be explained by the detection principle of RT-qPCR and viral distribution in the respiratory tract. RT-qPCR detects viral genes, and the amount of these genes is proportional to viral abundance. If a sample contains a scarce amount of virus, the viral gene copies may be below the limit of detection and, hence, cannot be reliably detected, leading to false negatives. As Bello-Lemus et al. commented [128], the detection sensitivity of viral genes ‘decreases with the decrease in the patients’ viral load’. Regarding viral distribution, several studies have reported that the viral load in the lower respiratory tract samples (i.e., sputum) is generally higher compared to upper respiratory tract samples (i.e., nasopharyngeal swabs) at the beginning of the disease [129,130]. This difference in viral distribution may be attributed to a higher density of angiotensin-converting enzyme-2 (ACE2) receptors and less exposure to the external environment in the lower respiratory tract [130]. The high RNA viral load in the lower respiratory tract can be easier to detect, leading to a higher clinical sensitivity for SARS-CoV-2 detection when using sputum samples. Furthermore, some studies have suggested that the viral shedding time may be longer in the lower respiratory tract than the upper respiratory tract [118,130,131]. This prolonged viral shedding in the lower respiratory tract could result in a persistent positive result for SARS-CoV-2 using sputum samples, even though nasopharyngeal swabs have turned negative [118,130]. 

All things considered, there are various measures to ensure high-quality results for RT-qPCR results. Every medical practitioner involved in sampling, processing, and storing of patient samples for RT-qPCR should strictly adhere to established guidelines to avoid faulty results. Besides, the diagnostic kit in use should be regularly evaluated by laboratories. For instance, literature reviews of recent studies on analytical performance can show whether the kit is capable of detecting important and up-to-date SARS-CoV-2 variants. Moreover, the reliability of results may be validated through external quality-assurance programs. Using more than one brand of diagnostic kits for result cross-checking can also ensure the results’ reliability. Regarding variation in clinical sensitivity across sample types, it may be necessary to standardize the sample used for virus detection, or different guidelines for result interpretation can be established for different specimen types.

#### 4.1.2. Multiplex RT-qPCR Assays for Detecting SARS-CoV-2 and Other Respiratory Pathogens

The invention of multiplex RT-qPCR respiratory panels was a remarkable technological advancement during the COVID-19 pandemic. Since the start of the pandemic, over 200 RT-qPCR commercial diagnostic kits have been granted emergency use authorization (EUA) from the FDA. However, only a small proportion of them were designed as multiplex assays, which allow for simultaneous detection of SARS-CoV-2 and other respiratory pathogens. To date, three out of seven RT-PCR diagnostic kits are multiplex assays and had their EUA issued and updated this year [132]. They are Cobas liat SARS-CoV-2, Influenza A/B and RSV nucleic acid test, ePlex Respiratory Pathogen Panel 2, and PKamp Respiratory SARS-CoV-2 RT-PCR Panel 1. While there is a lack of studies evaluating the performance of the newly approved Cobas liat kit (received FDA-EUA approval on 7 June 2024), the latter two assays were reported to have 100% sensitivity and specificity for SARS-CoV-2 detection [133]. Besides, these two assays were found to have 100% concordant results with the CDC 2019-nCoV RT-PCR diagnostic panel [134], and slightly higher detection rates for SARS-CoV-2 in nasopharyngeal swabs and saliva samples compared to another SARS-specific FDA EUA assays [135], respectively. These multiplex assays serve an important role in assisting clinicians in making accurate diagnoses and providing appropriate treatment to patients. As reported previously [136], co-infection with other respiratory pathogens was discovered in some COVID-19 patients. Moreover, the application of multiplex assays enables efficient testing, since fewer runs and machines are required for detecting and differentiating multiple pathogens in a sample.

#### 4.1.3. RT-dPCR

During the COVID-19 pandemic, consecutive negative results from RT-qPCR testing have been one of the considerations for hospital discharge or the end of quarantine for infected individuals [118,137]. Ensuring the accuracy of RT-qPCR results was particularly important during that period, as faulty results could have led to the wasteful use of medical resources on recovered individuals, and the potential exacerbation of the pandemic due to the continued spread of infectious virus particles from patients who had not fully recovered. Acknowledging the shortcomings of RT-qPCR, scientists suggested using RT-dPCR as an alternative to RT-qPCR because of its improved sensitivity and accuracy [138].

To apply dPCR in the detection of SARS-CoV-2, a reverse transcription step is required to produce cDNA from the viral RNA. With the cDNA successfully generated, a PCR mixture has to be prepared, of which the ingredients are similar to that of qPCR. The PCR mixture is then partitioned into thousands of droplets, each containing none or one to multiple nucleic acid molecules. Each droplet is a PCR reaction, in which nucleic acids will be amplified in the same way as qPCR. Following the amplification cycle, the PCR machine will detect the presence of target nucleic acids with the help of fluorescence probes. The number of droplets with fluorescence signals will be counted, and then the concentration of target nucleic acid molecules will be determined using Poisson statistics. 

Compared to qPCR, dPCR was found to have a higher sensitivity and tolerance to interference. In terms of analytical sensitivity, a previous study affirmed that the limit of detection (LoD) of droplet dPCR is 1.8 copies/reaction and 2.1 copies/reaction for the N gene and *ORF1ab* gene, respectively [139]. Meanwhile, for qPCR, the LoD for the N gene and *ORF1ab* gene is 873.2 copies/reaction and 1039 copies/reaction, respectively. Based on these findings, droplet dPCR was 500 times more sensitive compared to qPCR. Dong et al. [138] also affirmed the high clinical sensitivity of dPCR with their experimental data. In their study, 61 previously negative or equivocal samples tested with RT-qPCR were later confirmed to be positive with RT-dPCR. Through clinical symptoms, resampling, and retesting with RT-qPCR, these 61 patients were later diagnosed with COVID-19. For these 61 patient samples, the average viral loads were 25, 26, and 31 copies/reaction for the N, E, and *ORF1ab* genes, respectively. Meanwhile, high viral loads were observed in another 29 positive samples tested by RT-qPCR. The average viral loads for these 29 samples were 1099, 2594, and 998 copies/reaction for the N, E, and *ORF1ab* genes, respectively. These results suggested the potential application of dPCR in detecting SARS-CoV-2 at a low abundance. Except for its high sensitivity, dPCR was also expected to have a higher tolerance to PCR inhibition due to the partitioning of the reaction mixture [138]. 

While dPCR seemed to be a promising technique for combating the COVID-19 pandemic, there were some concerns with its widespread use. For partitioning a PCR mixture, specialized machines and dPCR chips are required, which could be a financial burden for poorer or resource-limited laboratories. Also, the production of dPCR chips is time-consuming [140]. At the peak of COVID-19 testing demands, it was challenging for manufacturers to produce and distribute sufficient dPCR chips to laboratories worldwide. In addition, the WHO later removed the requirement of receiving consecutive negative RT-qPCR results for patient discharge from its guidelines [141]. The reason was that for patients with symptoms manifesting for more than eight weeks, their nasopharyngeal swabs may still give out positive results due to prolonged viral shedding, while the median recovery time is only about 14–15 days after symptoms appear [141]. Since then, the demand for a highly sensitive yet more expensive NAAT, such as dPCR, was reduced, as its results were no longer critical for making infection control decisions.

#### 4.1.4. Other NAATs

Aside from RT-qPCR, in the mid-phase of the COVID-19 pandemic, there was the suggestion of using isothermal nucleic acid simplification technologies, such as LAMP (loop-mediated isothermal amplification) [142] and CRISPR-based assays [143]. LAMP is the most well-established isothermal amplification technique [114]. Compared to RT-qPCR, RT-LAMP does not require thermal cycling and the result generation time ranges from 5 to 13 min [123]. As such, less complicated machines were needed, and a shorter turn-around time could be obtained through applying RT-LAMP in SARS-CoV-2 detection. However, possibly due to the difficulty in designing a set of four to six robust primers for LAMP and the good establishment of RT-qPCR, RT-qPCR has dominated for SARS-CoV-2 detection in the field of NAATs [144]. Since the beginning of the COVID-19 pandemic, the FDA has granted EUA approval to only 17 RT-LAMP test kits, which is much fewer than that of RT-PCR (i.e., more than 200 entries) [132].

### 4.2. Serological Tests

Serological tests can be divided into antibody-based or antigen-based [114]. Respectively, they detect the presence of virus-specific host antibodies and viral antigens in tested individuals. However, antibody-based serological tests are not recommended for the diagnosis of acute SARS-CoV-2 infection [145]. Instead, its major function is to suggest whether an individual has been infected or recovered from SARS-CoV-2 previously [146]. The reason why antibodies cannot be used for diagnosis is that patients may take 1–2 weeks to give out a measurable amount of SARS-CoV-2 antibody after the onset of illness [145]. Therefore, antibodies cannot provide an immediate snapshot of the viral abundance in tested individuals. Adversely, antigen-based tests that detect particular SARS-CoV-2 antigens have a high diagnostic value. This is because the presence of antigens signifies current viral infection [147].

The detection of SARS-CoV-2 antigen can be performed by rapid antigen test (RAT) kits or ELISA (enzyme-linked immunosorbent assay). These two techniques also employ capture antibodies targeting spike or nucleocapsid proteins, which can be regarded as the antigens of SARS-CoV-2. However, the epitopes targeted by these capture antibodies may vary between brands and products. This may give rise to variance in the performance of antigen-based tests regarding coverage and sensitivity. Similar to RT-qPCR, antigen-based tests are compatible with nasopharyngeal swabs, oropharyngeal swabs, or sputum samples (depending on the design of the test) [113].

#### 4.2.1. RAT

**Figure 6:**
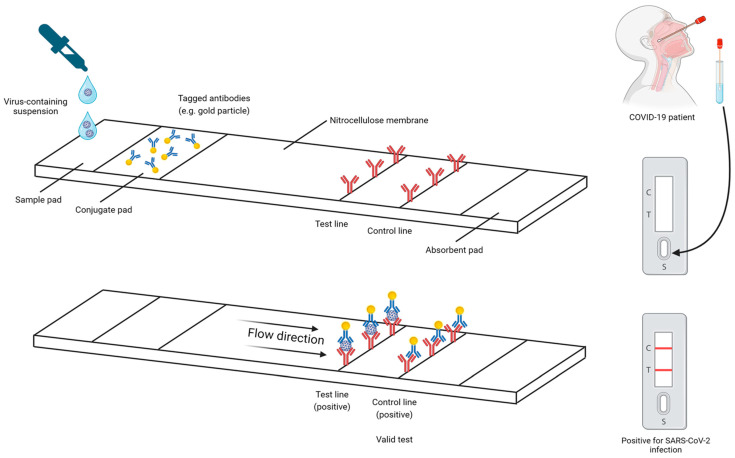
The architecture of LFIA for SARS-CoV-2 virus detection. This figure was created with BioRender (https://biorender.com; accessed on 11 June 2024).

Most of the SARS-CoV-2 RATs are designed based on the principles of a lateral flow immunoassay (LFIA), of which the design is shown in Figure 6. In LFIA, a specimen is first immersed into a buffer, which may help with the flow of viral particles along the nitrocellulose membrane in a RAT kit. After that, droplets of analyte are added to specific sites on the test kit. On the nitrocellulose membrane, there are usually three types of antibodies: antibodies specific for SARS-CoV-2 viral proteins and tagged with substances such as gold, latex, or fluorophore [148], antibodies immobilized on the test line and specific to SARS-CoV-2 viral proteins, and antibodies immobilized on the control line and specific to part of the tagged antibodies. Analyte flows through the nitrocellulose membrane by capillary action and antigen–antibody complexes will be formed once there is complementary binding between antigens and antibodies. Ultimately, a positive result will be visualized as a colored or fluorescence band depending on the tag linked to antibodies, and vice versa for a negative result. The control line should always show a positive result [149], as it signifies successful binding between immobilized antibodies and excess tagged antibodies. Otherwise, there may be problems, such as an insufficient sample volume, which makes tagged antibodies unable to reach the control line or components in the test kits degraded. The test should be repeated in this case. There is also another scenario that may require repeat testing. As recommended by the FDA, users who obtain a negative result using FDA-approved over-the-counter diagnostic tests should repeat testing to lower the risk of receiving a false-negative result [150].

The invention of RATs has significantly promoted self-testing during the COVID-19 pandemic, as they do not urge for expensive reagents, laboratory equipment, multiple processing steps, and trained staff for manipulation. These characteristics of RATs have contributed to their lower cost compared to laboratory-based techniques, such as RT-qPCR. Furthermore, a recent meta-analysis study revealed that self-testing and professional testing of SARS-CoV-2 infection using RATs could achieve similar accuracy, with a pooled Cohen’s kappa of 0.91 [151]. Additionally, RATs can yield results in 10–15 min [123], which is shorter than RT-qPCR, which needs one or more hours to obtain the results. In short, the cost effectiveness, ease of manipulation, and short result generation time of RATs have made them one of the invaluable tools for combating the COVID-19 pandemic.

#### 4.2.2. ELISA

In terms of ELISA, its derivative, sandwich ELISA, has been reported to be used in the detection of SARS-CoV-2 viral proteins [113,152]. Compared to RATs, the development of ELISA-based SARS-CoV-2 antigen tests came later and was less common. This may be due to the reasons that ELISA requires a longer result generation time and is not suitable for self-testing since it is a laboratory-based technique. Despite these limitations, ELISA is generally more sensitive than LFIA and has a lower production cost per sample [152]. The use of ELISA may help to reduce the financial burden on public health systems. Recognizing these advantages, some scientists have worked on the development of ELISA for the detection of SARS-CoV-2 antigen [152,153]. These ELISA kits may have greater importance in a clinical setting, where numerous COVID-19 samples are available. While most of the SARS-CoV-2 antigen diagnostic tests are LFIA at present, it is possible that more ELISA-based detection kits could emerge in the market in the future. This is because the WHO has stated that platforms other than RAT will be considered on a case-by-case basis for granting emergency use listing (EUL) approval [111]. 

**Figure 7:**
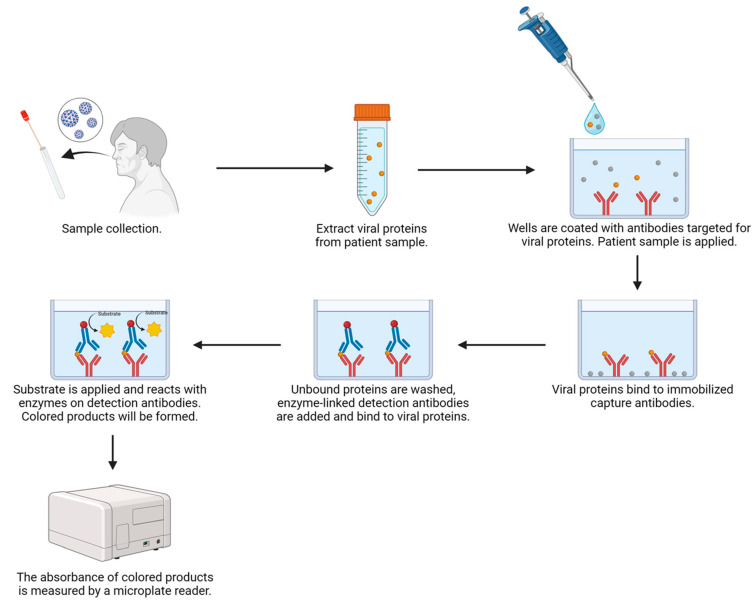
General flow for using ELISA in SARS-CoV-2 viral antigen detection. This figure was created with BioRender (https://biorender.com; accessed on 11 June 2024).

The general procedure of an ELISA-based SARS-CoV-2 antigen test is shown in Figure 7. To begin with, capture antibodies specific to SARS-CoV-2 viral proteins are first immobilized on a solid support. Following this, a lysis buffer is used to release viral particles from the specimens. If the SARS-CoV-2 virus possesses antigens that can specifically bind to the capture antibodies, antigen–antibody complexes will be formed. For the detection of those antigen–antibody complexes, enzyme-linked or fluorescently labeled detection antibodies with or without primary detection antibodies will be used. Additional substrates have to be added in case of enzyme-linked detection antibodies so that chromogenic products can be formed and quantified by a detector. Eventually, a detector, such as a microplate reader, can be used to measure the intensity of fluorescence, or the color intensity of the chromogenic substances formed. The value measured is proportional to the number of antigen–antibody complexes inside a well of the microplate. Users may construct a standard curve for the semi-quantitative measurement of the SARS-CoV-2 virus in patient samples.

As commented by the Pan American Health Organization and the WHO, ELISA for detecting SARS-CoV-2 viral proteins has acceptable specificity and could be set as one of the confirmation criteria, together with clinical and epidemiological histories and case definitions. ELISA results were also useful for making public health decisions [113].

#### 4.2.3. Shortcomings of Antigen Tests

However, antigen tests are not as reliable as NAATs due to a lower sensitivity [147,151]. There are two possible reasons for the difference in performance. Firstly, antigen-based serological tests lack an amplification step. To achieve a high confidence in SARS-CoV-2 detection, the tested individual should have a moderate to high viral load. Particularly, for RAT, its results are most reliable when the patient has a virus copy number higher than 10^6^ per mL [113]. Such a great viral load usually occurs in the pre-symptomatic state, which is one to three days before the onset of symptoms, and the early symptomatic state [154]. While the mean latent period for SARS-CoV-2 is about 5.5 days [155], it is possible for an antigen-based test to yield a false-negative result when the tested individual is only infected by the SARS-CoV-2 virus for a short period. Probably, a low abundance of SARS-CoV-2 antigens present in the specimen would give rise to inadequate numbers of antigen–antibody complexes, which together cannot produce a measurable or observable amount of signal. While the SARS-CoV-2 antigens cannot be amplified, as nucleic acid does in RT-qPCR, patients with a low SARS-CoV-2 load may be omitted by the antigen-based serological tests, giving rise to false-negative results.

Another reason is that mutations in the spike or nucleocapsid protein of SARS-CoV-2 may lead to detection failure in antigen-based tests. In recent years, SARS-CoV-2 has evolved and mutated, yielding numerous variants [156]. In these virus variants, their viral proteins may be altered. Although most of the single-nucleotide polymorphisms (SNP) that define the variants of SARS-CoV-2 occur in the spike protein [157], mutations in the nucleocapsid protein of SARS-CoV-2 have also been reported [158]. As such, antigen-based tests may not be able to detect particular SARS-CoV-2 variants with their spike or nucleocapsid proteins mutated. Comparatively, most of the RT-qPCR platforms target conserved gene sequences in SARS-CoV-2, hence they have a lower chance to overlook those newly emerged SARS-CoV-2 variants. With that being said, companies may perform constant regulatory reviews of their RAT products to improve the design of capture antibodies. This may help to improve the coverage and sensitivity of RATs. However, this may not be a usual practice for companies due to financial burdens. As such, before making the diagnosis of SARS-CoV-2 infection, healthcare providers should have first gone through the packing inserts of the RAT products to understand what SARS-CoV-2 variants can be detected. It is also advised to use RATs that are granted EUA from the FDA. As of May 2022, among 577 commercialized SARS-CoV-2 RATs recorded on the FIND (http://www.finddx.org, accessed on 15 April 2024) website, only 38 of them have been granted emergency use authorization from the FDA [113]. The use of unauthorized antigen-testing kits may produce inaccurate results and hence affect diagnosis. If necessary, RT-qPCR may be used for making the diagnosis.

## 5. Ancillary Tests for COVID-19

In addition to diagnostic tests, as mentioned above, several other techniques have been employed for monitoring the mutational dynamics of SARS-CoV-2 and patients’ health during the pandemic. Some important techniques will be reviewed below.

### 5.1. Sequencing

Although sequencing has limited efficacy for rapid virus screening of the general public due to the long turnaround time, high cost, and the requirement of expertise for data processing and results’ interpretation [159], sequencing data have made important contributions in the fight against the COVID-19 pandemic. 

Firstly, sequencing data have facilitated the understanding of SARS-CoV-2 biology. The original genome sequence of SARS-CoV-2 was obtained with metagenomic RNA sequencing. It was then promptly disclosed to the public and uploaded to the GenBank sequence database in January 2020 [160]. With this genomic information at hand, scientists were able to gain critical insights into the novel virus in the early stages of the COVID-19 pandemic. They were able to determine the virus’ genus, identify its genetic similarities with other Beta coronaviruses, infer the receptor it uses for cell entry, and recognize an insertion in the S gene that may improve the virus’ infectivity [161]. Besides, comparative genomic analysis provided hints about the potential animal reservoir host of SARS-CoV-2 [161]. This fundamental knowledge acquired from genome sequencing was instrumental for the implementation of preventive measurements targeting the human–animal interface as well as human-to-human transmission.

Secondly, SARS-CoV-2 sequencing data have been of paramount importance in developing various techniques against the COVID-19 pandemic. For molecular diagnostic tests, such as RT-qPCR, the genome sequences of SARS-CoV-2 are essential for the design of specific primers and fluorescent probes that can accurately and reliably detect the presence of the virus’ nucleic acids in patient samples [162]. For serological tests, the virus genome sequence enabled researchers to infer the protein sequences of viral antigens, such as the N and S proteins. Based on this understanding, synthetic viral antigens can be produced by introducing corresponding gene fragments into expression vectors [162,163]. These synthetic viral antigens can then be used in serological assays for the detection of host antibodies against SARS-CoV-2 antigens, and hence contribute to the study of a patient’s immune response and exposure to the virus. Besides, the design of COVID-19 vaccines also relied on the insights gained from SARS-CoV-2 genomic sequencing. Taking mRNA vaccines as an example, the gene fragments encoding the SARS-CoV-2 spike protein are introduced to the muscle cells of recipients [164]. As long as recipients’ muscle cells make copies of the spike protein, an immune response will be elicited. Similarly, for protein subunit vaccines, synthetic spike protein antigens are produced using expression vectors and then administered to recipients [162,164]. Host immune cells will recognize injected viral antigens as ‘foreign’ and initiate an immune response.

Thirdly, sequencing is crucial for SARS-CoV-2 genomic surveillance. As an RNA virus, SARS-CoV-2 has a high tendency of genetic mutation. Consequently, there is the emergence of numerous SARS-CoV-2 variants, including variants of interest (VOIs) and variants of concern (VOCs). These variants, such as the Alpha, Beta, Delta, Omicron, and their subvariants, may gain advantages, such as increased transmissibility or the ability of immune evasion [165]. Hence, it is essential to monitor the prevalence of these clinically important variants within the COVID-19 patient population. To identify the specific virus variant carried by the patient, sequencing is needed. Genomic surveillance is not just about monitoring variant proportion dynamics, it also includes the tracking of SARS-CoV-2 evolution and the detection of mutations within emerging variants, all of which require sequencing as well. Considering the significance of sequencing to genomic surveillance, some scientists have worked on modifying protocols to increase throughput and reduce the costs of sequencing SARS-CoV-2 [166]. To date, genomic surveillance is still ongoing, and the COVID-19 Real-time Learning Network (https://www.idsociety.org/covid-19-real-time-learning-network/diagnostics/covid-19-variant-update, accessed on 28 June 2024) is one of the continually updated online resource centers providing the latest information to the public.

### 5.2. Antibody Serology Tests

As mentioned above, antibody testing alone is not recommended for the diagnosis of acute SARS-CoV-2 infection. However, it has been an approach used by clinicians to check for recent or prior SARS-CoV-2 infection in tested individuals [167]. For a patient infected by SARS-CoV-2, his/her immune cells will recognize the viral particles as ‘foreign’ and initiate an adaptive immune response. This will lead to the production of antibodies, such as IgM and IgG, targeting viral antigens and being detectable by immunoassays. 

From a general perspective, once a patient has been infected by SARS-CoV-2, their IgM level would typically begin to rise within the first week of infection and then peak around two weeks after infection [159]. After that, the IgM level would start to decline slowly [168]. IgG antibodies may be produced simultaneously with or later than IgM [169,170]. The IgG level may peak around 3 weeks after infection, and the high level can persist for up to 48 days [159]. Based on this pattern, IgM antibodies may signify recent SARS-CoV-2 infection, while IgG may indicate previous exposure to the virus. Some studies reported contradictory findings, suggesting that seroconversion of IgG occurred earlier than IgM [169,171,172]. This discordant finding may be attributed to potential cross-reactivity of host antibodies developed against other human coronaviruses, such as HCoV-229E and HCoV-HKU1 [170]. Since the S or N protein of SARS-CoV-2 may have a similar amino acid sequence to those proteins in other members of the Coronaviridae family [169], both antibodies developed against SARS-CoV-2 and antibodies against other coronaviruses may bind to the synthetic SARS-CoV-2 antigen used in immunoassays. Immunoassays may not be able to differentiate between these cross-reactive antibodies, which can lead to faulty experimental results and, consequently, different understandings of the antibody response patterns.

Anti-SARS-CoV-2 IgM or/and IgG antibodies are the popular targets for these immunoassays [173]. Since the commencement of the COVID-19 pandemic, over 70 commercial antibody serological kits have been granted EUA approval from the FDA, and more than half of them detect either IgM or IgG, or both [174].

Various immunoassay-based technologies have been exploited for antibody testing. Some examples are the enzyme-linked immunosorbent assay (ELISA), lateral flow immunoassay (LFIA), and chemiluminescence enzyme immunoassay (CLEIA). While the general principles of ELISA and LFIA are similar to the ones mentioned earlier, there are some differences in their design to specifically detect antibodies. In the case of the indirect ELISA, which is the most common type of ELISA exploited for some FDA EUA antibody serology tests [174], the wells of a microtiter plate are precoated with synthetic SARS-CoV-2 antigens instead of antibodies. The target of enzyme-conjugated detection antibodies changes from viral antigens to human immunoglobulins. For LFIA, antibodies on the conjugate pad are replaced by conjugated viral antigens. The target of immobilized antibodies on the test line becomes a portion of the human immunoglobulins, but not viral antigen [175]. Considering CLEIA, the principle can be similar to indirect ELISA, with a key difference in the detection method. In CLEIA, a chemiluminescence reaction rather than a colorimetric reaction is exploited for the detection of target analytes. There are also related techniques, such as the chemiluminescence immunoassay (CLIA) and chemiluminescence microparticle immunoassay (CMIA). The major differences between these techniques and CLEIA are the exclusion of enzymes from the chemiluminescence reaction, and the coating of synthetic viral antigens on paramagnetic beads instead of wells of a microtiter plate, respectively. Furthermore, chemiluminescence can also be generated through electrochemical reactions, giving rise to another technique, called the electrochemiluminescence immunoassay (ECLIA). Considering the wide range of developed immunoassay techniques, there is a great diversity of COVID-19 antibody testing kits and platforms available in the market at present.

Recently, there were two studies that evaluated the performance of 35 manual serological assays (i.e., 26 rapid diagnostic tests (RDT) and 9 enzyme immunoassays (EIA)) and 25 automated serological assays (i.e., 4 CLEIA, 8 CLIA, 6 CMIA, 2 EIA, 2 ECLIA, and 3 not specified) on the same set of patient samples, with potential cross-reacting and interfering substances [176,177]. The WHO has released Target Product Profiles (TPP), which have listed the acceptable and desirable performance for SARS-CoV-2-related assays. For RDT, the acceptable and desirable levels are ≥90% and ≥95% for sensitivity, and ≥97% and ≥99% for specificity. For higher throughput assays, the acceptable and desirable levels are ≥95% and ≥98% for sensitivity, and ≥97% and ≥99% for specificity. Among the 11 evaluated manual RDTs and EIAs for anti-SARS-CoV-2 IgG only or total antibodies, only 5 and 3 test kits achieved desirable sensitivity and specificity, respectively [176]. Meanwhile, out of 16 automated serology tests for IgG or total antibody against SARS-CoV-2, 8 and 15 test kits met the desirable sensitivity and specificity, respectively [177]. Regarding the cross-reactivity and interference study on serology tests, 12 manual test kits had equal to or less than 3 false reactive results on either the interfering substances or cross-reactive samples panel, and only 1 test kit had no false reactive result on either panel [176]. On the other hand, no automated serology assay had more than three false reactive results on either panel, and eight automated assays did not have any false reactive results [177].

The data above suggest the emergence of a considerable amount of suboptimal manual serology kits in the market during the pandemic. This was likely due to the lack of comprehensive evaluation of the test kit’s performance before allowing its disclosure and distribution to the public [177]. Such a phenomenon also raises a concern that using these substandard test kits could lead to a false interpretation of patients’ health, potentially affecting clinical decisions. While automated assays demonstrated greater adherence to the performance standards, laboratories that perform serology tests should have regular reviews and examinations on the performance of serology test kits in use no matter if they are manual or automated, to ensure the quality of test results. 

## 6. Treatment Strategies

**Figure 8:**
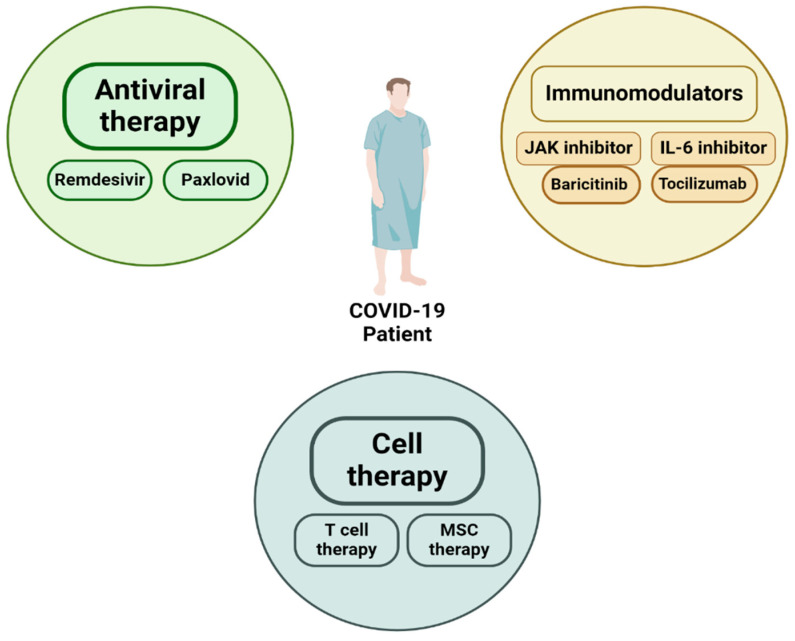
The treatment strategies for COVID-19 patients. This figure was created with BioRender (https://biorender.com; accessed on 6 July 2024).

According to Figure 8, antiviral therapy, immunomodulators, and cell therapy were the potential treatments for COVID-19 in the early outbreak. Most of the treatment strategies were derived from the previous treatments against SARS and MERS, which share similar properties, making those drugs potential candidates for SARS-CoV-2 treatments. Currently, many antiviral and anti-inflammatory agents are well studied in clinical trials, and some of them are approved by the FDA or recommended by COVID-19 guidelines, such as Baricitinib, Paxlovid, and Remdesivir. On the other hand, cell therapy is an emerging strategy for treating COVID-19 and, currently, numerous phase I–II clinical trials are in progress.

### 6.1. Antiviral Therapy

#### 6.1.1. Remdesivir

Remdesivir (GS-5734) is a nucleotide prodrug, which targets the viral RNA-dependent RNA polymerase (RdRp) of SAR-CoV-2. Remdesivir (GS-5734) was synthesized by adding an aryloxy-substituted phosphoryl group and an amino acid ester to its nucleoside GS-441524 [61]. After entering the body, Remdesivir is transformed into its nucleoside GS-441524, Remdesivir triphosphate, which acts as the analogue of adenosine to compete for the binding to RNA chains, resulting in the chain termination [178]. The mechanism of Remdesivir exhibits broad antiviral activity against several RNA viruses, including the Middle East respiratory syndrome (MERs)-related coronavirus, SARS-CoV, and influenza A [179]. The effect on SARS-CoV made Remdesivir a potential drug for treating COVID-19, and the FDA granted an emergency use authorization (EUA) for COVID-19 in 2020. 

Various clinical trials have been performed on the antiviral activity of Remdesivir against SAR-CoV-2 [180]. One randomized, double-blind study (*n* = 562) indicated that Remdesivir (200 mg on day 1 and 100 mg on days 2 and 3) could lower the risk of death or hospitalization by 87% when compared to a placebo [181]. A random clinical trial (*n* = 1062) showed that Remdesivir (200 mg loading dose on day 1, followed by 100 mg daily for up to 9 additional days) had clinical benefits when compared to a placebo or standard care, which shortened the median recovery time from 15 days to 10 days in adults [182]. In addition, several clinical studies suggested that the effect of Remdesivir depends on the viral replication stage, where Remdesivir will be effective in the early stage of infection [181,183]. Conversely, random clinical trials revealed the efficacy of Remdesivir still remains unclear in in-hospital patients. The DisCoVeRy phase III trial on COVID-19 patients (*n* = 857) who were admitted to the hospital indicated that no clinical benefit was observed with the use of Remdesivir in patients who were hospitalized, required oxygen support, and were symptomatic for more than 7 days [184]. The Solidarity random trial on COVID-19 patients (*n* = 14,221) found that the use of Remdesivir had no significant effect on COVID-19 patients who were ventilated, while the effect of Remdesivir on delaying the need for ventilation or death was observed in both clinical trials [184,185]. Moreover, researchers demonstrated that the combination of Remdesivir and baricitinib improved the clinical symptoms and shortened the recovery time [186]. However, a double-blind, randomized phase III trial indicated that the combined use of interferon beta-1a and Remdesivir did not improve the time to recovery in hospitalized COVID-19 patients when compared to the placebo plus Remdesivir group, and even led to a worse outcome in patients who required high-flow oxygen at baseline [187]. 

Due to the massive consumption of Remdesivir during the pandemic, concerns about the possible resistance were raised. Studies found that the mutation in the RNA-dependent RNA polymerase (RdRp) components, non-structural protein 12 (Nsp 12), developed a low level of resistance to Remdesivir [188]. Another study on a phase III adaptive COVID-19 treatment Trial I (ACTT-1) revealed that Remdesivir remains effective against the different Nsp 12 substitutions [189]. In addition, Remdesivir was found to be effective in vitro against the SAR-CoV-2 Omicron variants, XBB.1.9.1 and XBB.1.5, and other variants of concern, which may be due to the highly conserved RdRp among the variants [190,191,192].

More clinical trials have been carried out to solve its shortcomings of low oral availability and stability by developing prodrugs for oral intake, for example, GS-5245 and VV116 [11,183,193]. Recently, a double-blinded, phase III, randomized controlled study on mild-to-moderate COVID-19 patients (*n* = 1369) in China indicated that the use of VV116 significantly reduced the time to sustained clinical symptom resolution in comparison to the placebo group [194]. Another phase III randomized trial found that the use of VV116 was non-inferior to Paxlovid in terms of the time to sustained clinical recovery in the Omicron outbreak [193].

#### 6.1.2. Nirmatrelvir–Ritonavir (Paxlovid)

Nirmatrelvir–ritonavir (Paxlovid) is a combination of Nirmatrelvir, a protease inhibitor, and ritonavir, a cytochrome P450 3A4 (CYP3A4) inhibitor. Paxlovid was granted an EUA in 2021, and the FDA approved it for the treatment of mild-to-moderate COVID-19 in adults that are at high risk of death or hospitalization [195]. Nirmatrelvir is a protease inhibitor that targets the SARS-CoV-2 main protease (Mpro). The SARS-CoV-2 Mpro, together with papain-like protease (PLpro), mediates the cleavage of pp1a and pp1ab polyprotein, leading to the formation of non-structural protein (NSP) 1–16, and hence the formation of RdRp for RNA replication [196]. Previously, nirmatrelvir was used to target the SARS-CoV Mpro. However, researchers revealed that the structure of SARS-CoV-2 Mpro was highly similar to SARS-CoV Mpro, with a 96% sequence similarity, making it a potential candidate for SARS-CoV-2 [197]. The combination of nirmatrelvir and ritonavir can improve the half-life of nirmatrelvir by inhibiting CYP3A4, which is responsible for the metabolism of nirmatrelvir [178]. A phase II–III double-blind, randomized clinical trial (*n* = 2246) demonstrated that nirmatrelvir–ritonavir can lower the risk of progression to severe COVID-19 by 89% [198]. Another study suggested that treatment with nirmatrelvir–ritonavir on day 0–1 after the onset of symptoms significantly reduced the risk of 28-day all-cause mortality and hospitalization when compared to the delayed treatment of more than two days [199]. 

On the other hand, another team found that Paxlovid was only effective against patients who are >65 years old, while no significant effect was observed in younger patients in the Omicron surge [200]. This raised the concern of possible drug resistance of the SARS-CoV-2 variant, especially Omicron variants, to Paxlovid. A study was performed to evaluate the effect of nirmatrelvir on Omicron-infected Calu-3 cells. The group found that nirmatrelvir can completely suppress replication of the SARS-CoV-2 wildtype, but Omicron still replicated at a low level [201]. However, the group indicated that nirmatrelvir can suppress the residual replication of Omicron [201]. Moreover, several in vitro and cohort studies found that the efficacy of nirmatrelvir was lowered with the SARS-CoV-2 variant. However, it was still considered to be effective against the variants of concern (VOCs), including BA.1, BA.2, BA.4, BA.5, BA.2.75, and XBB sub-lineages [202,203,204,205,206,207,208,209]. It is also effective against the recent variant of interest, BA.2.86, in vitro [35]. In a large-scale in vitro experiment, 53 independent viral lineages with mutations at 23 different residues of the 3CL protease (also known as Mpro) of SARS-CoV-2 revealed that several mutation pathways with precursor mutations of T21I, P252L, or T304I led to a low-level resistance to Paxlovid. The strongest resistance was found to be associated with the E166V mutation, where 100-fold changes in the IC50 value were observed when compared to wildtype, but such mutation will result in a loss of viral replicative fitness [210]. With the aid of the structural method, the same group demonstrated the possible alternations in the substrate-binding pocket of M protease of SARS-CoV-2 that leads to the resistance of Paxlovid. Alternation in the S1 and S4 subsites can lower the inhibitor-binding level, while alternations in S2 and S4’ subsites can enhance the activity of M protease [211]. 

Despite the treatment effect of Paxlovid, cases of viral rebound were found 2–8 days after the completion of Paxlovid [212]. The viral rebound had a positive result in viral antigen or quantitative reverse transcriptase polymerase chain reaction (RT-qPCR) tests after treatment. A retrospective cohort study of electronic health records from a database in the US with a study population of 13,644 patients, who were treated with Paxlovid (*n* = 11,270) or Molnupiravir (*n* = 2374), reported a 7-day and 30-day rebound rate of 3.53% and 5.40%, and symptom rebound rate of 2.31% and 5.87% after Paxlovid treatment, respectively, and the team found no significant differences in rebound rates between Paxlovid and Molnupiravir [213]. A phase II–III, double-blind, randomized, controlled trial was conducted with 2246 unvaccinated patients that were treated with Paxlovid or placebo [214]. A rebound rate of 2.3% and 1.7% was observed in the Paxlovid group and placebo group, respectively. In addition, retrospective cohort studies on viral rebound in Hong Kong indicated that the viral load rebound rates were similar in patients with or without antiviral treatment, and there was no association between the rebound rate and adverse clinical outcomes [215,216]. Although the mechanism of rebound is still unclear, researchers proposed that it is more likely to be the timing of receiving Paxlovid and variation in pharmacokinetics than the resistance to Paxlovid causing the rebound [215,217]. Studies have shown that no Paxlovid resistance has yet been identified in the rebound individuals, suggesting that a longer drug exposure may be needed [217,218,219].

### 6.2. Immunomodulators

#### 6.2.1. Baricitinib (JAK Inhibitor)

Baricitinib is a Janus Kinase (JAK) inhibitor, which inhibits JAK1/2 in the JAK/STAT signaling pathway. The FDA approved Baricitinib for treating COVID-19 in hospitalized adults requiring supplemental oxygen, extracorporeal membrane oxygenation, or non-invasive or invasive mechanical ventilation in 2022. The JAK/STAT signaling pathway plays an important role in the immune response, the T-cell differentiation, the proliferation of CD8 T-cells, and interleukin signaling pathways [220,221]. Although it reduces the hyperinflammatory condition caused by the infection, inhibiting the JAK/STAT pathway can prevent the patient from hyperinflammatory conditions that may lead to death. Initially, it was used for treating immune-mediated diseases, such as rheumatoid arthritis. However, Baricitinib can reduce the COVID-19 hyperinflammation condition after drug repurposing [178]. The administration of Baricitinib is commonly 4 mg/day, but no recommended dosage was suggested. However, renal impairment would greatly affect the dosage of Baricitinib, as the kidney is the major route of excretion. Another study suggested that the dosage should be reduced to 2 mg when the estimated glomerular filtration rate (eGFR) is 30–60, while Baricitinib administration should be stopped when eGFR is lower than 30 [222].

Clinical studies have been performed to assess the efficacy and safety of Baricitinib in COVID-19 patients. Phase III randomized studies demonstrated the safety and efficacy of Baricitinib in COVID-19 patients with a reduction in mortality of hospitalized adults [223,224]. Moreover, researchers found that Baricitinib reduced the absolute mortality rate of older adults aged >70 who suffered from severe COVID-19 pneumonia [225,226].

The safety concern is mainly due to the immunosuppressive effect of Baricitinib as a JAK inhibitor, causing patients to be more susceptible to new infections. Randomized trials examined the safety of Baricitinib use in combination with Remdesivir. The ACTT-2 randomized trial indicated that the combination of Baricitinib plus Remdesivir in hospitalized COVID-19 patients had fewer adverse events and improved efficacy over Remdesivir alone [186]. Another randomized trial, ACTT-4, revealed that the combination of Baricitinib plus Remdesivir had significantly fewer adverse events than the Dexamethasone plus Remdesivir group in hospitalized COVID-19 patients [227]. However, the National Institutes of Health (NIH) indicated that these clinical trials only revealed the safety use of Baricitinib in the short term, and they lacked information on significant safety signals. In addition, the Bari-SolidAct trial on critically ill COVID-19 patients, including vaccinated patients, found a possible safety signal of Baricitinib in vaccinated patients who were older, with more comorbidities [228]. In addition to Baricitinib plus Remdesivir, a recent cohort study compared the efficacy of Baricitinib plus 6-methylprednisolone pulses and standard of care, Dexamethasone plus Remdesivir, on critically ill COVID-19 patients [229]. The Baricitinib group significantly reduced the mortality, with a lowered inflammatory marker level, which suggested another combination strategy for the hyperinflammatory COVID-19.

#### 6.2.2. Tocilizumab (IL-6 Inhibitor)

Tocilizumab is a repurposed humanized immunoglobulin G1K subclass monoclonal antibody, which targets both the interleukin 6 (IL-6) soluble receptor and IL-6 membrane receptor, inhibiting the IL-6 receptor-mediated signal transduction pathways [230,231]. Previously, Tocilizumab was used for treating rheumatoid arthritis, giant cell arteritis, and systemic juvenile idiopathic arthritis, and was found to be effective in alleviating the cytokine release syndrome (CRS) [230,231,232]. Tocilizumab administration can be injected either intravenously (IV) or subcutaneously (SC) in other treatments. On 21 December 2022, the FDA approved the IV injection of Tocilizumab for in-hospital COVID-19 patients who were receiving systemic corticosteroids and required mechanical ventilation, non-invasive ventilation, etc. A single IV dose of 400–800 mg and an initial weight-based dose of 8 mg/kg for COVID-19 patients is recommended by the NIH. However, the dosing strategies still varied in different studies and based on the clinicians’ decisions [230]. A cohort study compared the efficacy in terms of administration strategies and dosing of Tocilizumab, and no significant difference was found between the fixed-dose, 400 mg IV, and 162 mg SC of Tocilizumab [233]. However, the efficacy between weight-based dosing and fixed dosing is still unclear [234]. During the COVID-19 pandemic, researchers found that the IL-6 was correlated to the severity of COVID-19, and the disruption of the IL-6 and its receptor binding can reduce the inflammatory response and cytokine release in COVID-19 patients [235]. In a randomized clinical trial (*n* = 4116), patients with hypoxia and systemic inflammation were administered Tocilizumab or usual care, and Tocilizumab enhanced the 28-day hospital discharge with a reduced rate of reaching the composite endpoint of invasive mechanical ventilation or death [236]. A randomized, multifactorial, adaptive platform trial was performed on adult patients (*n* = 755) with COVID-19 and organ support in the intensive care unit, and Tocilizumab, sarilumab, or standard care was administered [237]. Tocilizumab was shown to improve the survival rate in the 90-day survival analysis. This indicates the importance of IL-6 and COVID-19 disease severity and mortality, and the IL-6 inhibitor is the potential therapeutic strategy for severe COVID-19 patients. 

Recently, a cohort study demonstrated that the use of Tocilizumab significantly reduced delirium or coma in critically ill COVID-19 patients (*n* = 253) [238]. The study was developed based on the preclinical studies performed by the same group, which revealed the association of IL-6 levels and a delirium-like phenotype in animal models [239,240,241]. The study also proposed that the IL-6 trans-signaling pathway contributes to delirium pathogenesis. However, future studies are needed to evaluate the contribution, as prior studies have demonstrated a strong association between acute lung injury and the risk of delirium [242,243].

Moreover, there were concerns regarding the safety of Tocilizumab administration, including secondary infection, thrombosis risk, and increased D-dimer levels [244,245,246]. Regarding thrombosis risk, a multicenter cohort study was performed on critically ill COVID-19 patients (*n* = 867) who were admitted to the ICU, and patients were divided into 2 sub-cohort groups based on the Tocilizumab use within 24 h. The study reported no significant differences in the thrombosis events and D-dimer levels between the Tocilizumab and control groups [247]. Moreover, the study indicated that the use of Tocilizumab significantly reduced the fibrinogen level, 30-day, and in-hospital mortality when compared to the control group [247]. In addition, it is expected that the use of Tocilizumab would inhibit the immune system, as IL-6 mediates the proliferation and differentiation of T cells and B cells. However, evidence of Tocilizumab administration and secondary infection was inconsistent among different studies [248,249,250]. A cohort study performed in Taiwanese hospitals (*n* = 453) indicated that the higher prevalence of secondary infection may be due to the tendency to prescribe Tocilizumab to older patients who are more susceptible to a secondary infection [251]. The same group also found that heavy drinkers and females with COVID-19 were more vulnerable to secondary infection; however, further studies are needed to confirm these findings [251].

### 6.3. Cell Therapy

#### 6.3.1. T Cell Therapy

**Figure 9:**
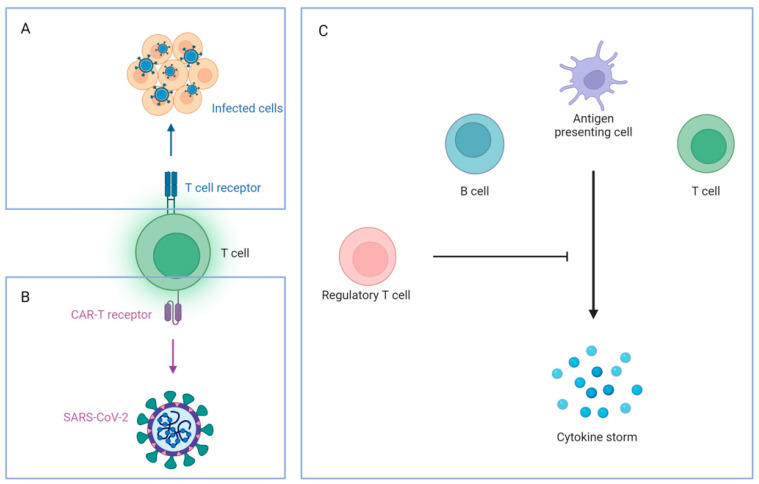
Three types of T cell therapies. (**A**) SARS-CoV-2-specific T cells, (**B**) CAR-T cells, and (**C**) regulatory T cells. This figure was created with BioRender (https://biorender.com; accessed on 11 June 2024).

T cell therapy uses T cells to recognize viral proteins or mediate the immune response. Figure 9 shows three types of T cell therapy, which are SARS-CoV-2-specific T cells that target the structural antigen of the membrane protein, chimeric antigen receptor (CAR-T) cells that target the antigen N protein, and the regulatory T cells that suppress the hyperinflammation condition caused by COVID-19 [252].

Previously, adoptive therapy using the virus-specific T cells (VST) was shown to be effective against viral infection in immunocompromised patients, including patients under immunosuppressive therapy, or post-transplantation [253,254]. The common approaches are to produce the CD45RA memory T cells and SARS-CoV-2-specific cytotoxic T lymphocytes. The SARS-CoV-2-specific T cells can be produced either by using the interferon-γ (IFN-γ) cytokine capture system (CCS) or ex vivo expansion. In the IFN-γ CCS, the peripheral blood mononuclear cells (PBMC) of convalescent COVID-19 donors are stimulated with viral proteins; hence, the IFN-γ CD4 and CD8 T cells are identified by flow cytometry and enriched by using the CCS with a rapid production in 12–24 h [255]. In the ex vivo expansion, SARS-CoV-2-specific T cells are obtained by exposing the donor’s CD4 and CD8 T cells to SARS-CoV-2 antigens, including nucleocapsid protein, spike protein, envelope protein, and membrane antigens, then interleukins 2/4/7 are added to enhance the proliferation of specific T cells.

Several criteria should be considered regarding the effect and safety of the manufactured T cells. Regarding the safety of the adoptive transfer, researchers indicated that the donor–recipient compatibility, including the human leukocyte antigen (HLA) match, in graft-versus-host disease (GVHD) are associated with the safety and efficacy of the therapy, while one of the approaches to reduce the GVHD is to deplete the naive CD45RA T cells [255,256]. Immunologic memory is another consideration, which can determine the duration of the therapy [257].

Several clinical trials had been performed on SARS-CoV-2-specific T cells. A randomized (2:1) phase I/II clinical trial of partially HLA-matched, SARS-CoV-2-specific T cells, in combination with standard care, on patients with severe COVID-19 was shown to be safe and feasible. However, the study also suggested that a larger scale of trial is needed to support the clinical benefit on severe COVID-19 patients [258]. A phase II randomized, multi-center study on memory T cells in COVID-19 patients (*n* = 84) with severe acute respiratory syndrome found that the adoptive therapy can modulate the immune system, leading to acceleration in total lymphocyte recovery on days 3 and 14 [259]. The group also reported that no adverse events and a decrease in CXCL10 levels were observed in the experimental group. In addition, the findings in terms of safety and efficacy were consistent with the randomized trial mentioned above [258,259]. Recently, another phase I/II clinical trial used SARS-CoV-2-specific T cells from the previous trial in 2020 to treat 13 adult and pediatric COVID-19 patients, demonstrating a shorter time to viral clearance with the SARS-CoV-2-specific T cells when compared to comparator cohorts on Remdesivir in the same study period and hospital [260]. The group also indicated that the response of SARS-CoV-2-specific T cells may vary depending on the donor and recipient’s HLA match.

Other than the efficacy of the therapy, its effectiveness against the SARS-CoV-2 variants is also considered. Researchers found that the majority of T cell epitopes were highly conserved among the different variants, while 72% of CD4 cell epitopes and 86% of CD8 cell epitopes were still completely conserved in the Omicron variant [261]. In addition, the selection of desired donors who were vaccinated or convalescent allowed the yield of functional and specific T cells against the newly found variants [260].

Previously, CAR-T therapy used receptor-modified CAR-T cells to target cancer cell antigens. However, it is proposed that CAR-T cells can be designed to target viral antigens, especially the N protein of SARS-CoV-2 [262]. The same can be applied on the regulatory T cells, where the regulatory T cells are modified with CAR. It is proposed that the CAR-regulatory T cells can exhibit an immunosuppressive effect in specific sites [263]. Preclinical models of CAR regulatory T cell therapy on autoimmune diseases were shown to be effective [264,265,266]. In addition, a clinical trial on CAR-regulatory T cells in renal transplant recipients is still ongoing (NCT04817774), while this approach has not been used for treating COVID-19 patients.

Regulatory T cells play an essential role in suppressing the immune response and preventing autoimmune disease through the maintenance of self-tolerance. Researchers observed that the balance between Th17 and regulatory T cells may modulate the COVID-19 disease. In addition, Th17 cells initiate the release of IL-17, leading to the initiation of a proinflammatory cytokine cascade and the attraction of neutrophils and macrophages to the site [267,268]. Studies reported that COVID-19 patients had a decrease in regulatory T cells, while an increase in Th17 cells was associated with the disease progression of COVID-19 [269,270]. 

Regarding the protective effect of the regulatory T cells, the adoptive therapy of regulatory T cell transfer has become the potential therapy to modulate the immune response in severe COVID-19 patients. Clinical trials have been performed to evaluate the effectiveness and safety of this therapy. A randomized and double-blinded phase I trial demonstrated the safety and efficacy of using allogenic cord blood regulatory T cells, CK0802, for COVID-19-associated acute respiratory distress syndrome [271]. In this trial, the circumstances in patients with acute respiratory distress syndrome (ARDS) were improved, with lowered proinflammatory cytokine levels, and no significant HLA-associated reaction was observed. Another clinical trial (NCT06052436) on the use of allogeneic regulatory T cells from thymic tissue to treat severe COVID-19 and ARDS is still ongoing. 

Besides the infusion of regulatory T cells, low-dose IL-2 therapy has been used in clinical trials in autoimmune diseases [272,273,274]. The IL-2 plays a role in the maturation and development of the regulatory T cells through the STAT5 signaling pathway [263,267]. The trials have shown the effect of low-dose IL-2 in enhancing the development of regulatory T cells. Based on that, several clinical trials have been performed to evaluate the effect of IL-2 on COVID-19 patients (NCT04724629 and NCT04357444). However, researchers found that there is a high concentration of soluble IL-2 receptors in severe COVID-19 patients, which may restrict the development of regulatory T cells and the effect of IL-2 [275]. The clinical benefit of regulatory T cells is still under investigation, and more clinical trials and studies are needed for this potential therapeutic strategy.

#### 6.3.2. Mesenchymal Stem Cell (MSC) Therapy

MSC is an adult stem cell that can be isolated from umbilical cord blood and the placenta. MSC exhibits various properties, such as immunomodulatory effects to suppress hyperinflammation and enhance the development of regulatory T cells, promoting tissue regeneration and antiapoptotic effects in COVID-19 patients [276]. The safety and efficacy of MSC therapy were demonstrated by clinical trials in various diseases, such as autoimmune disease, cardiovascular disease, and graft-versus-host disease [277,278,279]. 

Moreover, the MSC can be derived from different sources, including the umbilical cord (UC), placenta, Wharton’s jelly, bone marrow, and adipose tissues. Various clinical trials have been carried out on the efficacy and safety of MSC from different sources in COVID-19 patients [280,281,282,283,284]. 

In a phase I clinical trial, the use of UC-MSC therapy on COVID-19-induced ARDS patients (*n* = 20) was shown to be safe and effective in enhancing the SPO2/FIO2 and serum C-reactive protein (CRP) levels when compared to the standard care group [285]. Another randomized clinical trial (*n* = 40) reported that the use of UC-MSC improved the survival rate by 2.5 times when compared to a control group without any MSC-related adverse events [283]. In contrast to the above clinical trials, a randomized, double-blind trial (*n* = 100) showed no significant difference between the MSC group and placebo group in terms of SPO2/FIO2 [286]. In addition, a one-year follow-up study on severe COVID-19 patients who received MSC therapy showed that the symptoms and levels of KL-6 were significantly lower when compared to the control group over one year [287]. However, the effect of MSC therapy was not significant when the treatment time was extended to two years [288]. Findings from clinical studies suggest that MSC infusion is a safe and potential therapy for COVID-19 patients; however, larger clinical trials and studies are needed for further evaluation of MSC [289].

Besides the therapeutic effect, the limitations of MSC therapy should also be considered. The treatment efficacy of MSC depends on the viability of MSC at the disease site. Several risk factors can contribute to the viability of MSC, including the handling during in vitro expansion, immune response, and undesired differentiation [276,290]. 

## 7. Vaccines

### 7.1. Impact of Vaccines on Epidemiology of COVID-19

Initially, the primary strain of SARS-CoV-2 was isolated, and clinical trials of the COVID-19 vaccines were conducted. The mRNA vaccine BNT162b2 showed up to 95% of protective efficacy of preventing symptomatic infection, while the protein subunit vaccine showed 90% [291,292]. Both vaccines showed almost full protection against hospitalization and death [291,292]. However, the constant mutation of SARS-CoV-2 forms newer SARS-CoV-2 variants that cause breakthrough infections. Maintaining a high neutralizing antibody titer is the key to providing protection based on clinical trials, but the titer degrades rapidly over time [293,294]. One of the more recent Omicron variants of SARS-CoV-2 is JN.1, and it has demonstrated the ability to evade neutralizing antibodies [295]. It indicates that the vaccine efficacy is diminished. However, a new mRNA Omicron XBB.1.5 booster was approved and found to elicit a significant increase in the neutralizing antibody titer to the JN.1 variant of SARS-CoV-2 [296]. The new vaccine booster can counteract the rising variants of SARS-CoV-2. Moreover, the hospitalization and death rates have been stable in the vaccinated population. 

A study conducted in the US between the Alpha-predominant period to the Omicron BA.4/BA.5-predominant period showed that the number of hospitalized patients greater than 75 years old increased from 11% to 33% [297]. In the same period, hospitalized patients suffering from immunocompromised conditions increased from 17% to 28% [297]. These patients have increased risk factors for having severe COVID-19 in the rise of the Omicron variant [298]. However, the overall disease severity and death have decreased over time after vaccination [297]. This proves that vaccination can reduce the severity and mortality of COVID-19. 

### 7.2. Disparities in COVID-19 Response between High-Income and Low-Income Countries

Vaccination is crucial in defending against COVID-19. Wealthy countries, such as the US, have no financial problem in accessing vaccines. The US can afford to have mass vaccination sites and have massive stocks of vaccines for US citizens [299]. Moreover, the US has the technology to support digital tools for analyzing geographical distribution, vaccination status in real time, and demographic information [300]. This allows for efficient distribution of vaccines.

However, not every country can afford the high cost of vaccines set by the manufacturers, especially the low-income countries in Africa. To aid the vaccine distribution in low-income countries, COVID-19 Vaccines Global Access (COVAX) was created [301]. By the end of 2023, COVAX had successfully delivered 2 billion doses of COVID-19 vaccines and prevented 2.7 million deaths in low-income countries [302]. Although the problem of securing the supply of vaccines is solved, low-income countries still encounter a handful of problems. One major problem is the distribution of vaccines. As African countries have variable infrastructures in healthcare, the variance can impact the efficiency in the distribution of vaccines. To adapt to this difficulty, the COVID-19 vaccines are incorporated into routine immunization programs in healthcare centers and community clinics [303]. Utilizing the existing healthcare networks can help distribute vaccines and make vaccines accessible even in remote areas [303]. Another problem is the transportation of vaccines. The diverse geography of Africa poses challenges in transportation. The limited road infrastructure in remote and rural areas requires specialized transportation [304]. Collaboration with non-governmental organizations (NGOs) and the use of mobile vaccination units can overcome these transportation challenges [305,306]. Collaboration with NGOs can facilitate transportation and help with delivering vaccines to different areas [306]. The mobile vaccination units can be moved freely and assembled at any time as vaccination production hubs [305]. These two solutions help solve the transportation challenges faced in Africa.

### 7.3. Pfizer-BioNTech COVID-19 Vaccine (mRNA Vaccine)

Due to the emergence of the pandemic, the companies Pfizer and BioNTech together initiated the development of the mRNA vaccine in 2020. The most clinically advanced vaccine of Pfizer/BioNTech is the BNT162b2 vaccine, which consists of nucleoside-modified mRNA (Wuhan-Hu-1) that encodes a full-length transmembrane spike (S) glycoprotein with modification S(K986P/V987P) to ensure it is in the prefusion conformation, formulated in lipid nanoparticles [307,308].

The BNT162b2 vaccine was selected from the BNT162 vaccine candidates based on the efficacy and safety in several preclinical studies and early-phase clinical trials. Phase I/II clinical trials in Germany (NCT04380701) and the US (NCT04368728) with potential vaccine candidates indicated that both BNT162b1 and BNT162b2 showed promising effects on eliciting antibody responses above the convalescent serum and increased Th1 CD4+ T helper cells [309,310]. No serious adverse events were reported, while adverse events were resolved spontaneously in both trials. Moreover, BNT162b2 with a dose of 30 μg was then selected for phase II/III studies due to the lowered incidence and severity of systemic reactogenicity events. The global phase II/III clinical study that randomized 43,548 participants aged 16 years old or above indicated that 2 doses of BNT162b2 can offer 95% protection against COVID-19 in participants, with a similar incidence of serious adverse events to the placebo group [291]. However, a gradual decline in vaccine efficacy and a few cases of withdrawal due to adverse events were observed over six months of follow-up after the clinical study, which suggests the need for a booster [311]. After the evaluation of the efficacy and safety of BNT162b2, the first EUA was granted in December 2020 for individuals aged 16 years old or older.

The protection of the BNT162b2 and homologous booster against variants was evaluated in various studies in different countries. The effectiveness of BNT162b2 was found to be retained against the Beta and Delta variants in several observational studies. The studies conducted in Qatar and the US indicated that the effectiveness reached the highest point, over 90%, against hospitalization after the administration of the second dose of BNT162b2 during the Delta (B.1.617.2) and Beta (B.1.351) dominance [312,313,314,315]. However, a lower effectiveness (~70%) was estimated against the infection with a rapid waning to 20% after 4–5 months. A retrospective cohort study was performed to assess the effectiveness of the BNT162b2 booster in comparison to the two-dose primary series during the Omicron (B.1.1.529) outbreak in Qatar [316]. The effectiveness of the booster against symptomatic Omicron infection and hospitalization was estimated to be 49.4% and 76.5%, respectively. Moreover, the vaccine effectiveness in the Omicron surge was found to be 53% against Omicron in adolescents with two-dose BNT162b2 in Norway, while it was estimated at 30.9% against Omicron-related hospitalization in people with three-dose vaccination in the UK [317,318]. The studies indicated that the booster effect was less effective against the Omicron infection; however, the protection effect was still retained against hospitalization and death. In addition to the three-dose BNT162b2, a randomized phase III clinical trial (*n* = 10,125) reported that the efficacy of third-dose administration was 95.3% against the B.1.617.2 variant, with only low-grade reactogenicity events observed [319].

Due to the emergence of the Omicron variants, BioNTech/Pfizer developed several variant-adapted vaccines as boosters, such as monovalent (BA.1), bivalent (wildtype, BA.1), and another bivalent (wildtype, BA.4/BA.5) vaccines [320]. Moreover, the FDA granted EUA in September 2023 to the updated version of BNT162b2 (2023–2024 formula), which is effective against the Omicron XBB.1.5 strain for people who are at least six months old. According to the CDC, individuals aged five years and older should receive one dose of the updated BNT162b2 vaccine. Additional doses should be administered to children aged 6 months to 4 years and adults aged 65 years and older. The second dose should be administered at least 3 weeks after the first dose, and the third dose should be administered at least 8 weeks after the first dose [321].

Back in 2022, BioNTech/Pfizer had developed and started a phase II/III clinical trial of monovalent and bivalent vaccines against BA.1 variants in adults (*n* = 1846) aged 55 years old or above who had previously received 3 doses of BNT162b2 [322]. The result indicated that variant-adapted BNT162b2 against BA.1 had a similar safety profile to the original BNT162b2 and elicited substantial neutralizing antibodies against the BA.1 and ancestral strain with a dosage of 60 μg [322]. Despite the safety, the vaccine efficacy of this trial was not available due to the insufficient case numbers. However, the clinical trial and other studies reported that neutralizing antibody titers were low against the BA.4/BA.5 variants, indicating the inadequate efficacy of this vaccine against the later strains [323,324]. 

Regarding this, BioNTech/Pfizer developed the bivalent BNT162b2 vaccine against the wildtype and BA.4/BA.5. A phase II/III clinical study was conducted in adults who had received three doses of BNT162b2 to receive either the original or the bivalent (wildtype/BA.4/BA.5) BNT162b2 booster [325]. The study indicated that the bivalent BA.4/BA.5 vaccine as a booster elicited a higher neutralizing response against the BA.5 and BA.2, as well as their sub-lineages BA.4.6, BQ.1.1, BA.2.75.2, and XBB.1, than the original vaccine [325]. A nationwide cohort study indicated the bivalent BA.4/BA.5 or BA.1 as a fourth-dose booster can reduce the rate of hospitalization [326]. Another study in the US estimated that the relative effectiveness of the bivalent BA.4/BA.5 booster provided an additional 50% and 39% protection against the BA.4/BA.5-related critical illness and hospital admission when compared with those who received two-dose wildtype BNT162b2 [327]. The relative effectiveness against XBB-related infection was 56% for hospital admission and 34% against emergency department admission when compared to BA.4/BA.5-related infection. Moreover, an in vitro study indicated that the bivalent vaccine did not elicit a robust neutralization against the new variants, BA.2.75.2, BQ.1.1, or XBB.1, indicating the urge to develop a vaccine booster against the new variants [328]. 

In response to the XBB and sub-lineages, the monovalent XBB.1.5 vaccine was developed and granted the EUA. A phase II/III clinical trial (NCT05997290) is underway to evaluate the safety and immunogenicity of this vaccine in participants who received three doses or a previous BA.4/BA.5-adapted vaccine. In addition, one-month studies on the ongoing phase II/III clinical trial indicated that the overall geometric mean fold rises in neutralizing titers from baseline to one month of the XBB.1.5-adapted vaccine recipients were slightly higher than BA.4/BA.5-adapted vaccine recipients for XBB.1.5 (7.6 vs. 5.6), JN.1 (3.9 vs. 3.5), and BA.2.86 (4.8 vs. 4.9) [329,330]. In addition, a similar safety profile to the original BNT162b2 in recipients was observed. Studies evaluated the XBB.1.5-adapted vaccine’s effectiveness at around 70% against hospitalization and ICU admission in the XBB.1.5 surge [331,332]. In contrast, cohort studies estimated a lowered vaccine effectiveness, to around 40%, in the JN.1 predominance [333,334]. These findings indicated the need to develop the vaccine against the recent variants.

Other than the efficacy of BNT162b2, the related adverse effects were also considered. In the global phase II/III clinical study of BNT162b2, mild-to-moderate effects, such as fatigue, headache, and pain at the injection site, were common among the recipients [291]. However, serious adverse events, such as right axillary lymphadenopathy, paroxysmal ventricular arrhythmia, and right leg paresthesia, were also reported, as well as two deaths due to arteriosclerosis and cardiac arrest, which were not considered to be related to the vaccine. 

In terms of cardiovascular complications, myocarditis and pericarditis were most common among younger males who received BNT162b2. Several studies have been performed to assess the incidence of myocarditis and pericarditis in recipients with different doses of BNT162b2. In Israel, a study based on the database of Clalit Health Services was conducted with around 2.5 million vaccine recipients, and the incidence of myocarditis in people with 1-dose BNT162b2 was estimated at 2.13 cases per 100,000 persons; however, the highest incidence of 10.69 cases per 100,000 persons was reported in male recipients aged 16–29 years old [335]. Moreover, the European Medicines Agency (EMA) reviewed two European epidemiological studies conducted based on the French national health system and Nordic registry data that estimated the excess risk in younger males after the administration of the second dose of BNT162b2 [336]. The French study indicated that there were around 0.265 extra cases of myocarditis within 7 days after the second administration in 12–29-year-old males per 10,000 when compared to unexposed. Regarding the Nordic study, in a period of 28 days after the second dose, there were 0.56 extra cases of myocarditis in 16–24-year-old males per 10,000 when compared to unexposed. Another study conducted based on the data from Vaccine Safety Datalink (VSD) indicated that, in a period of 7 days after the second dose of BNT162b2, the incidence of myocarditis and pericarditis was 14.3 times higher than the comparison interval, and there were 22.4 extra cases in 18–39-year-old recipients per million doses [337]. In addition, the study also revealed that mRNA-1273 had a higher incidence rate when compared to BNT162b2 and 31.2 extra cases of myocarditis and pericarditis per million doses [337]. However, various studies and the CDC indicated that vaccine-associated myocarditis had a better clinical outcome and less severity when compared to other causes of myocarditis [338,339,340].

On the other hand, blood-coagulation-related adverse events were also reported post-BNT162b2 vaccination. Rare adverse events, such as cerebral venous thrombosis (CVT), thrombocytopenia, and arterial/venous thrombotic events, were reported in various studies. Regarding the arterial and venous thrombotic events, a study observed that 1197 thrombotic events were reported for BNT162b2 recipients of 361,734,967 people who received COVID-19 vaccination, including BNT162b2, mRNA-1273, and AZD1222, while an imbalance of venous (31.8%) and arterial (67.9%) thrombotic events was found. In addition, the incidence of CVT was 0.4% of the 1197 thrombotic events reported in BNT recipients [341]. Regarding thrombocytopenia and hemorrhage, 439 hemorrhagic cases were reported with BNT162b2 and mRNA-1273 out of around 110 million vaccinated people in the US, while 60 and 34 cases of thrombocytopenia were reported with the BNT162b2 vaccination in the UK and US, respectively [342]. It was also postulated that BNT162b2 can induce the spike glycoprotein interaction, causing platelet activation, aberrant activation of alternative pathways of complement, or disruption of endothelial homeostasis, leading to immuno-thrombosis [343]. Moreover, studies indicated that the rates of thrombotic events and CVT were rare for BNT162b2 when compared to other vaccines, such as ChAdOx1 nCoV-19 and Ad26.COV2.S; however, the safety of the vaccine should still be taken into consideration [344,345,346].

Other than the concerns for normal-health individuals, the adverse effects of BNT162b2 in immunocompromised patients were also evaluated by several studies. A cohort study conducted in 136 kidney transplant recipients who received 2-dose BNT162b2 found that the most common side effect was local pain (52.2%), and systemic symptoms (19.2%) were also reported [347]. Similar findings were indicated by another study on 187 transplant recipients who were vaccinated with either BNT162b2 or mRNA-1273, in which no unexpected adverse events were found [348]. In addition, a larger cohort study with 1002 immunocompromised patients with 2-dose BNT162b2 reported no rejection, graft-versus-host disease, or allergy in those immunocompromised patients, including transplant recipients and patients with HIV or lymphoma, while more adverse events were reported in those immunocompetent participants [349]. A recent systematic review of 24 articles including 2838 immunocompromised patients found that no serious adverse events were reported after the fourth dose of BNT162b2 or mRNA-1273 in 13 studies [350]. Collectively, further studies with larger sample sizes should be conducted to examine the rare adverse events, while the above studies also suggested that immunocompromised patients shared a similar safety profile as the general population.

### 7.4. Novavax COVID-19 Vaccine (Protein Subunit Vaccine)

During the COVID-19 pandemic, Novavax developed an adjuvanted protein subunit vaccine for SARS-CoV-2. The vaccine candidate was assigned the codename NVX-CoV2373. In 2021, NVX-CoV2373 was granted emergency use listing (EUL) from the WHO and was later sold under the brand names Nuvaxvoid and COVOVAX [351]. NVX-CoV2373 is now available in more than 45 countries worldwide and is being used either as a two-dose primary vaccination series or as a booster dose [352]. It is noteworthy that possibly due to the prevalence of the Omicron XBB.1.5 subvariant in the US during 2023 and the reduced neutralizing antibody response induced by prototype COVID-19 vaccines against XBB subvariants [353], the FDA amended the EUA approval of the Novavax vaccine in October 2023 [354]. Since then, only the vaccine with an updated formula (original spike protein designed from Wuhan-Hu-1 isolate replaced by spike protein of XBB.1.5 [355], codename NVX-CoV2601) is authorized to be used in individuals with an age of 12 years old or older in the US. In June 2024, Novavax submitted an application for EUA approval of its latest JN.1 vaccine (codename NVX-CoV2705) [356]. This vaccine was claimed to be effective in stimulating the production of broad cross-neutralizing antibodies against multiple variants, including JN.1, KP.2, and KP.3, based on nonclinical data support [356]. Further studies are required for assessing the performance of the latest Novavax COVID-19 vaccine.

The Novavax COVID-19 vaccine encompasses two major components, recombinant SARS-CoV-2 spike proteins and the Matrix-M adjuvant. The spike proteins are produced by introducing a full-length spike gene into baculovirus, and then expressing the gene by infecting SF9 moth cells with the recombinant baculovirus [357]. The injection of these synthetic spike proteins into recipients can lead to an adaptive immune response, due to the ‘foreign’ nature of the proteins introduced. The Matrix-M adjuvant is injected along with the recombinant spike proteins. It comprises saponin, phospholipids, and cholesterol [357]. The major functions of Matrix-M are recruiting antigen-presenting cells to the inoculum site and facilitating antigen presentation in lymph nodes [358,359].

Regarding NVX-CoV2373, two large-scale, placebo-controlled phase III clinical trials conducted in the UK (NCT04583995) and in the US and Mexico (NCT04611802) have provided valuable data on the efficacy and safety of this vaccine. Both studies evaluated the performance of NVX-CoV2373 after a two-dose regimen administered to participants aged 18 years and above.

In terms of vaccine efficacy, according to the data collected from the UK clinical trial, NVX-CoV2373 had an efficacy of 89.7% against virologically confirmed COVID-19 cases [360]. Specifically, it was 86.3% effective against the Alpha (B.1.1.7) and 96.4% non-Alpha variants. For the clinical trial held in the US and Mexico, NVX-CoV2373 was reported to have an overall efficacy of 90.4% [292]. In particular, the vaccine efficacy was 93.6% against the Alpha variant and 92.6% against any VOI or VOC (predominantly Beta (B.1.351), Gamma (P.1), Epsilon (B.1.427 and B.1.429), and Iota (B.1.526) at the times and locations where the trial was conducted). Nevertheless, scientists have expanded the scope of the clinical trial NCT04611802 to examine the performance of NVX-CoV2373 in adolescents aged 12 to 17 years in the US, following the earlier study on adult participants. The data from the adolescent trial suggested an overall efficacy of 79.5% against confirmed COVID-19 cases in this age group [361]. Particularly, it had an efficacy of 82% against the Delta (B.1.617.2) variant. These data together demonstrate the high vaccine efficacy of NVX-CoV2373 against multiple prevalent SARS-CoV-2 variants at the time the trials were conducted.

Aside from efficacy, safety is also an important concern when evaluating vaccine performance. Both phase III clinical trials conducted on adult participants reported that the solicited local and systemic adverse events experienced by vaccinated participants were mostly mild to moderate and transient [292,360]. The clinical trial carried out in the US and Mexico reported a slightly higher frequency of unsolicited adverse events in the vaccinated group compared to the placebo group (16.3% vs. 14.8), yet various unsolicited adverse events were balanced between the two groups. The clinical trial held in the UK also recorded a similar phenomenon, with elevated occurrence of unsolicited events in both the vaccinated and placebo groups (25.3% vs. 20.5%). The reported mortality rate (0.5%, the US and Mexico trial) or number of deaths (1 volunteer, the UK trial) was the same across the two groups of participants. NVX-CoV2373 was not only proven to be safe for use in adults, but also safe for adolescents. Data from the expanded NCT04611802 trial suggested that the severity and duration of solicited adverse events were mostly mild to moderate and self-limited in the vaccinated adolescents. Unsolicited adverse reactions were also balanced across the two treatment groups and no death was recorded [361].

In these studies, the most frequently reported solicited adverse events were injection-site pain, tenderness, headache, and fatigue. Besides, cardiovascular complications, such as myocarditis, pericarditis, and myopericarditis, have been a major concern for some vaccine recipients. Indeed, these adverse events are possible after the injection of NVX-CoV2373, and it was hypothesized that the molecular similarity between SARS-CoV-2 spike protein and the host self-antigen alpha-myosin would lead to direct injury in the myocardium [362]. However, data from clinical trials indicated a low likelihood for these events to occur. Fix et al. [352] performed a benefit–risk assessment based on the results of the NCT04611802 and NCT04583995 trials. In their study, vaccination of NVX-CoV2373 was estimated to prevent 1805 COVID-19 cases, while potentially causing 5.3 excess cases of the aforementioned heart inflammation types per 100,000 vaccinated individuals. More recently, a study reported that among 69,227 NVX-CoV2373 doses delivered in the US, only 2 cases of pericarditis were recorded [363]. In addition, a real-world safety study held in Australia revealed that the reported rates of myocarditis and pericarditis per 100,000 NVX-CoV2373 doses delivered were 1.9 and 19.4 [364], respectively. Taken together, clinical trials and real-world data suggest a low incidence rate of these particular heart inflammation types among NVX-CoV2373 recipients. While blood coagulation disorders, thrombotic events, and Guillain–Barré syndrome have also been characterized in some COVID-19 vaccine receivers [346,365], findings from clinical trials and real-world studies suggest that only a small number or no such cases were associated with the injection of NVX-CoV2373 [292,361,363,364]. Regarding the safety of NVX-CoV2373 in immunocompromised patients, limited data are available, since immunocompromised populations are usually excluded from trials [366]. One study suggested that immunocompromised COVID-19 vaccine recipients share similar rates of adverse events with the general population and no additional adverse effect has been characterized [366]. It is noteworthy that there is an ongoing trial (EUPAS104622) that aims to evaluate the safety of using NVX-CoV2373 in immunocompromised patients [367]. Further studies for this population are needed to gain a comprehensive understanding of the NVX-CoV2373 safety profile.

While NVX-CoV2373 is not authorized to be used in the US at present, it is still available to the public as a primary vaccination series and a booster dose in some other countries, such as Canada [368]. In Canada, a booster dose of NVX-CoV2373 can be administered to recipients aged 18 years and above, about 6 months after the second primary vaccine dose. Several studies have evaluated participants’ immune response after receiving NVX-CoV2373 as a booster dose [369,370,371]. In common, they reported a substantial increase in recipients’ antibodies against SARS-CoV-2. A phase II clinical trial (NCT04368988) conducted in the US and Australia assessed recipients’ responses after receiving three doses of NVX-CoV2373 [369]. The clinical data suggested that most of the local and systemic adverse events were mild to moderate and transient in nature. Moreover, robust increases in IgG and mean neutralizing titer (MN50) against SARS-CoV-2 were found among the participants who received the booster dose. Remarkably, the IgG geometric mean titers against SARS-CoV-2 Alpha and Beta variants increased by 33.7-fold and 40.6-fold, respectively, within 28 days following the booster. This study also reported increases in functional ACE2 receptor-binding inhibition titers for Alpha, Beta, Delta, and BA.1 subvariants among the booster recipients. In addition, data from a phase III clinical trial (NCT05463068) revealed that the NVX-CoV2373 booster dose could induce similar seroconversion rates across participants with different vaccination histories (i.e., those who had received mRNA, viral vector, or protein subunit COVID-19 vaccines previously) [371]. Furthermore, induced antibodies were found to be highly reactive even against the immune-evasive BA.1 and BA.5 subvariants. No severe adverse event or death was reported during the 28 days of follow-up after the booster administration. Collectively, these data suggested the potency and safety of NVX-CoV2373 as a booster in inducing an antibody response against infection caused by particular SARS-CoV-2 variants.

Except for clinical trials, several studies have been established in various locations to assess the real-world effectiveness of NVX-CoV2373 in recipients aged 12 years or above. A retrospective study conducted in Germany and published in April 2024 reported that NVX-CoV2373 was estimated to protect about 95% of recipients (in particular, 96% protection rate after the primary course and 93% protection rate after the booster dose) from COVID-19 [372]. This conclusion was made based on the results obtained from a 10-month follow-up period, and Alpha, Delta, and early Omicron variants were circulating during the study period. Mateo-Urdiales et al. [373] performed an observational study in Italy during the dominance of BA.2 and BA.5 subvariants. While they commented that NVX-CoV2373 can offer protection against SARS-CoV-2 infection, they reported a lower estimated effectiveness of NVX-CoV2373 as a primary series compared to the findings from the German study. With a follow-up median of 156 days (starting from the day of receiving the first dose), the adjusted estimated effectiveness of NVX-CoV2373 against notified and symptomatic COVID-19 was found to be 31% and 50%, respectively, in those who received full vaccination (regarded as 15 days or more after the second dose). The difference in estimated effectiveness of the NVX-CoV2373 primary course between the German and Italian studies may be attributed to various factors, including methodology heterogeneity, differences in the dominating SARS-CoV-2 variants, and participants’ demographics. While the current number of studies examining the effectiveness of NVX-CoV2373 in the general population is limited [374], further research is required to provide a more objective illustration of the performance of NVX-CoV2373 in real-world settings. Besides, some studies have compared the effectiveness of NVX-CoV2373 with other vaccine types, such as the mRNA vaccine BNT162b2. An Australian study revealed that recipients of the NVX-CoV2373 primary series or booster dose had higher propensities for SARS-CoV-2 infection caused by BA.1 or BA.2 subvariants compared to those who received the ancestral BNT162b2 (adjusted hazard ratio: 1.70 for the primary series and 1.39 for the booster dose) [375]. Similarly, another study held in South Korea during the dominance of BA.1, BA.2, and BA.4/5 subvariants also suggested a higher SARS-CoV-2 infection rate in adolescents who had completed the NVX-CoV2373 primary course than those who had received BNT162b2 (incidence risk ratio: 1.46) [376]. The difference in effectiveness between these two vaccines was believed to be attributed to a lower induction rate of spike-specific CD8 T cells by NVX-CoV2373 [376], which consequently led to a weaker cellular immune response against SARS-CoV-2 infection in NVX-CoV2373 recipients.

Regarding the use of NVX-CoV2601 in the US, currently, the CDC recommends its use for individuals with an age of 12 years old or above [377]. For those who are unvaccinated, a two-dose primary vaccination series can be administered, with an interval of three to eight weeks between the two injections. For individuals who have received one dose of either the original (NVX-CoV2373) or updated (NVX-CoV2601) Novavax vaccine, or any dose but without receiving the NVX-CoV2601 formulation, a dose of NVX-CoV2601 can be received as a booster. In addition, one or multiple booster doses of NVX-CoV2601 can be administered to individuals 5 through 64 years of age (immunocompromised), or those aged 65 years and older (regardless of their immunocompromised status). While numerous clinical trials (NCT05975060, NCT05973006, and NCT05925127) aiming to examine the performance of NVX-CoV2601 have been initiated in recent years [378,379,380], some of these studies are still ongoing, or data obtained from the trials have not yet been published in scientific journals. As a result, limited data about the performance and safety of NVX-CoV2601 as a primary vaccination series or a booster dose are now accessible. Further studies are required to critically assess the use of NVX-CoV2601 and reveal the findings to the public.

## 8. Conclusions

With the cooperation of scientists worldwide, early in the pandemic, we were able to gain an in-depth understanding of the novel coronavirus. Since then, there has been an increasing amount of new detection methods and treatment strategies being proposed and released to the market [381]. These have contributed to controlling the spread and lowering the severity of COVID-19. Although COVID-19 is no longer classed as a pandemic, research should be ongoing to focus on COVID-19 vaccines and treatments’ renewal and development. This would ensure their continued effectiveness despite the emergence of new SARS-CoV-2 variants and prevent the deterioration of the current situation of the COVID-19 endemic.

## Figures and Tables

**Table 1:** Five WHO-labeled parent lineage variants of concern during 2020–2022 (outbreak.info, 2024).

WHO Label	Pango Lineage	First Outbreak	Number of Cases Worldwide
Alpha	B.1.1.7	United Kingdom	1,141,525
Beta	B.1.351	South Africa	35,651
Gamma	P.1	Brazil	73,495
Delta	B.1.617.2	India	288,894
Omicron	B.1.1.529	South Africa	1895 *

* Note that Omicron has mutated into many subvariants of itself since the original parent lineage [34].

**Table 2:** A summary table of featured COVID-19 diagnostic methods.

Method	RT-qPCR	ELISA (Antigen)	Rapid Antigen Test (RAT)
Category	Nucleic acid amplification test (NAAT)	Serological test	Serological test
Comment from important organizations	- Gold standard for COVID-19 diagnosis (WHO)	- Acceptable specificity - Can be set as a diagnosis criterion, together with clinical and epidemiological histories and case definition (WHO)	- Several FDA-approved over-the-counter COVID-19 diagnostic tests available in the market - Need to perform repeat testing to prevent obtaining a false-negative result (FDA)
Target for detection	Nucleic acids - Particular regions on the cDNA originated from the ssRNA of the virus	Viral proteins	Viral proteins
Specimens	Nasopharyngeal swab, oropharyngeal swab, saliva, sputum	Nasopharyngeal swab, oropharyngeal swab, saliva, sputum (depends on instructions)	Nasopharyngeal swab, oropharyngeal swab, saliva, sputum (depends on instructions)
Sensitivity	High	Limited	Limited
Specificity	High	Varies	Varies
Result generation time	Varies	Varies	10–15 min
Cost	High	Moderate	Low
Trained operators	Yes	Yes	No
Affected by antigenically different variants	Not likely	Yes	Yes

## References

[1] Centers for Disease Control and Prevention (2024). CDC COVID Data Tracker. [Online]. Centers for Disease Control and Prevention. https://covid.cdc.gov/covid-data-tracker/#variant-proportions.

[2] Tsang H.F., Chan L.W.C., Cho W.C.S., Yu A.C.S., Yim A.K.Y., Chan A.K.C., Ng L.P.W., Wong Y.K.E., Pei X.M., Li M.J.W. (2020). An update on COVID-19 pandemic: The epidemiology, pathogenesis, prevention and treatment strategies. Expert Rev. Anti-Infect. Ther..

[3] Cui J., Li F., Shi Z.-L. (2019). Origin and evolution of pathogenic coronaviruses. Nat. Rev. Microbiol..

[4] Woo P.C.Y., Lau S.K.P., Huang Y., Yuen K.-Y. (2009). Coronavirus Diversity, Phylogeny and Interspecies Jumping. Exp. Biol. Med..

[5] Myint S.H. (1995). Human coronavirus infections. The Coronaviridae.

[6] Vabret A., Mourez T., Dina J., Van Der Hoek L., Gouarin S., Petitjean J., Brouard J., Freymuth F. (2005). Human coronavirus NL63, France. Emerg. Infect. Dis..

[7] Woo P.C.Y., Lau S.K.P., Chu C.-M., Chan K.-H., Tsoi H.-W., Huang Y., Wong B.H.L., Poon R.W.S., Cai J.J., Luk W.-K. (2005). Characterization and Complete Genome Sequence of a Novel Coronavirus, Coronavirus HKU1, from Patients with Pneumonia. J. Virol..

[8] Ogimi C., Kim Y.J., Martin E.T., Huh H.J., Chiu C.H., Englund J.A. (2020). What’s new with the old coronaviruses?. J. Pediatr. Infect. Dis. Soc..

[9] De Groot R.J., Baker S.C., Baric R.S., Brown C.S., Drosten C., Enjuanes L., Fouchier R.A., Galiano M., Gorbalenya A.E., Memish Z.A. (2013). Commentary: Middle East Respiratory Syndrome Coronavirus (MERS-CoV): Announcement of the Coronavirus Study Group. J. Virol..

[10] Peiris J.S.M., Lai S.T., Poon L.L.M., Guan Y., Yam L.Y.C., Lim W., Nicholls J., Yee W.K.S., Yan W.W., Cheung M.T. (2003). Coronavirus as a possible cause of severe acute respiratory syndrome. Lancet.

[11] Luo G., Gao S.-J. (2020). Global health concerns stirred by emerging viral infections. J. Med. Virol..

[12] Ludwig S., Zarbock A. (2020). Coronaviruses and SARS-CoV-2: A Brief Overview. Anesth. Analg..

[13] Coronaviridae Study Group of the International Committee on Taxonomy of Viruses (2020). The species Severe acute respiratory syndrome-related coronavirus: Classifying 2019-nCoV and naming it SARS-CoV-2. Nat. Microbiol..

[14] Fouchier R.A., Kuiken T., Schutten M., Van Amerongen G., Van Doornum G.J., Van Den Hoogen B.G., Peiris M., Lim W., Stöhr K., Osterhaus A.D. (2003). Koch’s postulates fulfilled for SARS virus. Nature.

[15] MacKenzie J.S., Smith D.W. (2020). COVID-19: A novel zoonotic disease caused by a coronavirus from China: What we know and what we don’t. Microbiol. Aust..

[16] Lu H., Stratton C.W., Tang Y.-W. (2020). Outbreak of pneumonia of unknown etiology in Wuhan, China: The mystery and the miracle. J. Med. Virol..

[17] Guo Y.R., Cao Q.D., Hong Z.S., Tan Y.Y., Chen S.D., Jin H.J., Tan K.S., Wang D.Y., Yan Y. (2020). The origin, transmission and clinical therapies on coronavirus disease 2019 (COVID-19) outbreak–an update on the status. Mil. Med. Res..

[18] Horton R. (2020). Offline: 2019-nCoV outbreak-early lessons. Lancet.

[19] World Health Organization (2024). Why Is COVID-19 Data Being Presented as Weekly Statistics? [Online]. World Health Organization. https://data.who.int/dashboards/covid19/cases?m49=156&n=c.

[20] Belser J.A., Rota P.A., Tumpey T.M. (2013). Ocular tropism of respiratory viruses. Microbiol. Mol. Biol. Rev..

[21] Lu Q., Shi Y. (2020). Coronavirus disease (COVID-19) and neonate: What neonatologist need to know. J. Med. Virol..

[22] Li H., Leong F.Y., Xu G., Kang C.W., Lim K.H., Tan B.H., Loo C.M. (2021). Airborne dispersion of droplets during coughing: A physical model of viral transmission. Sci. Rep..

[23] Jiang F., Deng L., Zhang L., Cai Y., Cheung C.W., Xia Z. (2020). Review of the clinical characteristics of coronavirus disease 2019 (COVID-19). J. Gen. Intern. Med..

[24] Chen T. (2021). Fomites and the COVID-19 Pandemic: An Evidence Review on Its Role in Viral Transmission.

[25] Wu D., Wu T., Liu Q., Yang Z. (2020). The SARS-CoV-2 outbreak: What we know. Int. J. Infect. Dis..

[26] Van Doremalen N., Bushmaker T., Morris D.H., Holbrook M.G., Gamble A., Williamson B.N., Tamin A., Harcourt J.L., Thornburg N.J., Gerber S.I. (2020). Aerosol and surface stability of SARS-CoV-2 as compared with SARS-CoV-1. N. Engl. J. Med..

[27] Koenig K.L., Bey C.K., McDonald E.C. (2020). 2019-nCoV: The identify-isolate-inform (3I) tool applied to a novel emerging coronavirus. West. J. Emerg. Med..

[28] Tellier R. (2022). COVID-19: The case for aerosol transmission. Interface Focus.

[29] Zhou C. (2020). Evaluating new evidence in the early dynamics of the novel coronavirus COVID-19 outbreak in Wuhan, China with real time domestic traffic and potential asymptomatic transmissions. MedRxiv.

[30] Morawska L.J.G.R., Johnson G.R., Ristovski Z.D., Hargreaves M., Mengersen K., Corbett S., Chao C.Y.H., Li Y., Katoshevski D. (2009). Size distribution and sites of origin of droplets expelled from the human respiratory tract during expiratory activities. J. Aerosol Sci..

[31] Cortellessa G., Stabile L., Arpino F., Faleiros D.E., Van Den Bos W., Morawska L., Buonanno G. (2021). Close proximity risk assessment for SARS-CoV-2 infection. Sci. Total Environ..

[32] Denison M.R., Graham R.L., Donaldson E.F., Eckerle L.D., Baric R.S. (2011). Coronaviruses: An RNA proofreading machine regulates replication fidelity and diversity. RNA Biol..

[33] Mlcochova P., Kemp S., Dhar M.S., Papa G., Meng B., Mishra S., Whittaker C., Mellan T., Ferreira I., Datir R. (2021). SARS-CoV-2 B. 1.617. 2 Delta variant emergence, replication and sensitivity to neutralising antibodies. BioRxiv.

[34] Kandeel M., Mohamed M.E.M., Abd El-Lateef H.M.A., Venugopala K.N., El-Beltagi H.S. (2022). Omicron variant genome evolution and phylogenetics. J. Med. Virol..

[35] Planas D., Staropoli I., Michel V., Lemoine F., Donati F., Prot M., Porrot F., Guivel-Benhassine F., Jeyarajah B., Brisebarre A. (2024). Distinct evolution of SARS-CoV-2 Omicron XBB and BA.2.86/JN.1 lineages combining increased fitness and antibody evasion. Nat. Commun..

[36] Stehlik P., Dowsett C., Camacho X., Falster M.O., Lim R., Nasreen S., Pratt N.L., Pearson S.A., Henry D. (2024). Evolution of the data and methods in real-world COVID-19 vaccine effectiveness studies on mortality: A scoping review protocol. BMJ Open.

[37] Haider N., Hasan M.N., Guitian J., Khan R.A., McCoy D., Ntoumi F., Dar O., Ansumana R., Uddin J., Zumla A. (2023). The disproportionate case-fatality ratio of COVID-19 between countries with the highest vaccination rates and the rest of the world. IJID Reg..

[38] World Health Organization (2023). Statement on the Fifteenth Meeting of the IHR (2005) Emergency Committee on the COVID-19 Pandemic. [Online]. World Health Organization. https://www.who.int/news/item/05-05-2023-statement-on-the-fifteenth-meeting-of-the-international-health-regulations-(2005)-emergency-committee-regarding-the-coronavirus-disease-(covid-19)-pandemic.

[39] World Health Organization (2023). Statement on the Update of Who’s Working Definitions and Tracking System for SARS-COV-2 Variants of Concern and Variants of Interest. [Online]. World Health Organization. https://www.who.int/news/item/16-03-2023-statement-on-the-update-of-who-s-working-definitions-and-tracking-system-for-sars-cov-2-variants-of-concern-and-variants-of-interest.

[40] Rodriguez H., Hartert T.V., Gebretsadik T., Carroll K.N., Larkin E.K. (2016). A simple respiratory severity score that may be used in evaluation of acute respiratory infection. BMC Res. Notes.

[41] Kaku Y., Yo M.S., Tolentino J.E., Uriu K., Okumura K., Ito J., Sato K. (2024). Virological characteristics of the SARS-CoV-2 KP. 3, LB. 1, and KP. 2.3 variants. Lancet Infect. Dis..

[42] World Health Organization (2024). Coronavirus Disease (COVID-19) Situation Reports. [Online]. World Health Organization. https://www.who.int/emergencies/diseases/novel-coronavirus-2019/situation-reports.

[43] World Health Organization (2024). COVID-19 Deaths WHO COVID-19 Dashboard. [Online]. World Health Organization. https://data.who.int/dashboards/covid19/deaths.

[44] Centers for Disease Control and Prevention (2024). Vaccination Trends-Adults. [Online]. Centers for Disease Control and Prevention. https://www.cdc.gov/respiratory-viruses/data-research/dashboard/vaccination-trends-adults.html.

[45] European Centre for Disease Prevention and Control Interim COVID-19 Vaccination Coverage in the EU/EEA during the 2023–2024 Season Campaigns 2024. [Online]. European Centre for Disease Prevention and Control. https://www.ecdc.europa.eu/sites/default/files/documents/COVID-19-vaccination-coverage-2023%E2%80%9324.pdf.

[46] World Health Organization (2024). COVID-19 Cases WHO COVID-19 Dashboard. [Online]. World Health Organization. https://data.who.int/dashboards/covid19/cases?m49=620&n=o.

[47] Centers for Disease Control and Prevention (2024). COVID-19 Wastewater Data—National Trends. Centers for Disease Control and Prevention. https://www.cdc.gov/nwss/rv/COVID19-nationaltrend.html.

[48] Pulliam J.R., van Schalkwyk C., Govender N., von Gottberg A., Cohen C., Groome M.J., Dushoff J., Mlisana K., Moultrie H. (2022). Increased risk of SARS-CoV-2 reinfection associated with emergence of Omicron in South Africa. Science.

[49] Chen Y., Zhu W., Han X., Chen M., Li X., Huang H., Zhang M., Wei R., Zhang H., Yang C. (2024). How does the SARS-CoV-2 reinfection rate change over time? The global evidence from systematic review and meta-analysis. BMC Infect. Dis..

[50] Guedes A.R., Oliveira M.S., Tavares B.M., Luna-Muschi A., Lazari C.d.S., Montal A.C., de Faria E., Maia F.L., Barboza A.d.S., Leme M.D. (2023). Reinfection rate in a cohort of healthcare workers over 2 years of the COVID-19 pandemic. Sci. Rep..

[51] Department of Health (2023). COVID-19 Reinfection Data. [Online]. Department of Health. https://coronavirus.health.ny.gov/covid-19-reinfection-data.

[52] Wei J., Stoesser N., Matthews P.C., Khera T., Gethings O., Diamond I., Studley R., Taylor N., Peto T.E., Walker A.S. (2024). Risk of SARS-CoV-2 reinfection during multiple Omicron variant waves in the UK general population. Nat. Commun..

[53] Hoeggerl A.D., Nunhofer V., Weidner L., Lauth W., Zimmermann G., Badstuber N., Grabmer C., Kartal O., Jungbauer C., Neureiter H. (2024). Dissecting the dynamics of SARS-CoV-2 reinfections in blood donors with pauci-or asymptomatic COVID-19 disease course at initial infection. Infect. Dis..

[54] de Anda-Jáuregui G., Gómez-Romero L., Cañas S., Campos-Romero A., Alcántar-Fernández J., Cedro-Tanda A. (2024). COVID-19 reinfections in Mexico City: Implications for public health. Front. Public Health.

[55] Antia R., Halloran M.E. (2021). Transition to endemicity: Understanding COVID-19. Immunity.

[56] Akkiz H. (2021). Implications of the novel mutations in the SARS-CoV-2 genome for transmission, disease severity, and the vaccine development. Front. Med..

[57] Andersen K.G., Rambaut A., Lipkin W.I., Holmes E.C., Garry R.F. (2020). The proximal origin of SARS-CoV-2. Nat. Med..

[58] Jiang S., Hillyer C., Du L. (2020). Neutralizing antibodies against SARS-CoV-2 and other human coronaviruses. Trends Immunol..

[59] V’kovski P., Kratzel A., Steiner S., Stalder H., Thiel V. (2021). Coronavirus biology and replication: Implications for SARS-CoV-2. Nat. Rev. Microbiol..

[60] Hoffmann M., Kleine-Weber H., Schroeder S., Krüger N., Herrler T., Erichsen S., Schiergens T.S., Herrler G., Wu N.H., Nitsche A. (2020). SARS-CoV-2 cell entry depends on ACE2 and TMPRSS2 and is blocked by a clinically proven protease inhibitor. Cell.

[61] Fuentes-prior P. (2021). Priming of SARS-CoV-2 S protein by several membrane-bound serine proteinases could explain enhanced viral infectivity and systemic COVID-19 infection. J. Biol. Chem..

[62] Wu J., Deng W., Li S., Yang X. (2020). Advances in research on ACE2 as a receptor for 2019-nCoV. Cell. Mol. Life Sci..

[63] Hanff T.C., Harhay M.O., Brown T.S., Cohen J.B., Mohareb A.M. (2020). Is there an association between COVID-19 mortality and the renin-angiotensin system? A call for epidemiologic investigations. Clin. Infect. Dis..

[64] Shieh W.-J., Hsiao C.-H., Paddock C.D., Guarner J., Goldsmith C.S., Tatti K., Packard M., Mueller L., Wu M.-Z., Rollin P. (2005). Immunohistochemical, in situ hybridization, and ultrastructural localization of SARS-associated coronavirus in lung of a fatal case of severe acute respiratory syndrome in Taiwan. Hum. Pathol..

[65] Chen C., Zhou Y., Wang D.W. (2020). SARS-CoV-2: A potential novel etiology of fulminant myocarditis. Herz.

[66] Li D., Wu M. (2021). Pattern recognition receptors in health and diseases. Signal Transduct. Target. Ther..

[67] Ebbole D.J. (2007). Magnaporthe as a model for understanding host-pathogen interactions. Annu. Rev. Phytopathol..

[68] Streicher F., Jouvenet N. (2019). Stimulation of innate immunity by host and viral RNAs. Trends Immunol..

[69] Ivashkiv L.B., Donlin L.T. (2014). Regulation of type I interferon responses. Nat. Rev. Immunol..

[70] Deng X., Hackbart M., Mettelman R.C., O’brien A., Mielech A.M., Yi G., Kao C.C., Baker S.C. (2017). Coronavirus nonstructural protein 15 mediates evasion of dsRNA sensors and limits apoptosis in macrophages. Proc. Natl. Acad. Sci. USA.

[71] Chen X., Zhao B., Qu Y., Chen Y., Xiong J., Feng Y., Men D., Huang Q., Liu Y., Yang B. (2020). Detectable serum severe acute respiratory syndrome coronavirus 2 viral load (RNAemia) is closely correlated with drastically elevated interleukin 6 level in critically ill patients with coronavirus disease 2019. Clin. Infect. Dis..

[72] Paul B.D., Lemle M.D., Komaroff A.L., Snyder S.H. (2021). Redox imbalance links COVID-19 and myalgic encephalomyelitis/chronic fatigue syndrome. Proc. Natl. Acad. Sci. USA.

[73] Channappanavar R., Perlman S. (2017). Pathogenic human coronavirus infections: Causes and consequences of cytokine storm and immunopathology. Semin. Immunopathol..

[74] Grifoni A., Weiskopf D., Ramirez S.I., Mateus J., Dan J.M., Moderbacher C.R., Rawlings S.A., Sutherland A., Premkumar L., Jadi R.S. (2020). Targets of T cell responses to SARS-CoV-2 coronavirus in humans with COVID-19 disease and unexposed individuals. Cell.

[75] Shi D., Weng T., Wu J., Dai C., Luo R., Chen K., Zhu M., Lu X., Cheng L., Chen Q. (2021). Dynamic characteristic analysis of antibodies in patients with COVID-19: A 13-month study. Front. Immunol..

[76] Zohar T., Alter G. (2020). Dissecting antibody-mediated protection against SARS-CoV-2. Nat. Rev. Immunol..

[77] Vetter P., Eberhardt C.S., Meyer B., Murillo P.A.M., Torriani G., Pigny F., Lemeille S., Cordey S., Laubscher F., Vu D.-L. (2020). Daily viral kinetics and innate and adaptive immune response assessment in COVID-19: A case series. mSphere.

[78] Jin J.M., Bai P., He W., Wu F., Liu X.F., Han D.M., Liu S., Yang J.K. (2020). Gender differences in patients with COVID-19: Focus on severity and mortality. Front. Public Health.

[79] Gupta A., Madhavan M.V., Sehgal K., Nair N., Mahajan S., Sehrawat T.S., Bikdeli B., Ahluwalia N., Ausiello J.C., Wan E.Y. (2020). Extrapulmonary manifestations of COVID-19. Nat. Med..

[80] Mehta P., McAuley D.F., Brown M., Sanchez E., Tattersall R.S., Manson J.J., on behalf of the HLH Across Speciality Collaboration, UK (2020). COVID-19: Consider cytokine storm syndromes and immunosuppression. Lancet.

[81] Singh M.K., Mobeen A., Chandra A., Joshi S., Ramachandran S. (2021). A meta-analysis of comorbidities in COVID-19: Which diseases increase the susceptibility of SARS-CoV-2 infection?. Comput. Biol. Med..

[82] Agrawal U., Azcoaga-Lorenzo A., Fagbamigbe A.F., Vasileiou E., Henery P., Simpson C.R., Stock S.J., Shah S.A., Robertson C., Woolhouse M. (2021). Association between multimorbidity and mortality in a cohort of patients admitted to hospital with COVID-19 in Scotland. J. R. Soc. Med..

[83] Carfì A., Bernabei R., Landi F. (2020). Persistent symptoms in patients after acute COVID-19. JAMA.

[84] Shah W., Hillman T., Playford E.D., Hishmeh L. (2021). Managing the long term effects of COVID-19: Summary of NICE, SIGN, and RCGP rapid guideline. BMJ.

[85] Greenhalgh T., Knight M., A’court C., Buxton M., Husain L. (2020). Management of post-acute COVID-19 in primary care. BMJ.

[86] van Kampen J.J., van de Vijver D.A., Fraaij P.L., Haagmans B.L., Lamers M.M., Okba N., van den Akker J.P., Endeman H., Gommers D.A., Cornelissen J.J. (2021). Duration and key determinants of infectious virus shedding in hospitalized patients with coronavirus disease-2019 (COVID-19). Nat. Commun..

[87] Raveendran A. (2020). Long COVID-19: Challenges in the diagnosis and proposed diagnostic criteria. Diabetes Metab. Syndr. Clin. Res. Rev..

[88] Ceban F., Ling S., Lui L.M., Lee Y., Gill H., Teopiz K.M., Rodrigues N.B., Subramaniapillai M., Di Vincenzo J.D., Cao B. (2021). Fatigue and cognitive impairment in Post-COVID-19 Syndrome: A systematic review and meta-analysis. Brain Behav. Immun..

[89] Ayoubkhani D., Bosworth M.L., King S., Pouwels K.B., Glickman M., Nafilyan V., Zaccardi F., Khunti K., Alwan N.A., Walker A.S. (2022). Risk of Long Covid in people infected with SARS-CoV-2 after two doses of a COVID-19 vaccine: Community-based, matched cohort study. medRxiv.

[90] Subramanian A., Nirantharakumar K., Hughes S., Myles P., Williams T., Gokhale K.M., Taverner T., Chandan J.S., Brown K., Simms-Williams N. (2022). Symptoms and risk factors for long COVID in non-hospitalized adults. Nat. Med..

[91] Han Q., Zheng B., Daines L., Sheikh A. (2022). Long-term sequelae of COVID-19: A systematic review and meta-analysis of one-year follow-up studies on post-COVID symptoms. Pathogens.

[92] Singh I., Joseph P., Heerdt P.M., Cullinan M., Lutchmansingh D.D., Gulati M., Possick J.D., Systrom D.M., Waxman A.B. (2022). Persistent exertional intolerance after COVID-19: Insights from invasive cardiopulmonary exercise testing. Chest.

[93] Fogarty H., Townsend L., Morrin H., Ahmad A., Comerford C., Karampini E., Englert H., Byrne M., Bergin C., O’sullivan J.M. (2021). Persistent endotheliopathy in the pathogenesis of long COVID syndrome. J. Thromb. Haemost..

[94] Li T., Yang Y., Li Y., Wang Z., Ma F., Luo R., Xu X., Zhou G., Wang J., Niu J. (2022). Platelets mediate inflammatory monocyte activation by SARS-CoV-2 spike protein. J. Clin. Investig..

[95] Grobbelaar L.M., Venter C., Vlok M., Ngoepe M., Laubscher G.J., Lourens P.J., Steenkamp J., Kell D.B., Pretorius E. (2021). SARS-CoV-2 spike protein S1 induces fibrin (ogen) resistant to fibrinolysis: Implications for microclot formation in COVID-19. Biosci. Rep..

[96] Ajaz S., McPhail M.J., Singh K.K., Mujib S., Trovato F.M., Napoli S., Agarwal K. (2021). Mitochondrial metabolic manipulation by SARS-CoV-2 in peripheral blood mononuclear cells of patients with COVID-19. Am. J. Physiol.-Cell Physiol..

[97] Tsivgoulis G., Fragkou P.C., Lachanis S., Palaiodimou L., Lambadiari V., Papathanasiou M., Sfikakis P.P., Voumvourakis K.I., Tsiodras S. (2020). Olfactory bulb and mucosa abnormalities in persistent COVID-19-induced anosmia: A magnetic resonance imaging study. Eur. J. Neurol..

[98] Brann D.H., Tsukahara T., Weinreb C., Lipovsek M., Van den Berge K., Gong B., Chance R., Macaulay I.C., Chou H.J., Fletcher R.B. (2020). Non-neuronal expression of SARS-CoV-2 entry genes in the olfactory system suggests mechanisms underlying COVID-19-associated anosmia. Sci. Adv..

[99] Vaira L.A., Hopkins C., Sandison A., Manca A., Machouchas N., Turilli D., Lechien J.R., Barillari M.R., Salzano G., Cossu A. (2020). Olfactory epithelium histopathological findings in long-term coronavirus disease 2019 related anosmia. J. Laryngol. Otol..

[100] Davis H.E., Mccorkell L., Vogel J.M., Topol E.J. (2023). Long COVID: Major findings, mechanisms and recommendations. Nat. Rev. Microbiol..

[101] Català M., Mercadé-Besora N., Kolde R., Trinh N.T., Roel E., Burn E., Rathod-Mistry T., Kostka K., Man W.Y., Delmestri A. (2024). The effectiveness of COVID-19 vaccines to prevent long COVID symptoms: Staggered cohort study of data from the UK, Spain, and Estonia. Lancet Respir. Med..

[102] Watanabe A., Iwagami M., Yasuhara J., Takagi H., Kuno T. (2023). Protective effect of COVID-19 vaccination against long COVID syndrome: A systematic review and meta-analysis. Vaccine.

[103] Mccarthy M.W. (2023). Metformin as a potential treatment for COVID-19. Expert Opin. Pharmacother..

[104] Mccarthy M.W. (2023). Paxlovid as a potential treatment for long COVID. Expert Opin. Pharmacother..

[105] Knopman D.S., Laskowitz D.T., Koltai D.C., Charvet L.E., Becker J.H., Federman A.D., Wisnivesky J., Mahncke H., Van Vleet T.M., Bateman L. (2024). RECOVER-NEURO: Study protocol for a multi-center, multi-arm, phase 2, randomized, active comparator trial evaluating three interventions for cognitive dysfunction in post-acute sequelae of SARS-CoV-2 infection (PASC). Trials.

[106] Zimmerman K. (2024). RECOVER-AUTONOMIC: A Platform Protocol for Evaluation of Interventions for Autonomic Dysfunction in Post-Acute Sequelae of SARS-CoV-2 Infection (PASC). [Online]. https://trials.recovercovid.org/documents/RECOVER_AUTONOMIC_Protocol_V3.0.pdf.

[107] Zimmerman K. (2024). RECOVER-SLEEP: A Platform Protocol for Evaluation of Interventions for Sleep Disturbances in Post-Acute Sequelae of SARS-CoV-2 Infection (PASC). [Online]. https://trials.recovercovid.org/documents/RECOVER_SLEEP_Protocol_V3.0.pdf.

[108] Gao W., Lv J., Pang Y., Li L.-M. (2021). Role of asymptomatic and pre-symptomatic infections in COVID-19 pandemic. BMJ.

[109] Ravindra K., Malik V., Padhi B., Goel S., Gupta M. (2021). Asymptomatic infection and transmission of COVID-19 among clusters: Systematic review and meta-analysis. Public Health.

[110] Jayaweera M., Perera H., Gunawardana B., Manatunge J. (2020). Transmission of Null COVID-19 virus by droplets and aerosols: A critical review on the unresolved dichotomy. Environ. Res..

[111] World Health Organization In Vitro Diagnostics for COVID-19 [Online]. https://www.who.int/teams/health-product-policy-and-standards/assistive-and-medical-technology/medical-devices/priority-medical-devices-for-covid/diagnostics-for-covid-19.

[112] Food and Drug Administration (2023). COVID-19 Test Basics [Online]. https://www.fda.gov/consumers/consumer-updates/covid-19-test-basics.

[113] Castellanos M., Somoza Á. (2022). Emerging clinically tested detection methods for COVID-19. FEBS J..

[114] Maia R., Carvalho V., Faria B., Miranda I., Catarino S., Teixeira S., Lima R., Minas G., Ribeiro J. (2022). Diagnosis Methods for COVID-19: A Systematic Review. Micromachines.

[115] Dong H., Zhang K., Zhang J., Xiao Y., Zhang F., Wang M., Wang H., Zhao G., Xie S., Xie X. (2023). A fast RT-qPCR system significantly shortens the time for SARS-CoV-2 nucleic acid test. Drug Discov. Ther..

[116] Tsang H.F., Leung W.M.S., Chan L.W.C., Cho W.C.S., Wong S.C.C. (2021). Performance comparison of the Cobas^®^ Liat^®^ and Cepheid^®^ GeneXpert^®^ systems on SARS-CoV-2 detection in nasopharyngeal swab and posterior oropharyngeal saliva. Expert Rev. Mol. Diagn..

[117] Tan S.H., Allicock O., Armstrong-Hough M., Wyllie A.L. (2021). Saliva as a gold-standard sample for SARS-CoV-2 detection. Lancet Respir. Med..

[118] Wang K., Zhang X., Sun J., Ye J., Wang F., Hua J., Zhang H., Shi T., Li Q., Wu X. (2020). Differences of Severe Acute Respiratory Syndrome Coronavirus 2 Shedding Duration in Sputum and Nasopharyngeal Swab Specimens among Adult Inpatients with Coronavirus Disease 2019. Chest.

[119] Akowuah E., Acheampong G., Ayisi-Boateng N.K., Amaniampong A., Agyapong F.O., Senyo Kamasah J., Agyei G., Owusu D.O., Nkrumah B., Mutocheluh M. (2022). Comparable Detection of SARS-CoV-2 in Sputum and Oropharyngeal Swab Samples of Suspected COVID-19 Patients. COVID.

[120] Ahmadzadeh M., Vahidi H., Mahboubi A., Hajifathaliha F., Nematollahi L., Mohit E. (2021). Different Respiratory Samples for COVID-19 Detection by Standard and Direct Quantitative RT-PCR: A Literature Review. Iran. J. Pharm. Res. IJPR.

[121] Ali D.Y., Hussein R.A., Elshafie S.M., Mohamed R.A., El Reheem F.A. (2023). Comparable detection of nasopharyngeal swabs and induced sputum specimens for viral nucleic acid detection of suspected novel coronavirus (SARS-Cov-2) patients in Fayoum governorate, Egypt. Beni-Suef Univ. J. Basic Appl. Sci..

[122] Alexandersen S., Chamings A., Bhatta T.R. (2020). SARS-CoV-2 genomic and subgenomic RNAs in diagnostic samples are not an indicator of active replication. Nat. Commun..

[123] Maniruzzaman M., Islam M.M., Ali M.H., Mukerjee N., Maitra S., Kamal M.A., Ghosh A., Castrosanto M.A., Alexiou A., Ashraf G.M. (2022). COVID-19 diagnostic methods in developing countries. Environ. Sci. Pollut. Res. Int..

[124] Jefferson T., Spencer E.A., Conly J.M., Rosca E.C., Maltoni S., Brassey J., Onakpoya I.J., Evans D.H., Heneghan C.J., Plüddemann A. (2023). Viral cultures, cycle threshold values and viral load estimation for assessing SARS-CoV-2 infectiousness in haematopoietic stem cell and solid organ transplant patients: A systematic review. J. Hosp. Infect..

[125] Benevides Lima L., Mesquita F.P., Brasil de Oliveira L.L., Andréa da Silva Oliveira F., Elisabete Amaral de Moraes M., Souza P.F., Montenegro R.C. (2022). True or false: What are the factors that influence COVID-19 diagnosis by RT-qPCR?. Expert Rev. Mol. Diagn..

[126] Udvardi M.K., Czechowski T., Scheible W.-R. (2008). Eleven golden rules of quantitative RT-PCR. Plant Cell.

[127] Panchali M.J.L., Oh H.J., Lee Y.M., Kim C.-M., Tariq M., Seo J.-W., Kim D.Y., Yun N.R., Kim D.-M. (2022). Accuracy of Real-Time Polymerase Chain Reaction in COVID-19 Patients. Microbiol. Spectr..

[128] Bello-Lemus Y., Anaya-Romero M., Gómez-Montoya J., Árquez M., González-Torres H.J., Navarro-Quiroz E., Pacheco-Londoño L., Pacheco-Lugo L., Acosta-Hoyos A.J. (2022). Comparative Analysis of In-House RT-qPCR Detection of SARS-CoV-2 for Resource-Constrained Settings. Diagnostics.

[129] Walsh K.A., Jordan K., Clyne B., Rohde D., Drummond L., Byrne P., Ahern S., Carty P.G., O’Brien K.K., O’Murchu E. (2020). SARS-CoV-2 detection, viral load and infectivity over the course of an infection. J. Infect..

[130] Liu R., Yi S., Zhang J., Lv Z., Zhu C., Zhang Y. (2020). Viral Load Dynamics in Sputum and Nasopharyngeal Swab in Patients with COVID-19. J. Dent. Res..

[131] Zheng S., Fan J., Yu F., Feng B., Lou B., Zou Q., Xie G., Lin S., Wang R., Yang X. (2020). Viral load dynamics and disease severity in patients infected with SARS-CoV-2 in Zhejiang province, China, January–March 2020: Retrospective cohort study. BMJ.

[132] Food and Drug Administration (2024). In Vitro Diagnostics EUAs—Molecular Diagnostic Tests for SARS-CoV-2 [Online]. https://www.fda.gov/medical-devices/covid-19-emergency-use-authorizations-medical-devices/in-vitro-diagnostics-euas-molecular-diagnostic-tests-sars-cov-2.

[133] Johns Hopkins Center for Health Security (2022). Antigen and Molecular Tests for COVID-19 [Online]. https://covid19testingtoolkit.centerforhealthsecurity.org/testing-trackers/antigen-and-molecular-tests-for-covid-19#lab.

[134] Phan T., Valeriano P., Boes S., McCullough M., Gribschaw J., Wells A. (2022). Evaluation of the ePlex Respiratory pathogen panel 2 to detect viral and bacterial pathogens, including SARS-CoV-2 Omicron in nasopharyngeal swabs. J. Clin. Virol. Plus.

[135] Sahajpal N.S., Mondal A.K., Ananth S., Njau A., Jones K., Ahluwalia P., Oza E., Ross T.M., Kota V., Kothandaraman A. (2022). Clinical validation of a multiplex PCR-based detection assay using saliva or nasopharyngeal samples for SARS-Cov-2, influenza A and B. Sci. Rep..

[136] Hashemi S.A., Safamanesh S., Ghasemzadeh-moghaddam H., Ghafouri M., Azimian A. (2021). High prevalence of SARS-CoV-2 and influenza A virus (H1N1) coinfection in dead patients in Northeastern Iran. J. Med. Virol..

[137] Fang Z., Zhang Y., Hang C., Ai J., Li S., Zhang W. (2020). Comparisons of viral shedding time of SARS-CoV-2 of different samples in ICU and non-ICU patients. J. Infect..

[138] Dong L., Zhou J., Niu C., Wang Q., Pan Y., Sheng S., Wang X., Zhang Y., Yang J., Liu M. (2021). Highly accurate and sensitive diagnostic detection of SARS-CoV-2 by digital PCR. Talanta.

[139] Suo T., Liu X., Feng J., Guo M., Hu W., Guo D., Ullah H., Yang Y., Zhang Q., Wang X. (2020). ddPCR: A more accurate tool for SARS-CoV-2 detection in low viral load specimens. Emerg. Microbes Infect..

[140] Dhar B.C. (2022). Diagnostic assay and technology advancement for detecting SARS-CoV-2 infections causing the COVID-19 pandemic. Anal. Bioanal. Chem..

[141] Shafie M.H., Antony Dass M., Ahmad Shaberi H.S., Zafarina Z. (2023). Screening and confirmation tests for SARS-CoV-2: Benefits and drawbacks. Beni Suef Univ. J. Basic Appl. Sci..

[142] Choi G., Moehling T.J., Meagher R.J. (2023). Advances in RT-LAMP for COVID-19 testing and diagnosis. Expert Rev. Mol. Diagn..

[143] Xu J., Ma Y., Song Z., Sun W., Liu Y., Shu C., Hua H., Yang M., Liang Q. (2023). Evaluation of an automated CRISPR-based diagnostic tool for rapid detection of COVID-19. Heliyon.

[144] New England Biolabs Loop-Mediated Isothermal Amplification [Online]. https://www.neb.com/en/applications/dna-amplification-pcr-and-qpcr/isothermal-amplification/loop-mediated-isothermal-amplification-lamp.

[145] Centers for Disease Control and Prevention (2022). Interim Guidelines for COVID-19 Antibody Testing in Clinical and Public Health Settings [Online]. https://www.cdc.gov/coronavirus/2019-ncov/hcp/testing/antibody-tests-guidelines.html.

[146] Food and Drug Administration (2023). Antibody (Serology) Testing for COVID-19: Information for Patients and Consumers [Online]. https://www.fda.gov/medical-devices/coronavirus-covid-19-and-medical-devices/antibody-serology-testing-covid-19-information-patients-and-consumers.

[147] Centers for Disease Control and Prevention (2023). Considerations for SARS-CoV-2 Antigen Testing for Healthcare Providers Testing Individuals in the Community [Online]. https://www.cdc.gov/coronavirus/2019-ncov/lab/resources/antigen-tests-guidelines.html.

[148] American Society for Microbiology (2020). How the SARS-CoV-2 EUA Antigen Tests Work [Online]. https://asm.org/articles/2020/august/how-the-sars-cov-2-eua-antigen-tests-work.

[149] Hashim I.A. (2024). Chapter 17—Analytical methods and special considerations. Tutorials in Clinical Chemistry.

[150] Food and Drug Administration (2024). At Home OTC COVID-19 Diagnostic Tests [Online]. https://www.fda.gov/medical-devices/coronavirus-covid-19-and-medical-devices/home-otc-covid-19-diagnostic-tests.

[151] Katzenschlager S., Bruemmer L.E., Schmitz S., Tolle H., Manten K., Gaeddert M., Erdmann C., Lindner A., Tobian F., Grilli M. (2023). Comparing SARS-CoV-2 antigen-detection rapid diagnostic tests for COVID-19 self-testing/self-sampling with molecular and professional-use tests: A systematic review and meta-analysis. Sci. Rep..

[152] Yadegari H., Mohammadi M., Maghsood F., Ghorbani A., Bahadori T., Golsaz-Shirazi F., Zarnani A.H., Salimi V., Jeddi-Tehrani M., Amiri M.M. (2023). Diagnostic performance of a novel antigen-capture ELISA for the detection of SARS-CoV-2. Anal. Biochem..

[153] Adnan N., Khandker S.S., Haq A., Chaity M.A., Khalek A., Nazim A.Q., Kaitsuka T., Tomizawa K., Mie M., Kobatake E. (2022). Detection of SARS-CoV-2 by antigen ELISA test is highly swayed by viral load and sample storage condition. Expert Rev. Anti-Infect. Ther..

[154] Weiss A., Jellingsø M., Sommer M.O.A. (2020). Spatial and temporal dynamics of SARS-CoV-2 in COVID-19 patients: A systematic review and meta-analysis. eBioMedicine.

[155] Xin H., Li Y., Wu P., Li Z., Lau E.H.Y., Qin Y., Wang L., Cowling B.J., Tsang T.K., Li Z. (2021). Estimating the Latent Period of Coronavirus Disease 2019 (COVID-19). Clin. Infect. Dis..

[156] Markov P.V., Ghafari M., Beer M., Lythgoe K., Simmonds P., Stilianakis N.I., Katzourakis A. (2023). The evolution of SARS-CoV-2. Nat. Rev. Microbiol..

[157] Hagag I.T., Pyrc K., Weber S., Balkema-Buschmann A., Groschup M.H., Keller M. (2022). Mutations in SARS-CoV-2 nucleocapsid in variants of concern impair the sensitivity of SARS-CoV-2 detection by rapid antigen tests. Front. Virol..

[158] Johnson B.A., Zhou Y., Lokugamage K.G., Vu M.N., Bopp N., Crocquet-Valdes P.A., Kalveram B., Schindewolf C., Liu Y., Scharton D. (2022). Nucleocapsid mutations in SARS-CoV-2 augment replication and pathogenesis. PLoS Pathog..

[159] El-Daly M.M. (2024). Advances and Challenges in SARS-CoV-2 Detection: A Review of Molecular and Serological Technologies. Diagnostics.

[160] Udugama B., Kadhiresan P., Kozlowski H.N., Malekjahani A., Osborne M., Li V.Y.C., Chen H., Mubareka S., Gubbay J.B., Chan W.C.W. (2020). Diagnosing COVID-19: The Disease and Tools for Detection. ACS Nano.

[161] Zhang Y.Z., Holmes E.C. (2020). A Genomic Perspective on the Origin and Emergence of SARS-CoV-2. Cell.

[162] World Health Organization Genomic Sequencing of SARS-CoV-2: A Guide to Implementation for Maximum Impact on Public Health. 8 January 2021. https://www.who.int/publications/i/item/9789240018440.

[163] ImmunoDiagnostics SARS-CoV-2 NP Ab ELISA Kit [CE-IVD] [Online]. https://www.immunodiagnostics.com.hk/product-page/sars-cov-2-np-ab-elisa-kit-ce-ivd.

[164] Centers for Disease Control and Prevention (2023). Understanding How COVID-19 Vaccines Work [Online]. https://www.cdc.gov/coronavirus/2019-ncov/vaccines/different-vaccines/how-they-work.html.

[165] Carabelli A.M., Peacock T.P., Thorne L.G., Harvey W.T., Hughes J., Peacock S.J., Barclay W.S., De Silva T.I., Towers G.J., Robertson D.L. (2023). SARS-CoV-2 variant biology: Immune escape, transmission and fitness. Nat. Rev. Microbiol..

[166] Papa Mze N., Kacel I., Beye M., Tola R., Sarr M., Basco L., Bogreau H., Colson P., Fournier P.E. (2023). High Throughput SARS-CoV-2 Genome Sequencing from 384 Respiratory Samples Using the Illumina COVIDSeq Protocol. Genes.

[167] Fox T., Geppert J., Dinnes J., Scandrett K., Bigio J., Sulis G., Hettiarachchi D., Mathangasinghe Y., Weeratunga P., Wickramasinghe D. (2022). Antibody tests for identification of current and past infection with SARS-CoV-2. Cochrane Database Syst. Rev..

[168] Kontou P.I., Braliou G.G., Dimou N.L., Nikolopoulos G., Bagos P.G. (2020). Antibody Tests in Detecting SARS-CoV-2 Infection: A Meta-Analysis. Diagnostics.

[169] Matinfar S., Mortezagholi S., Amiri D., Pashaiefar H., Eskandarian M., Ghadimi S., Nazari M.F., Tavakoli S., Valizadeh M., Namaki S. (2024). Investigating the Seroconversion Patterns of Specific Antibodies against Various Antigens of SARS-CoV-2 in Hospitalized COVID-19 Patients and Vaccinated Individuals. Arch. Clin. Infect. Dis..

[170] Imai K., Kitagawa Y., Tabata S., Kubota K., Nagura-Ikeda M., Matsuoka M., Miyoshi K., Sakai J., Ishibashi N., Tarumoto N. (2021). Antibody response patterns in COVID-19 patients with different levels of disease severity in Japan. J. Med. Virol..

[171] Nakano Y., Kurano M., Morita Y., Shimura T., Yokoyama R., Qian C., Xia F., He F., Kishi Y., Okada J. (2021). Time course of the sensitivity and specificity of anti-SARS-CoV-2 IgM and IgG antibodies for symptomatic COVID-19 in Japan. Sci. Rep..

[172] Long Q.X., Liu B.Z., Deng H.J., Wu G.C., Deng K., Chen Y.K., Liao P., Qiu J.F., Lin Y., Cai X.F. (2020). Antibody responses to SARS-CoV-2 in patients with COVID-19. Nat. Med..

[173] Bhattacharjee B., Rynjah D., Ahmed A.B., Newar A., Sengupta S., Chakrabarty S., Sahu R.K., Khan J. (2024). Overview of diagnostic tools and nano-based therapy of SARS-CoV-2 infection. Chem. Pap..

[174] Food and Drug Administration (2024). In Vitro Diagnostics Emergency Use Authorizations (EUAs)—Serology and Other Adaptive Immune Response Tests for SARS-CoV-2 [Online]. https://www.fda.gov/medical-devices/covid-19-emergency-use-authorizations-medical-devices/in-vitro-diagnostics-emergency-use-authorizations-euas-serology-and-other-adaptive-immune-response.

[175] Augustine R., Das S., Hasan A., Abdul Salam S., Augustine P., Dalvi Y.B., Varghese R., Primavera R., Yassine H.M., Thakor A.S. (2020). Rapid antibody-based COVID-19 mass surveillance: Relevance, challenges, and prospects in a pandemic and post-pandemic world. J. Clin. Med..

[176] Dimech W., Curley S., Subissi L., Ströher U., Perkins M.D., Cunningham J. (2023). Comprehensive, Comparative Evaluation of 35 Manual SARS-CoV-2 Serological Assays. Microbiol. Spectr..

[177] Dimech W., Curley S., Cai J.J. (2024). Comprehensive, comparative evaluation of 25 automated SARS-CoV-2 serology assays. Microbiol. Spectr..

[178] Li G., Hilgenfeld R., Whitley R., De Clercq E. (2023). Therapeutic strategies for COVID-19: Progress and lessons learned. Nat. Rev. Drug Discov..

[179] Eastman R.T., Roth J.S., Brimacombe K.R., Simeonov A., Shen M., Patnaik S., Hall M.D. (2020). Remdesivir: A Review of Its Discovery and Development Leading to Emergency Use Authorization for Treatment of COVID-19. ACS Central Sci..

[180] Barghash R.F., Fawzy I.M., Chandrasekar V., Singh A.V., Katha U., Mandour A.A. (2021). In Silico Modeling as a Perspective in Developing Potential Vaccine Candidates and Therapeutics for COVID-19. Coatings.

[181] Gottlieb R.L., Vaca C.E., Paredes R., Mera J., Webb B.J., Perez G., Oguchi G., Ryan P., Nielsen B.U., Brown M. (2021). Early Remdesivir to Prevent Progression to Severe Covid-19 in Outpatients. N. Engl. J. Med..

[182] Beigel J.H., Tomashek K.M., Dodd L.E., Mehta A.K., Zingman B.S., Kalil A.C., Hohmann E., Chu H.Y., Luetkemeyer A., Kline S. (2020). Remdesivir for the Treatment of COVID-19—Final Report. N. Engl. J. Med..

[183] Xie J., Wang Z. (2021). Can remdesivir and its parent nucleoside GS-441524 be potential oral drugs? An in vitro and in vivo DMPK assessment. Acta Pharm. Sin. B.

[184] Ader F., Bouscambert-Duchamp M., Hites M., Peiffer-Smadja N., Poissy J., Belhadi D., Diallo A., Lê M.-P., Peytavin G., Staub T. (2021). Remdesivir plus standard of care versus standard of care alone for the treatment of patients admitted to hospital with COVID-19 (DisCoVeRy): A phase 3, randomised, controlled, open-label trial. Lancet Infect. Dis..

[185] Pan H., Peto R., Restrepo A.M.H., Preziosi M.-P., Sathiyamoorthy V., Karim Q.A., Alejandria M., García C.H., Kieny M.-P., Malekzadeh R. (2022). Remdesivir and three other drugs for hospitalised patients with COVID-19: Final results of the WHO Solidarity randomised trial and updated meta-analyses. Lancet.

[186] Kalil A.C., Patterson T.F., Mehta A.K., Tomashek K.M., Wolfe C.R., Ghazaryan V., Marconi V.C., Ruiz-Palacios G.M., Hsieh L., Kline S. (2021). Baricitinib plus Remdesivir for Hospitalized Adults with COVID-19. N. Engl. J. Med..

[187] Kalil A.C., Mehta A.K., Patterson T.F., Erdmann N., Gomez C.A., Jain M.K., Wolfe C.R., Ruiz-Palacios G.M., Kline S., Pineda J.R. (2021). Efficacy of interferon beta-1a plus remdesivir compared with remdesivir alone in hospitalised adults with COVID-19: A double-blind, randomised, placebo-controlled, phase 3 trial. Lancet Respir. Med..

[188] Focosi D., Maggi F., McConnell S., Casadevall A. (2022). Very low levels of remdesivir resistance in SARS-CoV-2 genomes after 18 months of massive usage during the COVID19 pandemic: A GISAID exploratory analysis. Antivir. Res..

[189] Hedskog C., Rodriguez L., Roychoudhury P., Huang M.-L., Jerome K.R., Hao L., Ireton R.C., Li J., Perry J.K., Han D. (2023). Viral Resistance Analyses from the Remdesivir Phase 3 Adaptive COVID-19 Treatment Trial-1 (ACTT-1). J. Infect. Dis..

[190] Vangeel L., Chiu W., De Jonghe S., Maes P., Slechten B., Raymenants J., André E., Leyssen P., Neyts J., Jochmans D. (2022). Remdesivir, Molnupiravir and Nirmatrelvir remain active against SARS-CoV-2 Omicron and other variants of concern. Antivir. Res..

[191] Uraki R., Ito M., Kiso M., Yamayoshi S., Iwatsuki-Horimoto K., Sakai-Tagawa Y., Imai M., Koga M., Yamamoto S., Adachi E. (2023). Antiviral efficacy against and replicative fitness of an XBB.1.9.1 clinical isolate. iScience.

[192] Pitts J., Li J., Perry J.K., Du Pont V., Riola N., Rodriguez L., Lu X., Kurhade C., Xie X., Camus G. (2022). Remdesivir and GS-441524 Retain Antiviral Activity against Delta, Omicron, and Other Emergent SARS-CoV-2 Variants. Antimicrob. Agents Chemother..

[193] Cao Z., Gao W., Bao H., Feng H., Mei S., Chen P., Gao Y., Cui Z., Zhang Q., Meng X. (2023). VV116 versus Nirmatrelvir–Ritonavir for Oral Treatment of COVID-19. N. Engl. J. Med..

[194] Fan X., Dai X., Ling Y., Wu L., Tang L., Peng C., Huang C., Liu H., Lu H., Shen X. (2024). Oral VV116 versus placebo in patients with mild-to-moderate COVID-19 in China: A multicentre, double-blind, phase 3, randomised controlled study. Lancet Infect. Dis..

[195] Hashemian S.M., Sheida A., Taghizadieh M., Memar M.Y., Hamblin M.R., Baghi H.B., Nahand J.S., Asemi Z., Mirzaei H. (2023). Paxlovid (Nirmatrelvir/Ritonavir): A new approach to COVID-19 therapy?. Biomed. Pharmacother..

[196] Yan W., Zheng Y., Zeng X., He B., Cheng W. (2022). Structural biology of SARS-CoV-2: Open the door for novel therapies. Signal Transduct. Target. Ther..

[197] Zhang L., Lin D., Sun X., Curth U., Drosten C., Sauerhering L., Becker S., Rox K., Hilgenfeld R. (2020). Crystal structure of SARS-CoV-2 main protease provides a basis for design of improved α-ketoamide inhibitors. Science.

[198] Hammond J., Leister-Tebbe H., Gardner A., Abreu P., Bao W., Wisemandle W., Baniecki M., Hendrick V.M., Damle B., Simón-Campos A. (2022). Oral Nirmatrelvir for High-Risk, Nonhospitalized Adults with COVID-19. N. Engl. J. Med..

[199] Wong C.K.H., Lau J.J., Au I.C.H., Lau K.T.K., Hung I.F.N., Peiris M., Leung G.M., Wu J.T. (2023). Optimal timing of nirmatrelvir/ritonavir treatment after COVID-19 symptom onset or diagnosis: Target trial emulation. Nat. Commun..

[200] Arbel R., Sagy Y.W., Hoshen M., Battat E., Lavie G., Sergienko R., Friger M., Waxman J.G., Dagan N., Balicer R. (2022). Nirmatrelvir Use and Severe Covid-19 Outcomes during the Omicron Surge. N. Engl. J. Med..

[201] Li P., Wang Y., Lavrijsen M., Lamers M.M., de Vries A.C., Rottier R.J., Bruno M.J., Peppelenbosch M.P., Haagmans B.L., Pan Q. (2022). SARS-CoV-2 Omicron variant is highly sensitive to molnupiravir, nirmatrelvir, and the combination. Cell Res..

[202] Greasley S.E., Noell S., Plotnikova O., Ferre R., Liu W., Bolanos B., Fennell K., Nicki J., Craig T., Zhu Y. (2022). Structural basis for the in vitro efficacy of nirmatrelvir against SARS-CoV-2 variants. J. Biol. Chem..

[203] Takashita E., Kinoshita N., Yamayoshi S., Sakai-Tagawa Y., Fujisaki S., Ito M., Iwatsuki-Horimoto K., Halfmann P., Watanabe S., Maeda K. (2022). Efficacy of Antiviral Agents against the SARS-CoV-2 Omicron Subvariant BA.2. N. Engl. J. Med..

[204] Takashita E., Yamayoshi S., Simon V., van Bakel H., Sordillo E.M., Pekosz A., Fukushi S., Suzuki T., Maeda K., Halfmann P. (2022). Efficacy of Antibodies and Antiviral Drugs against Omicron BA.2.12.1, BA.4, and BA.5 Subvariants. N. Engl. J. Med..

[205] Saito A., Tamura T., Zahradnik J., Deguchi S., Tabata K., Anraku Y., Kimura I., Ito J., Yamasoba D., Nasser H. (2022). Virological characteristics of the SARS-CoV-2 Omicron BA.2.75 variant. Cell Host Microbe.

[206] Imai M., Ito M., Kiso M., Yamayoshi S., Uraki R., Fukushi S., Watanabe S., Suzuki T., Maeda K., Sakai-Tagawa Y. (2023). Efficacy of Antiviral Agents against Omicron Subvariants BQ.1.1 and XBB. N. Engl. J. Med..

[207] Aggarwal N.R., Molina K.C., Beaty L.E., Bennett T.D., Carlson N.E., Mayer D.A., Peers J.L., Russell S., Wynia M.K., Ginde A.A. (2023). Real-world use of nirmatrelvir–ritonavir in outpatients with COVID-19 during the era of omicron variants including BA.4 and BA.5 in Colorado, USA: A retrospective cohort study. Lancet Infect. Dis..

[208] Zhou X., Kelly S.P., Liang C., Li L., Shen R., Leister-Tebbe H.K., Terra S.G., Gaffney M., Russo L. (2022). Real-World Effectiveness of Nirmatrelvir/Ritonavir in Preventing Hospitalization among Patients with COVID-19 at High Risk for Severe Disease in the United States: A Nationwide Population-Based Cohort Study. medRxiv.

[209] Cegolon L., Pol R., Simonetti O., Filon F.L., Luzzati R. (2023). Molnupiravir, Nirmatrelvir/Ritonavir, or Sotrovimab for High-Risk COVID-19 Patients Infected by the Omicron Variant: Hospitalization, Mortality, and Time until Negative Swab Test in Real Life. Pharmaceuticals.

[210] Iketani S., Mohri H., Culbertson B., Hong S.J., Duan Y., Luck M.I., Annavajhala M.K., Guo Y., Sheng Z., Uhlemann A.-C. (2022). Multiple pathways for SARS-CoV-2 resistance to nirmatrelvir. Nature.

[211] Duan Y., Zhou H., Liu X., Iketani S., Lin M., Zhang X., Bian Q., Wang H., Sun H., Hong S.J. (2023). Molecular mechanisms of SARS-CoV-2 resistance to nirmatrelvir. Nature.

[212] Charness M.E., Gupta K., Stack G., Strymish J., Adams E., Lindy D.C., Mohri H., Ho D.D. (2022). Rebound of SARS-CoV-2 Infection after Nirmatrelvir–Ritonavir Treatment. N. Engl. J. Med..

[213] Wang L., Berger N.A., Davis P.B., Kaelber D.C., Volkow N.D., Xu R. (2022). COVID-19 rebound after Paxlovid and Molnupiravir during January–June 2022. MedRxiv.

[214] Anderson A.S., Caubel P., Rusnak J.M. (2022). Nirmatrelvir–Ritonavir and Viral Load Rebound in COVID-19. N. Engl. J. Med..

[215] Wong C.K.H., Lau K.T.K., Au I.C.H., Lau E.H.Y., Poon L.L.M., Hung I.F.N., Cowling B.J., Leung G.M. (2023). Viral burden rebound in hospitalised patients with COVID-19 receiving oral antivirals in Hong Kong: A population-wide retrospective cohort study. Lancet Infect. Dis..

[216] Wong G.L.-H., Yip T.C.-F., Lai M.S.-M., Wong V.W.-S., Hui D.S.-C., Lui G.C.-Y. (2022). Incidence of Viral Rebound after Treatment with Nirmatrelvir-Ritonavir and Molnupiravir. JAMA Netw. Open.

[217] Perelson A.S., Ribeiro R.M., Phan T. (2023). An explanation for SARS-CoV-2 rebound after Paxlovid treatment. medRxiv.

[218] Boucau J., Uddin R., Marino C., Regan J., Flynn J.P., Choudhary M.C., Chen G., Stuckwisch A.M., Mathews J., Liew M.Y. (2022). Characterization of Virologic Rebound Following Nirmatrelvir-Ritonavir Treatment for Coronavirus Disease 2019 (COVID-19). Clin. Infect. Dis..

[219] Carlin A.F., Clark A.E., Chaillon A., Garretson A.F., Bray W., Porrachia M., Santos A.T., Rana T.M., Smith D.M. (2022). Virologic and Immunologic Characterization of Coronavirus Disease 2019 Recrudescence After Nirmatrelvir/Ritonavir Treatment. Clin. Infect. Dis..

[220] Huang J., Zhou C., Deng J., Zhou J. (2022). JAK inhibition as a new treatment strategy for patients with COVID-19. Biochem. Pharmacol..

[221] Satarker S., Tom A.A., Shaji R.A., Alosious A., Luvis M., Nampoothiri M. (2020). JAK-STAT Pathway Inhibition and their Implications in COVID-19 Therapy. Postgrad. Med..

[222] Tanaka Y. (2023). A review of Janus kinase inhibitors for the treatment of COVID-19 pneumonia. Inflamm. Regen..

[223] Abani O., Abbas A., Abbas F., Abbas J., Abbas K., Abbas M., Abbasi S., Abbass H., Abbott A., Abbott A. (2022). Baricitinib in patients admitted to hospital with COVID-19 (RECOVERY): A randomised, controlled, open-label, platform trial and updated meta-analysis. Lancet.

[224] Marconi V.C., Ramanan A.V., de Bono S., Kartman C.E., Krishnan V., Liao R., Piruzeli M.L.B., Alatorre-Alexander J., Pellegrini R.d.C., Estrada V. (2021). Efficacy and safety of baricitinib for the treatment of hospitalised adults with COVID-19 (COV-BARRIER): A randomised, double-blind, parallel-group, placebo-controlled phase 3 trial. Lancet Respir. Med..

[225] Abizanda P., Mayo J.M.C., Romero M.M., Zamora E.B.C., Sahuquillo M.T.T., Rizos L.R., Sánchez-Jurado P.M., Sánchez-Nievas G., Escolano C.C., Serrano A.O. (2021). Baricitinib reduces 30-day mortality in older adults with moderate-to-severe COVID-19 pneumonia. J. Am. Geriatr. Soc..

[226] Stebbing J., Sánchez Nievas G., Falcone M., Youhanna S., Richardson P., Ottaviani S., Shen J.X., Sommerauer C., Tiseo G., Ghiadoni L. (2021). JAK inhibition reduces SARS-CoV-2 liver infectivity and modulates inflammatory responses to reduce morbidity and mortality. Sci. Adv..

[227] Wolfe C.R., Tomashek K.M., Patterson T.F., Gomez C.A., Marconi V.C., Jain M.K., Yang O.O., Paules C.I., Palacios G.M.R., Grossberg R. (2022). Baricitinib versus dexamethasone for adults hospitalised with COVID-19 (ACTT-4): A randomised, double-blind, double placebo-controlled trial. Lancet Respir. Med..

[228] Troseid M., Arribas J.R., Assoumou L., Holten A.R., Poissy J., Terzic V., Mazzaferri F., Rodriguez-Bano J., Eustace J., Hites M. (2023). Efficacy and safety of baricitinib in hospitalized adults with severe or critical COVID-19 (Bari-SolidAct): A randomised, double-blind, placebo-controlled phase 3 trial. Crit. Care.

[229] Ferro F., La Rocca G., Elefante E., Italiano N., Moretti M., Talarico R., Pelati E., Valentini K., Baldini C., Mozzo R. (2024). Baricitinib and Pulse Steroids Combination Treatment in Hyperinflammatory COVID-19: A Rheumatological Approach in the Intensive Care Unit. Int. J. Mol. Sci..

[230] Rezabakhsh A., Mojtahedi F., Tekantapeh S.T., Mahmoodpoor A., Ala A., Soleimanpour H. (2024). Therapeutic Impact of Tocilizumab in the Setting of Severe COVID-19; an Updated and Comprehensive Review on Current Evidence. Arch. Acad. Emerg. Med..

[231] Zhang C., Wu Z., Li J.-W., Zhao H., Wang G.Q. (2020). The cytokine release syndrome (CRS) of severe COVID-19 and Interleukin-6 receptor (IL-6R) antagonist Tocilizumab may be the key to reduce the mortality. Int. J. Antimicrob. Agents.

[232] Le R.Q., Li L., Yuan W., Shord S.S., Nie L., Habtemariam B.A., Przepiorka D., Farrell A.T., Pazdur R. (2018). FDA Approval Summary: Tocilizumab for Treatment of Chimeric Antigen Receptor T Cell-Induced Severe or Life-Threatening Cytokine Release Syndrome. Oncologist.

[233] Steuber T.D., Rosandich T., Cadwallader T., Steil L., Belk M., Yendrapalli U., Hassoun A., Edwards J. (2023). Dosing and Administration Strategies of Tocilizumab in Patients with COVID-19: A Retrospective Cohort Analysis. Ann. Pharmacother..

[234] Leung E., Crass R.L., Jorgensen S.C.J., Raybardhan S., Langford B.J., Moore W.J., Rhodes N.J. (2021). Pharmacokinetic/Pharmacodynamic Considerations of Alternate Dosing Strategies of Tocilizumab in COVID-19. Clin. Pharmacokinet..

[235] Tay M.Z., Poh C.M., Rénia L., MacAry P.A., Ng L.F.P. (2020). The trinity of COVID-19: Immunity, inflammation and intervention. Nat. Rev. Immunol..

[236] RECOVERY Collaborative Group (2021). Tocilizumab in patients admitted to hospital with COVID-19 (RECOVERY): A randomised, controlled, open-label, platform trial. Lancet.

[237] Gordon A.C., Mouncey P.R., Al-Beidh F., Rowan K.M., Nichol A.D., Arabi Y.M., Annane D., Beane A., van Bentum-Puijk W., Berry L.R. (2021). Interleukin-6 Receptor Antagonists in Critically Ill Patients with COVID-19. N. Engl. J. Med..

[238] Alkhateeb T., Stollings J.L., Sohn I., Liu D., Fleenor L.M., Ely E.W., Lahiri S. (2024). Tocilizumab is associated with reduced delirium and coma in critically ill patients with COVID-19. Sci. Rep..

[239] Rashid M.H., Sparrow N.A., Anwar F., Guidry G., Covarrubias A.E., Pang H., Bogguri C., Karumanchi S.A., Lahiri S. (2021). Interleukin-6 mediates delirium-like phenotypes in a murine model of urinary tract infection. J. Neuroinflamm..

[240] Anwar F., Sparrow N.A., Rashid M.H., Guidry G., Gezalian M.M., Ley E.J., Koronyo-Hamaoui M., Danovitch I., Ely E.W., Karumanchi S.A. (2022). Systemic interleukin-6 inhibition ameliorates acute neuropsychiatric phenotypes in a murine model of acute lung injury. Crit. Care.

[241] Sparrow N.A., Anwar F., Covarrubias A.E., Rajput P.S., Rashid M.H., Nisson P.L., Gezalian M.M., Toossi S., Ayodele M.O., Karumanchi S.A. (2021). IL-6 Inhibition Reduces Neuronal Injury in a Murine Model of Ventilator-induced Lung Injury. Am. J. Respir. Cell Mol. Biol..

[242] Knorr J.P., Colomy V., Mauriello C.M., Ha S. (2020). Tocilizumab in patients with severe COVID-19: A single-center observational analysis. J. Med. Virol..

[243] Hager D.N., Dinglas V.D., Subhas S., Rowden A.M., Neufeld K.J., Bienvenu O.J., Touradji P., Colantuoni E., Reddy D.R., Brower R.G. (2013). Reducing Deep Sedation and Delirium in Acute Lung Injury Patients. Crit. Care Med..

[244] Chan K.H., Patel B., Podel B., Szablea M.E., Shaaban H.S., Guron G., Slim J. (2021). Tocilizumab and Thromboembolism in COVID-19: A Retrospective Hospital-Based Cohort Analysis. Cureus.

[245] Hafez W., Ziade M.A., Arya A., Saleh H., Abdelshakor M., Alla O.F., Agrawal P., Ali S., Rao S.R., Gupta S. (2022). Treatment Outcomes of Tocilizumab in Critically-Ill COVID-19 Patients, Single-Centre Retrospective Study. Antibiotics.

[246] Rosas I.O., Bräu N., Waters M., Go R.C., Hunter B.D., Bhagani S., Skiest D., Aziz M.S., Cooper N., Douglas I.S. (2021). Tocilizumab in Hospitalized Patients with Severe Covid-19 Pneumonia. N. Engl. J. Med..

[247] Aljuhani O., Al Sulaiman K., Korayem G.B., Altebainawi A.F., Alsohimi S., Alqahtani R., Alfaifi S., Alharbi A., AlKhayrat A., Hattan A. (2024). The association between tocilizumab therapy and the development of thrombosis in critically ill patients with COVID-19: A multicenter, cohort study. Sci. Rep..

[248] Guaraldi G., Meschiari M., Cozzi-Lepri A., Milic J., Tonelli R., Menozzi M., Franceschini E., Cuomo G., Orlando G., Borghi V. (2020). Tocilizumab in patients with severe COVID-19: A retrospective cohort study. Lancet Rheumatol..

[249] Hermine O., Mariette X., Tharaux P.-L., Resche-Rigon M., Porcher R., Ravaud P., Bureau S., Dougados M., Tibi A., CORIMUNO-19 Collaborative Group (2021). Effect of Tocilizumab vs Usual Care in Adults Hospitalized with COVID-19 and Moderate or Severe Pneumonia: A Randomized Clinical Trial. JAMA Intern. Med..

[250] Stone J.H., Frigault M.J., Serling-Boyd N.J., Fernandes A.D., Harvey L., Foulkes A.S., Horick N.K., Healy B.C., Shah R., Bensaci A.M. (2020). Efficacy of Tocilizumab in Patients Hospitalized with COVID-19. N. Engl. J. Med..

[251] Lu D.-E., Ou T.-Y., Kang J.-W., Ong J.Y., Chen I.-J., Lee C.-H., Lee M.-C. (2024). The association between tocilizumab and the secondary bloodstream infection maybe nonsignificant in hospitalized patients with SARS-CoV-2 infection: A cohort study. J. Microbiol. Immunol. Infect..

[252] Wang Y., Liang Q., Chen F., Zheng J., Chen Y., Chen Z., Li R., Li X. (2023). Immune-Cell-Based Therapy for COVID-19: Current Status. Viruses.

[253] Kállay K., Kassa C., Réti M., Karászi É., Sinkó J., Goda V., Stréhn A., Csordás K., Horváth O., Szederjesi A. (2018). Early Experience with CliniMACS Prodigy CCS (IFN-gamma) System in Selection of Virus-specific T Cells from Third-party Donors for Pediatric Patients with Severe Viral Infections after Hematopoietic Stem Cell Transplantation. J. Immunother..

[254] Naik S., Nicholas S.K., Martinez C.A., Leen A.M., Hanley P.J., Gottschalk S.M., Rooney C.M., Hanson I.C., Krance R.A., Shpall E.J. (2016). Adoptive immunotherapy for primary immunodeficiency disorders with virus-specific T lymphocytes. J. Allergy Clin. Immunol..

[255] Chu Y., Milner J., Lamb M., Maryamchik E., Rigot O., Ayello J., Harrison L., Shaw R., Behbehani G.K., Mardis E.R. (2022). Manufacture and Characterization of Good Manufacturing Practice-Compliant SARS-CoV-2 Cytotoxic T Lymphocytes. J. Infect. Dis..

[256] Bleakley M.M., Gooley T.A., Hilzinger B., Riddell S.R., Shlomchik W.D. (2016). NaïVe T Cell Depletion of PBSC Grafts Results in Very Low Rates of Chronic Gvhd and High Survival. Blood.

[257] Dan J.M., Mateus J., Kato Y., Hastie K.M., Yu E.D., Faliti C.E., Grifoni A., Ramirez S.I., Haupt S., Frazier A. (2021). Immunological Memory to SARS-CoV-2 Assessed for Up to 8 Months after Infection. Science.

[258] Papadopoulou A., Karavalakis G., Papadopoulou E., Xochelli A., Bousiou Z., Vogiatzoglou A., Papayanni P.G., Georgakopoulou A., Giannaki M., Stavridou F. (2023). SARS-CoV-2-specific T cell therapy for severe COVID-19: A randomized phase 1/2 trial. Nat. Med..

[259] Ferreras C., Hernández-Blanco C., Martín-Quirós A., Al-Akioui-Sanz K., Mora-Rillo M., Ibáñez F., Díaz-Almirón M., Cano-Ochando J., Lozano-Ojalvo D., Jiménez-González M. (2024). Results of phase 2 randomized multi-center study to evaluate the safety and efficacy of infusion of memory T cells as adoptive therapy in severe acute respiratory syndrome coronavirus 2 (SARS-CoV-2) pneumonia and/or lymphopenia (RELEASE NCT04578210). Cytotherapy.

[260] Ong R.Y.L., Seah V.X.F., Chong C.Y., Thoon K.C., Tan N.W.H., Li J., Nadua K.D., Soh S.Y., Seng M.S.-F., Pham T.N.A. (2023). A cohort study of COVID-19 infection in pediatric oncology patients plus the utility and safety of remdesivir treatment. Acta Oncol..

[261] Tarke A., Coelho C.H., Zhang Z., Dan J.M., Yu E.D., Methot N., Bloom N.I., Goodwin B., Phillips E., Mallal S. (2022). SARS-CoV-2 vaccination induces immunological T cell memory able to cross-recognize variants from Alpha to Omicron. Cell.

[262] Zavvar M., Yahyapoor A., Baghdadi H., Zargaran S., Assadiasl S., Abdolmohammadi K., Abooei A.H., Sattarian M.R., JalaliFarahani M., Zarei N. (2022). COVID-19 immunotherapy: Treatment based on the immune cell-mediated approaches. Int. Immunopharmacol..

[263] Xu Z., Jiang X., Dai X., Li B. (2022). The Dynamic Role of FOXP3+ Tregs and Their Potential Therapeutic Applications during SARS-CoV-2 Infection. Front. Immunol..

[264] Fransson M., Piras E., Burman J., Nilsson B., Essand M., Lu B., Harris R.A., Magnusson P.U., Brittebo E., Loskog A.S. (2012). CAR/FoxP3-engineered T regulatory cells target the CNS and suppress EAE upon intranasal delivery. J. Neuroinflamm..

[265] Blat D., Zigmond E., Alteber Z., Waks T., Eshhar Z. (2014). Suppression of Murine Colitis and its Associated Cancer by Carcinoembryonic Antigen-Specific Regulatory T Cells. Mol. Ther..

[266] Skuljec J., Chmielewski M., Happle C., Habener A., Busse M., Abken H., Hansen G. (2017). Chimeric Antigen Receptor-Redirected Regulatory T Cells Suppress Experimental Allergic Airway Inflammation, a Model of Asthma. Front. Immunol..

[267] Guan T., Zhou X., Zhou W., Lin H. (2023). Regulatory T cell and macrophage crosstalk in acute lung injury: Future perspectives. Cell Death Discov..

[268] Wu D., Yang X.O. (2020). TH17 responses in cytokine storm of COVID-19: An emerging target of JAK2 inhibitor Fedratinib. J. Microbiol. Immunol. Infect..

[269] Gonçalves-Pereira M.H., Santiago L., Ravetti C.G., Vassallo P.F., de Andrade M.V.M., Vieira M.S., Oliveira F.d.F.S.d., Carobin N.V., Li G., Sabino A.d.P. (2022). Dysfunctional phenotype of systemic and pulmonary regulatory T cells associate with lethal COVID-19 cases. Immunology.

[270] Khesht A.M., Karpisheh V., Saeed B.Q., Zekiy A.O., Yapanto L.M., Afjadi M.N., Aksoun M., Esfahani M.N., Aghakhani F., Movahed M. (2021). Different T cell related immunological profiles in COVID-19 patients compared to healthy controls. Int. Immunopharmacol..

[271] Gladstone D.E., D’Alessio F.R., Howard C., Lyu M.-A., Mock J.R., Gibbs K.W., Abrams D., Huang M., Zeng K., Herlihy J.P. (2023). Randomized, double-blinded, placebo-controlled trial of allogeneic cord blood T-regulatory cells for treatment of COVID-19 ARDS. Blood Adv..

[272] Buitrago-Molina L.E., Pietrek J., Noyan F., Schlue J., Manns M.P., Wedemeyer H., Hardtke-Wolenski M., Jaeckel E. (2020). Treg-specific IL-2 therapy can reestablish intrahepatic immune regulation in autoimmune hepatitis. J. Autoimmun..

[273] Humrich J.Y., Riemekasten G. (2016). Restoring regulation—IL-2 therapy in systemic lupus erythematosus. Expert Rev. Clin. Immunol..

[274] Boyman O., Sprent J. (2012). The role of interleukin-2 during homeostasis and activation of the immune system. Nat. Rev. Immunol..

[275] Dhawan M., Rabaan A.A., Alwarthan S., Alhajri M., Halwani M.A., Alshengeti A., Najim M.A., Alwashmi A.S.S., Alshehri A.A., Alshamrani S.A. (2023). Regulatory T Cells (Tregs) and COVID-19: Unveiling the Mechanisms, and Therapeutic Potentialities with a Special Focus on Long COVID. Vaccines.

[276] Alavi-Dana S.M.M., Gholami Y., Meghdadi M., Fadaei M.S., Askari V.R. (2023). Mesenchymal stem cell therapy for COVID-19 infection. Inflammopharmacology.

[277] Cipriani P., Carubbi F., Liakouli V., Marrelli A., Perricone C., Perricone R., Alesse E., Giacomelli R. (2013). Stem cells in autoimmune diseases: Implications for pathogenesis and future trends in therapy. Autoimmun. Rev..

[278] Peng Y., Chen X., Liu Q., Zhang X., Huang K., Liu L., Li H., Zhou M., Huang F., Fan Z. (2014). Mesenchymal stromal cells infusions improve refractory chronic graft versus host disease through an increase of CD5+ regulatory B cells producing interleukin 10. Leukemia.

[279] Xu J.-Y., Lee Y.-K., Ran X., Liao S.-Y., Yang J., Au K.-W., Lai W.-H., Esteban M.A., Tse H.-F. (2016). Generation of Induced Cardiospheres via Reprogramming of Skin Fibroblasts for Myocardial Regeneration. Stem Cells.

[280] Saleh M., Vaezi A.A., Aliannejad R., Sohrabpour A.A., Kiaei S.Z.F., Shadnoush M., Siavashi V., Aghaghazvini L., Khoundabi B., Abdoli S. (2021). Cell therapy in patients with COVID-19 using Wharton’s jelly mesenchymal stem cells: A phase 1 clinical trial. Stem Cell Res. Ther..

[281] Sengupta V., Sengupta S., Lazo A., Woods P., Nolan A., Bremer N. (2020). Exosomes Derived from Bone Marrow Mesenchymal Stem Cells as Treatment for Severe COVID-19. Stem Cells Dev..

[282] Adas G., Cukurova Z., Yasar K.K., Yilmaz R., Isiksacan N., Kasapoglu P., Yesilbag Z., Koyuncu I., Karaoz E. (2021). The Systematic Effect of Mesenchymal Stem Cell Therapy in Critical COVID-19 Patients: A Prospective Double Controlled Trial. Cell Transplant..

[283] Dilogo I.H., Aditianingsih D., Sugiarto A., Burhan E., Damayanti T., Sitompul P.A., Mariana N., Antarianto R.D., Liem I.K., Kispa T. (2021). Umbilical Cord Mesenchymal Stromal Cells as Critical COVID-19 Adjuvant Therapy: A Randomized Controlled Trial. Stem Cells Transl. Med..

[284] Ringden O., Roshandel E., Pirsalehi A., Kazemi S., Sankanian G., Majidi M., Salimi M., Aghdami N., Sadrosadat H., Kochaksaraei S.S. (2022). Conquering the cytokine storm in COVID-19-induced ARDS using placenta-derived decidua stromal cells. Biol. Blood Marrow Transplant..

[285] Farkhad N.K., Sedaghat A., Reihani H., Moghadam A.A., Moghadam A.B., Ghaebi N.K., Khodadoust M.A., Ganjali R., Tafreshian A.R., Tavakol-Afshari J. (2022). Mesenchymal stromal cell therapy for COVID-19-induced ARDS patients: A successful phase 1, control-placebo group, clinical trial. Stem Cell Res. Ther..

[286] Monsel A., Hauw-Berlemont C., Mebarki M., Heming N., Mayaux J., Tchoumba O.N., Diehl J.-L., Demoule A., Annane D., Marois C. (2022). Treatment of COVID-19-associated ARDS with mesenchymal stromal cells: A multicenter randomized double-blind trial. Crit. Care.

[287] Shi L., Zheng Y., Cheng Z., Ji N., Niu C., Wang Y., Huang T., Li R., Huang M., Chen X. (2022). One-year follow-up study after patients with severe COVID-19 received human umbilical cord mesenchymal stem cells treatment. Stem Cell Res. Ther..

[288] Li T.-T., Li T.-T., Zhang B., Zhang B., Fang H., Fang H., Shi M., Shi M., Yao W.-Q., Yao W.-Q. (2023). Human mesenchymal stem cell therapy in severe COVID-19 patients: 2-year follow-up results of a randomized, double-blind, placebo-controlled trial. eBioMedicine.

[289] Yudintceva N., Mikhailova N., Fedorov V., Samochernych K., Vinogradova T., Muraviov A., Shevtsov M. (2022). Mesenchymal Stem Cells and MSCs-Derived Extracellular Vesicles in Infectious Diseases: From Basic Research to Clinical Practice. Bioengineering.

[290] Lukomska B., Stanaszek L., Zuba-Surma E., Legosz P., Sarzynska S., Drela K. (2019). Challenges and Controversies in Human Mesenchymal Stem Cell Therapy. Stem Cells Int..

[291] Polack F.P., Thomas S.J., Kitchin N., Absalon J., Gurtman A., Lockhart S., Perez J.L., Pérez Marc G., Moreira E.D., Zerbini C. (2020). Safety and efficacy of the BNT162b2 mRNA COVID-19 vaccine. N. Engl. J. Med..

[292] Dunkle L.M., Kotloff K.L., Gay C.L., Áñez G., Adelglass J.M., Barrat Hernández A.Q., Harper W.L., Duncanson D.M., McArthur M.A., Florescu D.F. (2022). Efficacy and safety of NVX-CoV2373 in adults in the United States and Mexico. N. Engl. J. Med..

[293] Gilbert P.B., Montefiori D.C., McDermott A.B., Fong Y., Benkeser D., Deng W., Zhou H., Houchens C.R., Martins K., Jayashankar L. (2022). Immune correlates analysis of the mRNA-1273 COVID-19 vaccine efficacy clinical trial. Science.

[294] Collier A.-R.Y., Yu J., McMahan K., Liu J., Chandrashekar A., Maron J.S., Atyeo C., Martinez D.R., Ansel J.L., Aguayo R. (2021). Differential kinetics of immune responses elicited by COVID-19 vaccines. N. Engl. J. Med..

[295] Yang S., Yu Y., Xu Y., Jian F., Song W., Yisimayi A., Wang P., Wang J., Liu J., Yu L. (2024). Fast evolution of SARS-CoV-2 BA. 2.86 to JN. 1 under heavy immune pressure. Lancet Infect. Dis..

[296] Wang Q., Guo Y., Bowen A., Mellis I.A., Valdez R., Gherasim C., Gordon A., Liu L., Ho D.D. (2024). XBB.1.5 monovalent mRNA vaccine booster elicits robust neutralizing antibodies against XBB subvariants and JN.1. Cell Host Microbe.

[297] Kojima N., Adams K., Self W.H., Gaglani M., McNeal T., Ghamande S., Steingrub J.S., Shapiro N.I., Duggal A., Busse L.W. (2023). Changing severity and epidemiology of adults hospitalized with coronavirus disease 2019 (COVID-19) in the United States after introduction of COVID-19 vaccines, March 2021–August 2022. Clin. Infect. Dis..

[298] Tenforde M.W., Self W.H., Adams K., Gaglani M., Ginde A.A., McNeal T., Ghamande S., Douin D.J., Talbot H.K., Casey J.D. (2021). Association between mRNA vaccination and COVID-19 hospitalization and disease severity. JAMA.

[299] Fitzpatrick M.C., Galvani A.P. (2021). Optimizing age-specific vaccination. Science.

[300] Zaoui S., Foguem C., Tchuente D., Fosso-Wamba S., Kamsu-Foguem B. (2023). The viability of supply chains with interpretable learning systems: The case of COVID-19 vaccine deliveries. Glob. J. Flex. Syst. Manag..

[301] Gavi, the Vaccine Alliance (2023). COVAX Explained. [Online]. Gavi, the Vaccine Alliance. https://www.gavi.org/vaccineswork/covax-explained.

[302] World Health Organization (2023). COVID-19 Vaccinations Shift to Regular Immunization as COVAX Draws to a Close. [Online]. World Health Organization. https://www.who.int/news/item/19-12-2023-covid-19-vaccinations-shift-to-regular-immunization-as-covax-draws-to-a-close.

[303] Sign¢ L. (2021). Strategies for Effective Health Care for Africa in the Fourth Industrial Revolution, Bridging the Gap between the Promise and Delivery. [Online]. https://www.brookings.edu/wp-content/uploads/2021/10/Strategies-for-effective-health-care-delivery-in-Africa_FINAL.pdf.

[304] Hierink F., Okiro E.A., Flahault A., Ray N. (2021). The winding road to health: A systematic scoping review on the effect of geographical accessibility to health care on infectious diseases in low- and middle-income countries. PLoS ONE.

[305] Med Aditus (2023). Rwanda Welcomes Africa’s First Mobile Vaccine-Production Units. Med Aditus. [Online]. https://medaditus.org/news-articles/rwanda-welcomes-africas-first-mobile-vaccine-production-units/.

[306] Masresha B., Ruiz M.A.S., Atuhebwe P., Mihigo R. (2022). The first year of COVID-19 vaccine roll-out in Africa: Challenges and lessons learned. Pan Afr. Med. J..

[307] Vogel A.B., Kanevsky I., Che Y., Swanson K.A., Muik A., Vormehr M., Kranz L.M., Walzer K.C., Hein S., Güler A. (2021). BNT162b vaccines protect rhesus macaques from SARS-CoV-2. Nature.

[308] Muik A., Lui B.G., Wallisch A.-K., Bacher M., Mühl J., Reinholz J., Ozhelvaci O., Beckmann N., Garcia R.d.l.C.G., Poran A. (2022). Neutralization of SARS-CoV-2 Omicron by BNT162b2 mRNA vaccine–elicited human sera. Science.

[309] Walsh E.E., Frenck R.W., Falsey A.R., Kitchin N., Absalon J., Gurtman A., Lockhart S., Neuzil K., Mulligan M.J., Bailey R. (2020). Safety and Immunogenicity of Two RNA-Based COVID-19 Vaccine Candidates. N. Engl. J. Med..

[310] Sahin U., Muik A., Derhovanessian E., Vogler I., Kranz L.M., Vormehr M., Baum A., Pascal K., Quandt J., Maurus D. (2020). COVID-19 vaccine BNT162b1 elicits human antibody and TH1 T-cell responses. Nature.

[311] Thomas S.J., Moreira E.D., Kitchin N., Absalon J., Gurtman A., Lockhart S., Perez J.L., Pérez Marc G., Polack F.P., Zerbini C. (2021). Safety and Efficacy of the BNT162b2 mRNA Covid-19 Vaccine through 6 Months. N. Engl. J. Med..

[312] Chemaitelly H., Tang P., Hasan M.R., AlMukdad S., Yassine H.M., Benslimane F.M., Al Khatib H.A., Coyle P., Ayoub H.H., Al Kanaani Z. (2021). Waning of BNT162b2 Vaccine Protection against SARS-CoV-2 Infection in Qatar. N. Engl. J. Med..

[313] Tartof S.Y., Slezak J.M., Fischer H., Hong V., Ackerson B.K., Ranasinghe O.N., Frankland T.B., Ogun O.A., Zamparo J.M., Gray S. (2021). Effectiveness of mRNA BNT162b2 COVID-19 vaccine up to 6 months in a large integrated health system in the USA: A retrospective cohort study. Lancet.

[314] Tang P., Hasan M.R., Chemaitelly H., Yassine H.M., Benslimane F.M., Al Khatib H.A., AlMukdad S., Coyle P., Ayoub H.H., Al Kanaani Z. (2021). BNT162b2 and mRNA-1273 COVID-19 vaccine effectiveness against the SARS-CoV-2 Delta variant in Qatar. Nat. Med..

[315] Abu-Raddad L.J., Chemaitelly H., Butt A.A. (2021). Effectiveness of the BNT162b2 COVID-19 Vaccine against the B.1.1.7 and B.1.351 Variants. N. Engl. J. Med..

[316] Abu-Raddad L.J., Chemaitelly H., Ayoub H.H., AlMukdad S., Yassine H.M., Al-Khatib H.A., Smatti M.K., Tang P., Hasan M.R., Coyle P. (2022). Effect of mRNA Vaccine Boosters against SARS-CoV-2 Omicron Infection in Qatar. N. Engl. J. Med..

[317] Veneti L., Berild J.D., Watle S.V., Starrfelt J., Greve-Isdahl M., Langlete P., Bøås H., Bragstad K., Hungnes O., Meijerink H. (2023). Effectiveness of BNT162b2 vaccine against SARS-CoV-2 Delta and Omicron infection in adolescents, Norway, August 2021 to January 2022. Int. J. Infect. Dis..

[318] Chatzilena A., Hyams C., Challen R., Marlow R., King J., Adegbite D., Kinney J., Clout M., Maskell N., Oliver J. (2022). Effectiveness of BNT162b2 COVID-19 vaccination in prevention of hospitalisations and severe disease in adults with SARS-CoV-2 Delta (B.1.617.2) and Omicron (B.1.1.529) variant between June 2021 and July 2022: A prospective test negative case–control study. Lancet Reg. Health—Eur..

[319] Moreira E.D., Kitchin N., Xu X., Dychter S.S., Lockhart S., Gurtman A., Perez J.L., Zerbini C., Dever M.E., Jennings T.W. (2022). Safety and Efficacy of a Third Dose of BNT162b2 COVID-19 Vaccine. N. Engl. J. Med..

[320] Pather S., Muik A., Rizzi R., Mensa F. (2023). Clinical development of variant-adapted BNT162b2 COVID-19 vaccines: The early Omicron era. Expert Rev. Vaccines.

[321] US Centers for Disease Control and Prevention (2024). Stay Up to Date with COVID-19 Vaccines. https://www.cdc.gov/coronavirus/2019-ncov/vaccines/stay-up-to-date.html#UTD.

[322] Winokur P., Gayed J., Fitz-Patrick D., Thomas S.J., Diya O., Lockhart S., Xu X., Zhang Y., Bangad V., Schwartz H.I. (2023). Bivalent Omicron BA.1–Adapted BNT162b2 Booster in Adults Older than 55 Years. N. Engl. J. Med..

[323] Muik A., Lui B.G., Bacher M., Wallisch A.-K., Toker A., Couto C.I.C., Güler A., Mampilli V., Schmitt G.J., Mottl J. (2022). Exposure to BA.4/5 S protein drives neutralization of Omicron BA.1, BA.2, BA.2.12.1, and BA.4/5 in vaccine-experienced humans and mice. Sci. Immunol..

[324] Muik A., Lui B.G., Bacher M., Wallisch A.-K., Toker A., Finlayson A., Krüger K., Ozhelvaci O., Grikscheit K., Hoehl S. (2022). Omicron BA.2 breakthrough infection enhances cross-neutralization of BA.2.12.1 and BA.4/BA.5. Sci. Immunol..

[325] Zou J., Kurhade C., Patel S., Kitchin N., Tompkins K., Cutler M., Cooper D., Yang Q., Cai H., Muik A. (2023). Neutralization of BA.4–BA.5, BA.4.6, BA.2.75.2, BQ.1.1, and XBB.1 with Bivalent Vaccine. N. Engl. J. Med..

[326] Andersson N.W., Thiesson E.M., Baum U., Pihlström N., Starrfelt J., Faksová K., Poukka E., Meijerink H., Ljung R., Hviid A. (2023). Comparative effectiveness of bivalent BA.4-5 and BA.1 mRNA booster vaccines among adults aged ≥ 50 years in Nordic countries: Nationwide cohort study. BMJ.

[327] Tartof S.Y., Slezak J.M., Puzniak L., Hong V., Frankland T.B., Ackerson B.K., Xie F., Takhar H., Ogun O.A., Simmons S. (2023). Effectiveness of BNT162b2 BA.4/5 bivalent mRNA vaccine against a range of COVID-19 outcomes in a large health system in the USA: A test-negative case–control study. Lancet Respir. Med..

[328] Kurhade C., Zou J., Xia H., Liu M., Chang H.C., Ren P., Xie X., Shi P. (2022). Low neutralization of SARS-CoV-2 Omicron BA.2.75.2, BQ.1.1 and XBB.1 by parental mRNA vaccine or a BA.5 bivalent booster. Nat. Med..

[329] Gayed J., Bangad V., Xu X., Mensa F., Cutler M., Türeci Ö., Şahin U., Modjarrad K., Swanson K.A., Anderson A.S. (2024). Immunogenicity of the Monovalent Omicron XBB.1.5-Adapted BNT162b2 COVID-19 Vaccine against XBB.1.5, BA.2.86, and JN.1 Sublineages: A Phase 2/3 Trial. Vaccines.

[330] Gayed J., Diya O., Lowry F.S., Xu X., Bangad V., Mensa F., Zou J., Xie X., Hu Y., Lu C. (2024). Safety and Immunogenicity of the Monovalent Omicron XBB.1.5-Adapted BNT162b2 COVID-19 Vaccine in Individuals ≥12 Years Old: A Phase 2/3 Trial. Vaccines.

[331] Hansen C.H., Hansen C.H., Moustsen-Helms I.R., Moustsen-Helms I.R., Rasmussen M., Rasmussen M., Søborg B., Søborg B., Ullum H., Ullum H. (2024). Short-term effectiveness of the XBB.1.5 updated COVID-19 vaccine against hospitalisation in Denmark: A national cohort study. Lancet Infect. Dis..

[332] van Werkhoven C.H., Valk A.-W., Smagge B., de Melker H.E., Knol M.J., Hahné S.J., Hof S.v.D., de Gier B. (2024). Early COVID-19 vaccine effectiveness of XBB.1.5 vaccine against hospitalisation and admission to intensive care, the Netherlands, 9 October to 5 December 2023. Eurosurveillance.

[333] Shrestha N.K., Burke P.C., Nowacki A.S., Gordon S.M. (2024). Effectiveness of the 2023–2024 Formulation of the Coronavirus Disease 2019 Messenger RNA Vaccine. Clin. Infect. Dis..

[334] Link-Gelles R., Ciesla A.A., Mak J., Miller J.D., Silk B.J., Lambrou A.S., Paden C.R., Shirk P., Britton A., Smith Z.R. (2024). Early Estimates of Updated 2023–2024 (Monovalent XBB.1.5) COVID-19 Vaccine Effectiveness Against Symptomatic SARS-CoV-2 Infection Attributable to Co-Circulating Omicron Variants Among Immunocompetent Adults—Increasing Community Access to Testing Program, United States, September 2023–January 2024. MMWR. Morb. Mortal. Wkly. Rep..

[335] Witberg G., Barda N., Hoss S., Richter I., Wiessman M., Aviv Y., Grinberg T., Auster O., Dagan N., Balicer R.D. (2021). Myocarditis after Covid-19 Vaccination in a Large Health Care Organization. N. Engl. J. Med..

[336] Comirnaty COVID-19 Vaccine (2022). [Online] Summary of Product Characteristics. European Medicines Agency. https://www.ema.europa.eu/en/documents/product-information/comirnaty-epar-product-information_en.pdf.

[337] Goddard K., Lewis N., Fireman B., Weintraub E., Shimabukuro T., Zerbo O., Boyce T.G., Oster M.E., Hanson K.E., Donahue J.G. (2022). Risk of myocarditis and pericarditis following BNT162b2 and mRNA-1273 COVID-19 vaccination. Vaccine.

[338] Husby A., Gulseth H.L., Hovi P., Hansen J.V., Pihlström N., Gunnes N., Härkänen T., Dahl J., Karlstad Ø., Heliö T. (2023). Clinical outcomes of myocarditis after SARS-CoV-2 mRNA vaccination in four Nordic countries: Population based cohort study. BMJ Med..

[339] Fronza M., Thavendiranathan P., Chan V., Karur G.R., Udell J.A., Wald R.M., Hong R., Hanneman K. (2022). Myocardial Injury Pattern at MRI in COVID-19 Vaccine–Associated Myocarditis. Radiology.

[340] Centers for Disease Control and Prevention (2023). [Online] Selected Adverse Events Reported after COVID-19 Vaccination. https://www.cdc.gov/coronavirus/2019-ncov/vaccines/safety/adverse-events.html.

[341] Smadja D.M., Yue Q.-Y., Chocron R., Sanchez O., Louet A.L.-L. (2021). Vaccination against COVID-19: Insight from arterial and venous thrombosis occurrence using data from VigiBase. Eur. Respir. J..

[342] Elalamy I., Gerotziafas G., Alamowitch S., Laroche J.-P., Van Dreden P., Ageno W., Beyer-Westendorf J., Cohen A.T., Jimenez D., Brenner B. (2021). SARS-CoV-2 Vaccine and Thrombosis: An Expert Consensus on Vaccine-Induced Immune Thrombotic Thrombocytopenia. Thromb. Haemost..

[343] Fan B.E., Shen J.Y., Lim X.R., Tu T.M., Chang C.C.R., Khin H.S.W., Koh J.S., Rao J.P., Lau S.L., Tan G.B. (2021). Cerebral venous thrombosis post BNT162b2 mRNA SARS-CoV-2 vaccination: A black swan event. Am. J. Hematol..

[344] Whiteley W.N., Ip S., Cooper J.A., Bolton T., Keene S., Walker V., Denholm R., Akbari A., Omigie E., Hollings S. (2022). Association of COVID-19 vaccines ChAdOx1 and BNT162b2 with major venous, arterial, or thrombocytopenic events: A population-based cohort study of 46 million adults in England. PLoS Med..

[345] Cari L., Fiore P., Alhosseini M.N., Sava G., Nocentini G. (2021). Blood clots and bleeding events following BNT162b2 and ChAdOx1 nCoV-19 vaccine: An analysis of European data. J. Autoimmun..

[346] Sekulovski M., Mileva N., Vasilev G.V., Miteva D., Gulinac M., Peshevska-Sekulovska M., Chervenkov L., Batselova H., Vasilev G.H., Tomov L. (2023). Blood Coagulation and Thrombotic Disorders following SARS-CoV-2 Infection and COVID-19 Vaccination. Biomedicines.

[347] Grupper A., Rabinowich L., Schwartz D., Schwartz I.F., Ben-Yehoyada M., Shashar M., Katchman E., Halperin T., Turner D., Goykhman Y. (2021). Reduced humoral response to mRNA SARS-CoV-2 BNT162b2 vaccine in kidney transplant recipients without prior exposure to the virus. Arab. Archaeol. Epigr..

[348] Boyarsky B.J., Ou M.T.B., Greenberg R.S.B., Teles A.T.B., Werbel W.A., Avery R.K., Massie A.B., Segev D.L., Garonzik-Wang J.M. (2021). Safety of the First Dose of SARS-CoV-2 Vaccination in Solid Organ Transplant Recipients. Transplantation.

[349] Rahav G., Lustig Y., Lavee J., Benjamini O., Magen H., Hod T., Shem-Tov N., Shmueli E.S., Merkel D., Ben-Ari Z. (2021). BNT162b2 mRNA COVID-19 vaccination in immunocompromised patients: A prospective cohort study. eClinicalMedicine.

[350] Martinelli S., Pascucci D., Laurenti P. (2023). Humoral response after a fourth dose of SARS-CoV-2 vaccine in immunocompromised patients. Results of a systematic review. Front. Public Health.

[351] Novavax (2021). Novavax and Serum Institute of India Announce World Health Organization Grants Emergency Use Listing for NVX-CoV2373 COVID-19 Vaccine [Online]. https://ir.novavax.com/press-releases/2021-12-17-Novavax-and-Serum-Institute-of-India-Announce-World-Health-Organization-Grants-Emergency-Use-Listing-for-NVX-CoV2373-COVID-19-Vaccine.

[352] Fix J., Mast T.C., Smith K., Baker N. (2024). Benefit–risk assessment for the Novavax COVID-19 vaccine (NVX-CoV2373). Vaccine.

[353] Patel N., Trost J.F., Guebre-Xabier M., Zhou H., Norton J., Jiang D., Cai Z., Zhu M., Marchese A.M., Greene A.M. (2023). XBB.1.5 spike protein COVID-19 vaccine induces broadly neutralizing and cellular immune responses against EG.5.1 and emerging XBB variants. Sci. Rep..

[354] Food and Drug Administration (2023). Novavax COVID-19 Vaccine, Adjuvanted [Online]. https://www.fda.gov/vaccines-blood-biologics/coronavirus-covid-19-cber-regulated-biologics/novavax-covid-19-vaccine-adjuvanted#additional.

[355] World Health Organization (2021). Background Document on the Novavax NVX-CoV2373 Vaccine against COVID-19 [Online]. https://www.who.int/publications/i/item/WHO-2019-nCoV-vaccines-SAGE-recommendation-Novavax-NVX-CoV2373-background.

[356] Novavax (2024). Novavax Submits Application to U.S. FDA for Updated Protein-Based 2024–2025 Formula COVID-19 Vaccine [Online]. https://ir.novavax.com/press-releases/2024-06-14-Novavax-Submits-Application-to-U-S-FDA-for-Updated-Protein-based-2024-2025-Formula-COVID-19-Vaccine.

[357] Hotez P.J., Bottazzi M.E. (2022). Whole inactivated virus and protein-based COVID-19 vaccines. Annu. Rev. Med..

[358] Reimer J.M., Karlsson K.H., Lövgren-Bengtsson K., Magnusson S.E., Fuentes A., Stertman L. (2012). Matrix-M™ adjuvant induces local recruitment, activation and maturation of central immune cells in absence of antigen. PLoS ONE.

[359] Stertman L., Palm A.K., Zarnegar B., Carow B., Lunderius Andersson C., Magnusson S.E., Carnrot C., Shinde V., Smith G., Glenn G. (2023). The Matrix-M™ adjuvant: A critical component of vaccines for the 21st century. Hum. Vaccines Immunother..

[360] Heath P.T., Galiza E.P., Baxter D.N., Boffito M., Browne D., Burns F., Chadwick D.R., Clark R., Cosgrove C., Galloway J. (2021). Safety and efficacy of NVX-CoV2373 COVID-19 vaccine. N. Engl. J. Med..

[361] Áñez G., Dunkle L.M., Gay C.L., Kotloff K.L., Adelglass J.M., Essink B., Campbell J.D., Cloney-Clark S., Zhu M., Plested J.S. (2023). Safety, Immunogenicity, and Efficacy of the NVX-CoV2373 COVID-19 Vaccine in Adolescents: A Randomized Clinical Trial. JAMA Netw. Open.

[362] Ahmad S., Yuson C., Le A., Hissaria P. (2022). Myopericarditis following both BNT162b2 and NVX-CoV2373. Allergy Asthma Clin. Immunol..

[363] Romanson B. (2023). Notes from the Field: Safety Monitoring of Novavax COVID-19 Vaccine among Persons Aged ≥ 12 Years—United States, 13 July 2022–13 March 2023. MMWR. Morb. Mortal. Wkly. Rep..

[364] Clothier H.J., Parker C., Mallard J.H., Effler P., Bloomfield L., Carcione D., Buttery J.P. (2024). Real-world Nuvaxovid COVID-19 vaccine safety profile after first 100,000 doses in Australia, 2022–2023. medRxiv.

[365] Ogunjimi O.B., Tsalamandris G., Paladini A., Varrassi G., Zis P. (2023). Guillain-Barré Syndrome Induced by Vaccination Against COVID-19: A Systematic Review and Meta-Analysis. Cureus.

[366] Song J.-W., Hu W., Shen L., Wang F.-S. (2022). Safety and immunogenicity of COVID-19 vaccination in immunocompromised patients. Chin. Med. J..

[367] HMA-EMA Catalogues of Real-World Data Sources and Studies (2024). Safety Profile of the NVX-CoV2373 Vaccine in Individuals ≥ 12 Years of Age in the United States [Online]. HMA-EMA Catalogues of Real-World Data Sources and Studies. https://catalogues.ema.europa.eu/node/3713/methodological-aspects.

[368] Health Canada (2024). Novavax Nuvaxovid COVID-19 Vaccine [Online]. https://www.canada.ca/en/health-canada/services/drugs-health-products/covid19-industry/drugs-vaccines-treatments/vaccines/novavax.html.

[369] Mallory R.M., Formica N., Pfeiffer S., Wilkinson B., Marcheschi A., Albert G., McFall H., Robinson M., Plested J.S., Zhu M. (2022). Safety and immunogenicity following a homologous booster dose of a SARS-CoV-2 recombinant spike protein vaccine (NVX-CoV2373): A secondary analysis of a randomised, placebo-controlled, phase 2 trial. Lancet Infect. Dis..

[370] Munro A.P.S., Janani L., Cornelius V., Aley P.K., Babbage G., Baxter D., Bula M., Cathie K., Chatterjee K., Dodd K. (2021). Safety and immunogenicity of seven COVID-19 vaccines as a third dose (booster) following two doses of ChAdOx1 nCov-19 or BNT162b2 in the UK (COV-BOOST): A blinded, multicentre, randomised, controlled, phase 2 trial. Lancet.

[371] Raiser F., Davis M., Adelglass J., Cai M.R., Chau G., Cloney-Clark S., Eickhoff M., Kalkeri R., McKnight I., Plested J. (2023). Immunogenicity and safety of NVX-CoV2373 as a booster: A phase 3 randomized clinical trial in adults. Vaccine.

[372] Kutikova L., Brash J.T., Helme K., Brewster J., Brand M., Adam A., Seager S., Kostev K., Schelling J. (2024). Characteristics and Outcomes for Recipients of NVX-CoV2373: A Real-World Retrospective Study in Germany. Vaccines.

[373] Mateo-Urdiales A., Sacco C., Petrone D., Bella A., Riccardo F., Del Manso M., Bressi M., Siddu A., Brusaferro S., Palamara A.T. (2023). Estimated Effectiveness of a Primary Cycle of Protein Recombinant Vaccine NVX-CoV2373 Against COVID-19. JAMA Netw. Open.

[374] Gwak E., Choe S.A., Bolormaa E., Choe Y.J., Wang C., Fix J., Vadivale M., Rousculp M.D. (2024). Short-Term Relative Effectiveness of Homologous NVX-CoV2373 and BNT162b2 COVID-19 Vaccinations in South Korea. medRxiv.

[375] Liu B., Stepien S., Qian J., Gidding H., Nicolopoulos K., Amin J., Cheng A., Macartney K. (2023). Comparative effectiveness of four COVID-19 vaccines, BNT162b2 mRNA, mRNA-1273, ChAdOx1 nCov-19 and NVX-CoV2373 against SARS-CoV-2 B.1.1.529 (Omicron) infection. Vaccine.

[376] Lee E.S.M., Choe Y.J., Choe S.A., Gwak E.S., Kwon D.D. (2024). Relative Effectiveness of the NVX-CoV2373 Vaccine Compared with the BNT162b2 Vaccine in Adolescents. Pediatr. Infect. Dis. J..

[377] Centers for Disease Control and Prevention (2024). Updated (2023–24 Formula) Novavax COVID-19 Vaccine [Online]. https://www.cdc.gov/vaccines/covid-19/info-by-product/novavax/downloads/novavax-standing-orders.pdf.

[378] ClinicalTrials.gov (2024). A Study to Evaluate the Safety and Immunogenicity of an (Omicron Subvariant) COVID-19 Vaccine Booster Dose in Previously Vaccinated Participants and Unvaccinated Participants. (COVID-19) [Online]. https://clinicaltrials.gov/study/NCT05975060?intr=NVX-CoV2601&rank=3.

[379] ClinicalTrials.gov (2024). Phase 3 Adolescent Study for SARS-CoV-2 rS Variant Vaccines (COVID-19) [Online]. https://clinicaltrials.gov/study/NCT05973006?intr=NVX-CoV2601&rank=2.

[380] ClinicalTrials.gov (2024). Phase 2/3 Heterologous Boosting Study with Different Dose Levels of Monovalent SARS-CoV-2 rS Vaccines (COVID-19) [Online]. https://clinicaltrials.gov/study/NCT05925127?intr=NVX-CoV2601&rank=1.

[381] Singh A.V., Kayal A., Malik A., Maharjan R.S., Dietrich P., Thissen A., Siewert K., Curato C., Pande K., Prahlad D. (2022). Interfacial Water in the SARS Spike Protein: Investigating the Interaction with Human ACE2 Receptor and In Vitro Uptake in A549 Cells. Langmuir.

